# Genome-Wide Crossover Distribution in *Arabidopsis thaliana* Meiosis Reveals Sex-Specific Patterns along Chromosomes

**DOI:** 10.1371/journal.pgen.1002354

**Published:** 2011-11-03

**Authors:** Laurène Giraut, Matthieu Falque, Jan Drouaud, Lucie Pereira, Olivier C. Martin, Christine Mézard

**Affiliations:** 1 Institut Jean-Pierre Bourgin, UMR1318 INRA-AgroParisTech, INRA Centre de Versailles-Grignon, Versailles, France; 2 UMR de Génétique Végétale du Moulon, INRA/CNRS/Univ Paris-Sud/AgroParisTech, Gif sur Yvette, France; National Cancer Institute, United States of America

## Abstract

In most species, crossovers (COs) are essential for the accurate segregation of homologous chromosomes at the first meiotic division. Their number and location are tightly regulated. Here, we report a detailed, genome-wide characterization of the rate and localization of COs in *Arabidopsis thaliana*, in male and female meiosis. We observed dramatic differences between male and female meiosis which included: (i) genetic map length; 575 cM versus 332 cM respectively; (ii) CO distribution patterns: male CO rates were very high at both ends of each chromosome, whereas female CO rates were very low; (iii) correlations between CO rates and various chromosome features: female CO rates correlated strongly and negatively with GC content and gene density but positively with transposable elements (TEs) density, whereas male CO rates correlated positively with the CpG ratio. However, except for CpG, the correlations could be explained by the unequal repartition of these sequences along the *Arabidopsis* chromosome. For both male and female meiosis, the number of COs per chromosome correlates with chromosome size expressed either in base pairs or as synaptonemal complex length. Finally, we show that interference modulates the CO distribution both in male and female meiosis.

## Author Summary

Reciprocal exchanges of genetic material (crossovers) between homologous chromosomes ensure their proper segregation to generate gametes. Their number and location along chromosomes are tightly regulated. We localized precisely the position of 13,535 crossovers in more than 3,000 plants of *Arabidopsis thaliana*. While *A. thaliana* is a hermaphrodite plant with male and female meiosis occurring in the same flower and thus with the same genome, we observed dramatic differences in the distribution and the rate of crossovers along chromosomes in male and female meiosis. On average, chromosomes recombine 1.7 times more in male than in female meiosis. Moreover, male CO rates are very high at both ends of each chromosome, whereas female CO rates are very low. Finally, for the first time in a eukaryote, we show that the correlations between CO rates and various chromosome features differ in male and female meiosis. Female CO rates correlated strongly and negatively with GC content and gene density but positively with transposable elements density, whereas male CO rates correlated positively with the CpG ratio. However, most of the correlations could be explained by the structure of the *Arabidopsis* genome.

## Introduction

Crossovers (COs) are recombination events characterized by a reciprocal exchange of genetic material. In most eukaryotes, they are essential for the segregation of homologous chromosomes at the first meiotic division. When CO formation or localization is impaired, aneuploid gametes are formed [1] leading to sterility, embryo-lethality or developmental problems.

The number of COs per chromosome and per meiosis is tightly controlled. Firstly, in most species, there is a need for one obligatory CO per pair of homologous chromosomes. Secondly, interference (a lower frequency of close-by COs than expected if they were to occur independently [2]) has been shown to play a role in controlling the number of COs. The mechanism that mediates interference is still poorly understood. However, in the past few years, powerful approaches to quantify interference have been developed and applied to a number of organisms like *Arabidopsis thaliana* [3], Human [4], mouse [5] or maize [6]. The most used approaches involve the “counting” [7] and the “gamma” [8] models which parametrize the distribution of distances between successive crossovers on the bivalent. These models also give predictions for crossover patterns in gametes, and thus have been used to measure interference strength from genetic segregation data [8]. Thirdly, the distribution of COs along chromosomes is not homogeneous. In all species, the CO rate drops in centromeric regions with estimates between 5 to more than 200 fold depending on the organism [9]. COs are also rare in heterochromatic regions but the centromeric effect has been decoupled from the heterochromatic effect [10]. GC content was shown to positively correlate with the CO rate in many species such as rat, mice, human, zebra finch, honeybee and maize, even at a broad scale [11]–[14]. The underlying mechanisms responsible for this correlation are still under discussion (see [15], [16], discussion). In contrast, we reported that in *A thaliana*, the variation in CO rate of a male-female averaged map was negatively correlated to GC content [17]. Variations in CO rates also correlate with several other genomic features such as transposable elements (TE) density, the CpG ratio, gene density, nucleotide polymorphisms or chromosomal architecture properties like distance to telomeres or centromeres [11], [18]–[21]. Nevertheless, none of these other characteristics are systematically correlated with CO rate variation across every species. Thus, what causes variation in CO rates along chromosomes is still poorly understood. The various features that correlate with this non-homogeneity in CO rates may have causal relationships or may be only incidentally related.

CO rates and distribution can vary between male and female meiosis in the same species (reviewed in [22]). Haldane suggested that the heterogametic sex has a lower CO rate as a consequence of selection against recombination between the sex chromosomes [23]. However, this hypothesis, referred to as the Haldane and Huxley rule has been since called into question: less recombination in the homogametic sex than the heterogametic sex has been observed in some species and heterochiasmy (different crossover rates in male and female meiosis) has been found without the presence of sex chromosomes in plants such as *Allium* [24], *Brassica oleracea* [25] and *A*. *thaliana* [26], [27] or animals like the saltwater crocodile [28]. Other hypotheses have been proposed (reviewed in [29]) but none satisfactorily explain the variations in heterochiasmy in all species.

Strikingly, a correlation was reported between CO number per chromosome and the total length of synaptonemal complex (SC) (a proteinaceous structure that links homologous chromosomes at the pachytene stage of meiosis I [30]). Several studies have shown that CO number and SC length vary coordinately, even in situations where DNA length is constant [31]. For example, in human meiosis, males have about half the CO number and total SC length compared to females [32]. This correlation was also reported in male and female meiocytes in *Dendrocoelum lacteum* [33] and zebrafish [34], and in many other species with various individuals of the same population [35]–[37]. The reasons for this correlation are still poorly understood.

*A. thaliana* has a comparatively small genome estimated to be between 125 and 157 Mb at the haploid stage [38], [39]. DNA is distributed on 5 pairs of chromosomes. Chromosomes 2 and 4 are acrocentric and carry on the telomeric half of their short arm several hundreds of copies of rDNA 18S, 5.8S and 25S constituting the Nucleolar Organizer Regions (NORs) [38]. Thus, including the NOR, their size is approximately between 22 and 25 Mb. Chromosomes 1, 3 and 5, are metacentric. Their sizes vary from 19.7 to 30.4 Mb (http://www.ncbi.nlm.nih.gov/mapview/).

A few years ago, we published detailed genetic maps of *A. thaliana* chromosome 4 [17], [27]. The first map was built by genotyping progeny obtained after self-fertilization of an F1 between the two accessions Columbia and Landsberg *erecta*. It determined the sex-averaged distribution of COs along chromosome 4. This sex-averaged distribution pattern was found to be highly non-homogeneous with successive regions of high and low CO rates. Regions with significantly higher CO rates had a high CpG ratio and low GC content. For the second map, the same F1 Columbia x Landsberg *erecta* was used either as male or a female in a backcross with Columbia. By genotyping the progeny of these two crosses on chromosome 4, we demonstrated that male and female CO rates were dramatically different, with a male/female ratio of 1.64. Positive interference was also found both in male and female meiosis. The CO distribution contrasted too between male and female meiosis, with very high male CO rates at both ends of the maps while at the same locations female CO rates were either average or below average. A similar ratio between male and female meiotic CO rates was reported recently using the same parental accessions but the limited number of meioses studied (137 female and 92 male) did not allow precise comparison of the CO distribution between male and female meiosis [40].

To determine if the CO landscape found in male and female meiosis was peculiar to chromosome 4, we decided to perform the analysis of CO rates and distributions along all five chromosomes of *A. thaliana*. Moreover, we strengthened the analysis by investigating the correlations between CO rates and several genomic features genome wide in both male and female meiosis separately. Finally, we performed a quantitative analysis of the interference strength using the gamma model.

## Results

F1 plants from crosses between Columbia (Col) and Landsberg *erecta* (Ler) were backcrossed with Col plants using the F1 either as the male or the female parent, thereby creating two populations. On average for each marker 1,505 plants were genotyped with 380 SNPs in the male population and 1,507 plants with 386 SNPs in the female population (380 in common between the two populations) (Table S1), spanning the five chromosomes (see Materials and Methods). With such small intervals (having an average of 316 kb in male and 311 kb in female with maximum of up to 3.2 Mb; Table S2), double COs are expected to be negligible, and so we calculated genetic distances between adjacent markers simply by dividing the number of recombinant chromosomes by the total number of plants genotyped at both markers (Table S2).

The CO landscapes obtained on chromosome 4 in male and female did not differ from those obtained in our previous study generated with the same parental accessions and the same set of markers (lowest *p*-value  = 0,14; see Materials and Methods). We therefore confirmed that there is no significant variation in meiotic CO rate for a given genetic background.

### Segregation bias in the population arising from male meiosis

**Figure 1:**
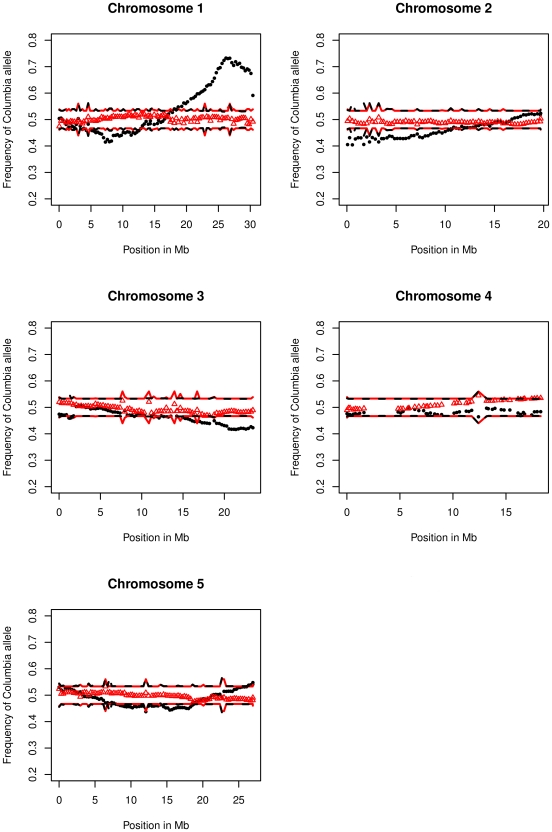
Frequency of Columbia alleles at each marker for the five chromosomes. Black dots: male population. Red triangles: female population. Black dashed and red solid lines represent the 99% confidence interval for the “no distortion” hypothesis in the male and female populations, respectively (see Materials and Methods).

For the five chromosomes, the average frequencies of the parental alleles at each marker locus were examined. In the male population we found regions of the genome with a significant segregation distortion at *p*-values less than 0.01, *i.e*, regions where the observed genotypic frequencies departed from the 1∶1 ratio predicted if no selection bias occurred during the generation of populations (Figure 1). No significant departure from normal ratios was detected in the female population. Thus all the observed cases of segregation bias are likely to be linked to a problem in the male gametophyte. The strongest segregation distortions were detected on chromosome 1 with values up to 1.49∶1 (Ler:Col) and 2.70∶1 (Col:Ler) at position 7,267,270 and 26,188,466 respectively (Figure 1). This is consistent with the hypothesis of two genes under selection, with a preference for the Ler allele at the first position and Col at the second position. The segregation bias affects the estimates of recombination rates, in particular for markers located between the two selected genes. To calculate this effect, we estimated the relative fitness of the Col:Ler alleles, 0.67∶1 at position 7,267,270 and 1∶0.37 at position 26,188,466 (see Materials and Methods). We then determined the CO rates for the 60 intervals between the two markers, correcting for the bias produced by the selection. We found that instead of the 78 cM estimated with our genetic map between the two selected markers, the corrected distance was 95 cM. Thus for the whole chromosome 1 male, instead of 142 cM, the corrected length is 159 cM. On the other chromosomes, the segregation distortion was too small to have a significant impact on CO rates, distributions or correlation analyses.

### Chromosomes recombine at a higher rate in male than female meiosis

We obtained 13,535 COs in our two populations, 8,532 and 5,003 in male and female meiosis respectively. This difference was highly significant (chi2 test, *p* = 1.2 e-202). On average, there were 11.15 and 6.6 COs per male and female meiocyte respectively (Table 1). Thus, the genetic map length for male meiosis was 575 cM (1 cM per 209 kb on average) and 332 cM (1 cM per 361 kb on average) for female meiosis. The global male to female CO ratio was 1.73. This ratio was similar to the ratio of male to female total SC length (1.69) obtained in the same genetic background [27].

**Table 1:** Comparison between male and female population.

	Female	Male	Ratio Male/Female
Number of COs analyzed	5003	8532	
size genetic map (cM)	332	575	
COs per cell	6.65	11.15	1.67 (1.63–1.70)*
COs per chromosome 1 bivalent	1.63	2.85	1.75 (1.70–1.81)*
COs per chromosome 1 bivalent corrected**	1.63	3.18	1.95 (1.90–2.01)*
COs per chromosome 2 bivalent	1.19	1.89	1.58 (1.52–1.65)*
COs per chromosome 3 bivalent	1.29	2.14	1.66 (1.60–1.72)*
COs per chromosome 4 bivalent	1.10	1.71	1.56 (1.49–1.62)*
COs per chromosome 5 bivalent	1.44	2.58	1.79 (1.73–1.85)*

*Parentheses indicate 95% confidence intervals of the Male/Female ratio (See Methods).

**Values for chromosome 1 Male bivalent are given both with and without correcting for the segregation bias (See Methods)

**Figure 2:**
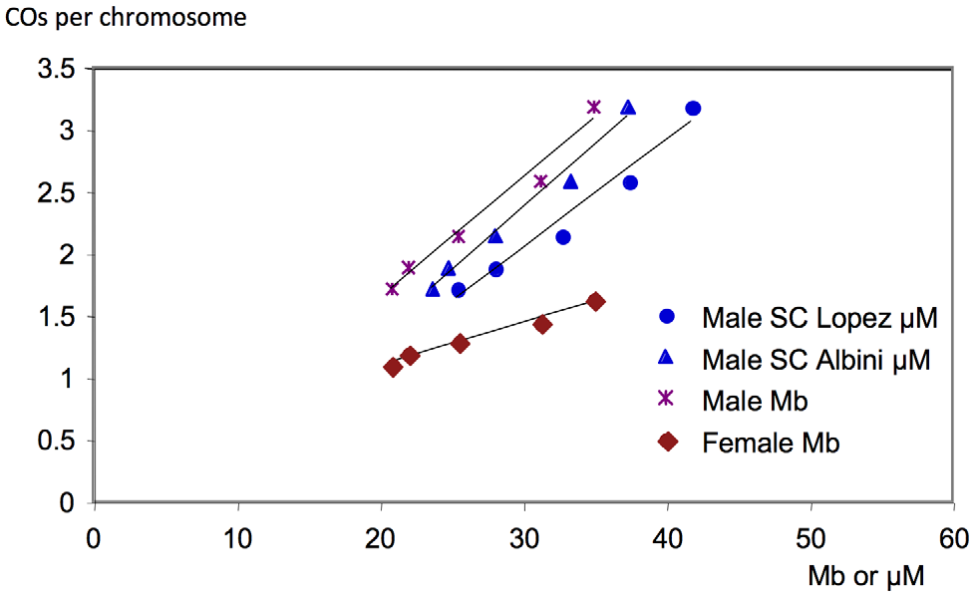
Correlation between the number of COs per chromosome and the physical size of chromosomes. Number of COs per chromosome at the bivalent level *versus* size of a chromosome in Mb or the size of the synaptonemal complex of a chromosome in µM. Blue dots: data from [42]. Blue triangles: data from [41]. In the case of our male and female genetic map data, numbers of COs at the bivalent level were obtained by doubling the genetic size in Morgans. Male SC Lopez: y = 0.0869x−0.5776; R^2^ = 0.97. Male SC Albini: y = 0.1016x−0.36394; R^2^ = 0.98. Male Mb: y = 0.097x−0.3022; R^2^ = 0.98. Female Mb: y = 0.0342x+0.4068; R^2^ = 0.98

We then compared male and female CO rates at the level of the bivalent (pair of homologous chromosomes) (Table 1). For male meiosis, the mean number of COs per cell varied between 1.7 for chromosome 4 bivalent (the smallest) to 3.2 COs for chromosome 1 bivalent (the longest). In female meiosis, fewer COs were found per chromosome with 1.1 on chromosome 4 bivalent up to 1.6 on chromosome 1 bivalent. For both male and female meiosis, a linear correlation was observed between the size of chromosomes in Mb and the average number of COs per chromosome (Figure 2) (*R^2^* = 0.98 in M and in F) but with a different slope. For male meiosis, we also analyzed the correlation between the number of COs per chromosome and the size of the SC in µM for each chromosome obtained in two different studies [41], [42]. We again obtained a linear correlation (Figure 2). This is expected given the close proportionality between SC length in µM and physical length in Mb for male meiosis (R^2^>0.999, data not shown).

In conclusion, the five chromosomes undergo more COs in male meiosis than in female meiosis and this difference becomes more substantial when the physical length of the chromosome is greater.

### Distributions of CO number per chromosome in male and female meiosis are not random, suggesting interference

**Figure 3:**
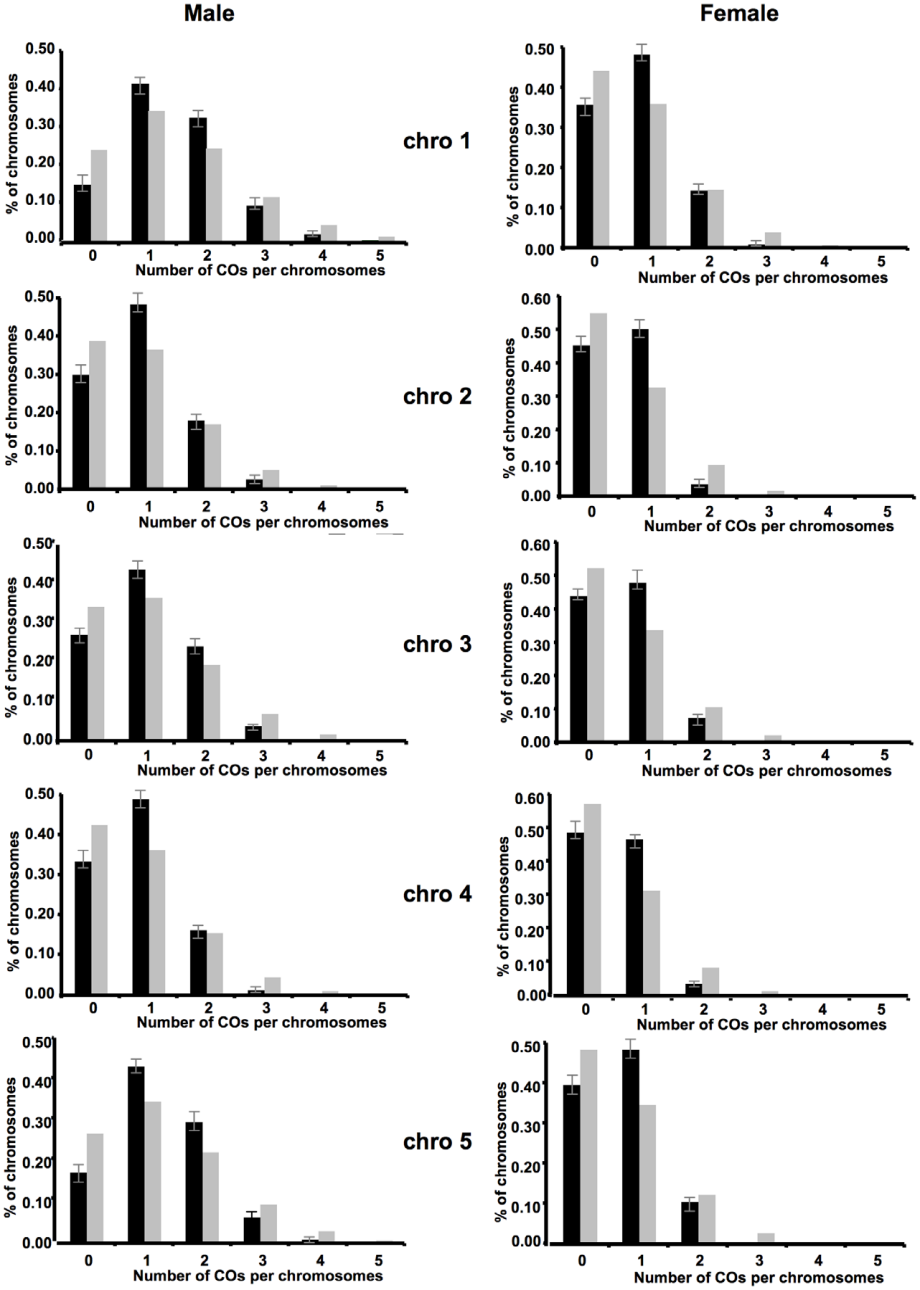
Distributions of CO numbers per chromosome in male and female meiosis. Observed and Poisson distributions are shown in black and grey respectively. *p*-values are obtained by testing the hypothesis that the experimental data are Poisson-distributed. Error bars on observed distributions indicate 95% confidence intervals. *p*-values <1.6 e-20 for all of the comparisons.

We looked at the distributions of CO numbers per chromosomes in male and female populations (Figure 3, Table S4). In the hypothesis of non-interfering COs, their numbers per chromosome are distributed according to a Poisson law of mean given by the genetic length. We thus calculated the theoretical distribution for each chromosome using the measured average number of COs. As readily seen in Figure 3, for all five chromosomes, both in male and female meiosis, the observed and the expected (Poisson) distributions show clear differences (all *p*-values <10^−19^). In all cases, there is a deficit in plants with no CO and an excess of plants with one CO compared to the Poisson distribution.

As an illustration, in the female population, only 8.6% of plants had more than one CO whereas 14.4 were expected in the absence of interference. This effect was particularly obvious on the small chromosomes 2 and 4 where only 4.4% and 3.9% of plants had multiple COs while 12% and 10.6% were expected respectively.

Thus we observed a decrease of events with no or many COs, and excess of events with one CO which reduces the variance of CO number per chromosome, as predicted as a consequence of interference (see Discussion).

Interference reduces the variance in the number of crossovers but also the variance in the distance between adjacent crossovers. Thus we measured the interference intensity by fitting the gamma model to estimate its parameter *nu* (95% confidence intervals indicated in brackets). In male meiosis, for the successive five chromosomes, we have (starting from chromosome 1 to 5) 2.6 [2.4–2.9], 2.5 [2.2–2.8], 2.5 [2.2–2.7], 3.5 [3.1–4.0], and 3.0 [2.7–3.3]. Similarly, in female meiosis, we have 2.7 [2.4–3.1], 2.8 [2.4–3.3], 2.6 [2.2–3.0], 4.1 [3.3–4.9], and 3.5 [3.0–4.0]. In all cases, the hypothesis of no interference, corresponding to *nu* = 1, is excluded.

### The CO distribution on the five chromosomes differs in male and female meiosis

**Figure 4:**
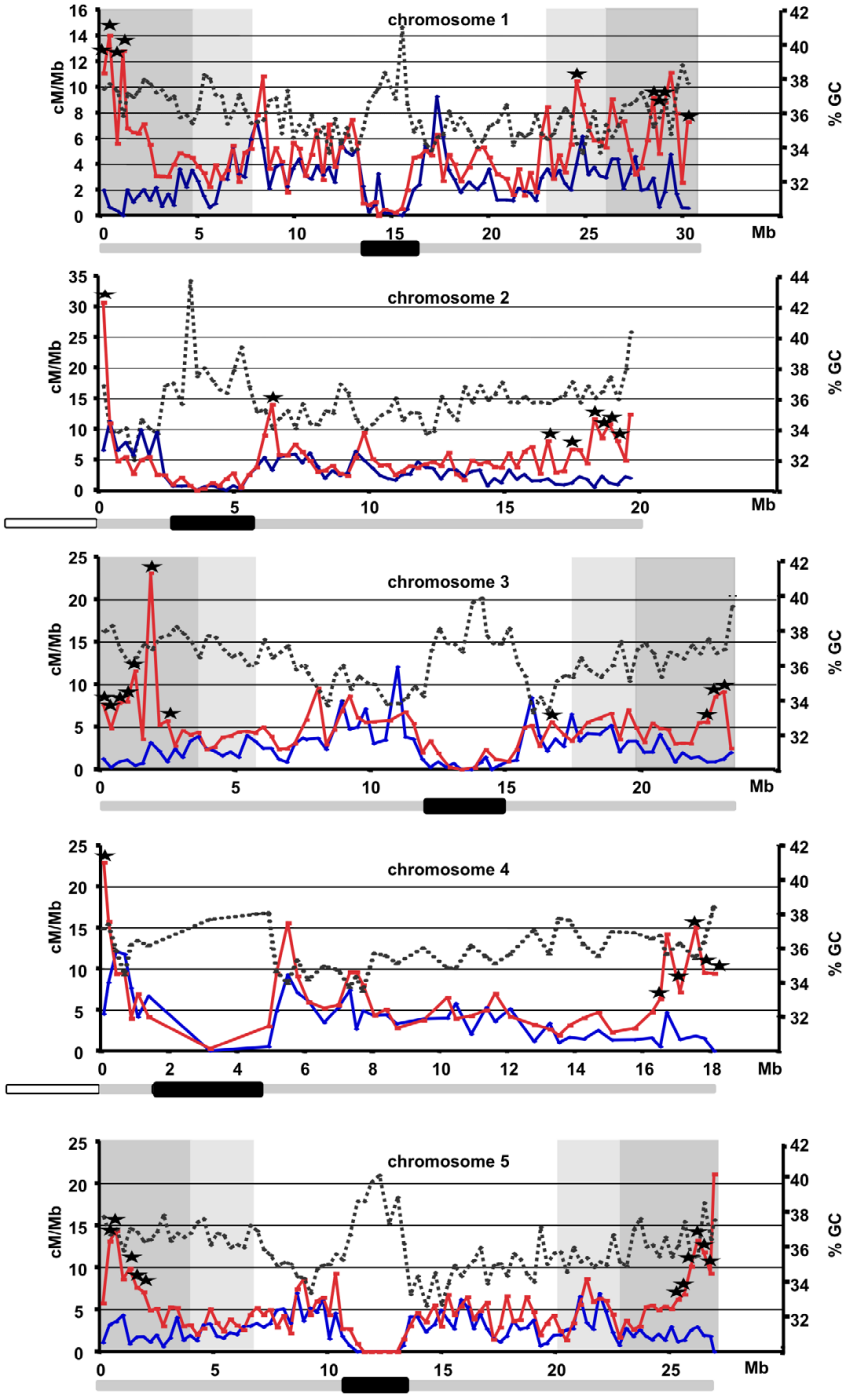
CO distribution and GC% along the five chromosomes. Blue line: CO rates in female meiosis. Red line: CO rates in male meiosis. Dotted black line: GC%. Intervals with significantly different CO rates between male and female meiosis are indicated with black stars (*p*<0.05; Benjamini 0.05). Regions of chromosomes 1, 3 and 5 removed in the "−30%" and in the "−50%" analyses are shown in dark and light grey respectively. In the bar under each graph, the black box corresponds to heterochromatic regions, and the white box corresponds to the NOR regions.

In the male population, the CO rate per interval varied from 0 to 30 cM/Mb and in the female population from 0 to 12 cM/Mb. Strikingly, visual examination of the graphs suggested that the regions with the most contrast between CO distribution in male and female meiosis were the terminal regions of the chromosomes (Figure 4). To analyze these differences in more detail, in the male and female populations, we compared the CO rate of each interval for the five chromosomes to the "mean" CO rate of each chromosome arm (excluding the centromeric heterochromatic regions, see Materials and Methods, Table S2, Figure 4). For each chromosome, both in male and female meiosis, we observed a number of "hot" (40 and 32 in male and female populations respectively) and "cold" (80 and 73 in male and female populations respectively) intervals (Table S2). (An interval was considered to be "hot" or "cold" when the 95% confidence of its CO rate did not contain the mean CO rate of the considered arm (Table S2; see Materials and Methods)). Indeed, in the male population, 27/40 of the "hot" intervals were located in the telomeric third of the arms of the chromosomes and the remaining ones were mainly localized in the pericentromeric area. Conversely in the female population, only three out of the 32 "hot" intervals were located in the distal third of the chromosomes while most of the others were pericentromeric. For the "cold" intervals, the proportions were inversed with 15/65 in male meiosis located in the distal area and 58/73 in female meiosis. Interestingly, only two "hot" intervals were shared while 27 "cold" were common between male and female populations.

In a pairwise comparison, 46 intervals were significantly different between male and female populations (see Materials and Methods, *p*<0.05). Not surprisingly, the vast majority of these intervals (43/46) were located at the ends of the chromosomes (Figure 4). This led us to ask if the observed differences in global CO rates per chromosome between male and female meiosis were only due to the intervals at chromosomal ends. We thus compared the male and female genetic length of each chromosome when removing intervals belonging to the ends. Explicitly, we considered two cases, the first where 30% of the physical length was removed (thus 15% of the total length on each end), hereafter referred to as “−30%”, and the second where 50% was removed, hereafter referred to as “−50%”. Genetic intervals overlapping these truncated regions were entirely removed. Map lengths were computed by counting recombination events using all markers rather than only adjacent markers, to overcome the limitations coming from missing data. We did not include the two small chromosomes 2 and 4 in this analysis because of their peculiar structure: the terminal end of their short arm consists of several megabases (3 to 6) of the sequences of the nucleolar region (NOR) for which we do not have markers. Thus we could not look at the effect of the chromosomal end on CO rates on these two chromosomes. By taking away the genetic intervals corresponding to 30% of the physical length, we eliminated 30 of the 32 intervals with significant different CO rates, whereas taking away 50% of the physical length kept only 1 significant interval, on chromosome 3. We analyzed the effect of the truncations on the distribution of chromosomes with 0, 1, and 2 or more COs. We found that the observed fraction lost is higher than expected for individuals with 2 or more COs and less for those with one CO (Table S5). Thus, the truncation indeed penalizes more severely the individuals with many rather than few COs. The deviations from the expectation are modest, and they are also unsurprising since there is positive interference, so for instance individuals with 2 COs have these COs more frequently in the extremities.

On all three chromosomes 1, 3, and 5, and for both truncations (“−30%” or “−50%”), the male genetic map remained longer than the female one. The male to female ratio decreased as the number of intervals kept in the analyses was reduced, for instance in the case of chromosome 1 from 1.75 (all intervals,) to 1.38 (“−30%”) and to 1.33 (“−50%”). The other chromosomes showed the same trend (Table 2). In spite of this trend, the male/female differences remained highly significant (*p*-value <10^−8^). Thus, even though an important part of the differences between male and female genetic maps is due to the intervals at the sub-telomeric ends, chromosomes recombine more in the central part in male meiosis than in female meiosis (Table 2).

**Table 2:** Comparison of male and female genetic map length with truncated chromosomes.

				
Genetic size		Total	−30%	−50%
**Chro1**	Male	142.36	81.92	53.84
	Female	81.29	59.26	40.54
	Male/Female	1.75	1.38	1.33
**Chro3**	Male	106.78	70.38	52.08
	Female	64.30	51.23	36.36
	Male/Female	1.66	1.37	1.43
**Chro5**	Male	128.95	71.48	50.07
	Female	71.90	54.21	38.17
	Male/Female	1.79	1.32	1.31

“−30%”: correlation based on a truncated chromosome where 15% of the physical length at each extremity was removed. “−50%”: correlation based on a truncated chromosome where 25% of the physical length at each extremity was removed. *p*-value: probability that the observed discrepancy between Male and Female genetic map sizes be as large as it is under the hypothesis that their true values do not differ. *p*-value <4.81 e-09 for all of the comparisons.

Seven out of eight of the intervals found to be significantly different between male and female on chromosome 4 in our previous study [27] were also retrieved in this study. The eighth interval was the least significantly different in the previous study and was borderline in this study. Our CO map thus looks robust in this genetic background.

In conclusion, during both male and female meiosis the CO distribution is not homogeneous along the chromosomes and these distributions exhibit very contrasting patterns between the male and female populations. Moreover, even if the telomeric regions, which showed the greatest contrast, are removed, the lengths of the remaining genetic maps are still significantly different between male and female meiosis.

### CO rates correlate with different genomic features associated with the structure of the *Arabidopsis* chromosomes

In a previous study, we reported that high CO rates in a sex-averaged F2 population correlated positively with the CpG ratio but negatively with the GC content [17]. Simple repeats only gave a weak positive correlation and all the other parameters tested (TE density, gene density, pseudogene density) did not show a correlation. We repeated similar analyses with our separate male and female CO maps here.

**Figure 5:**
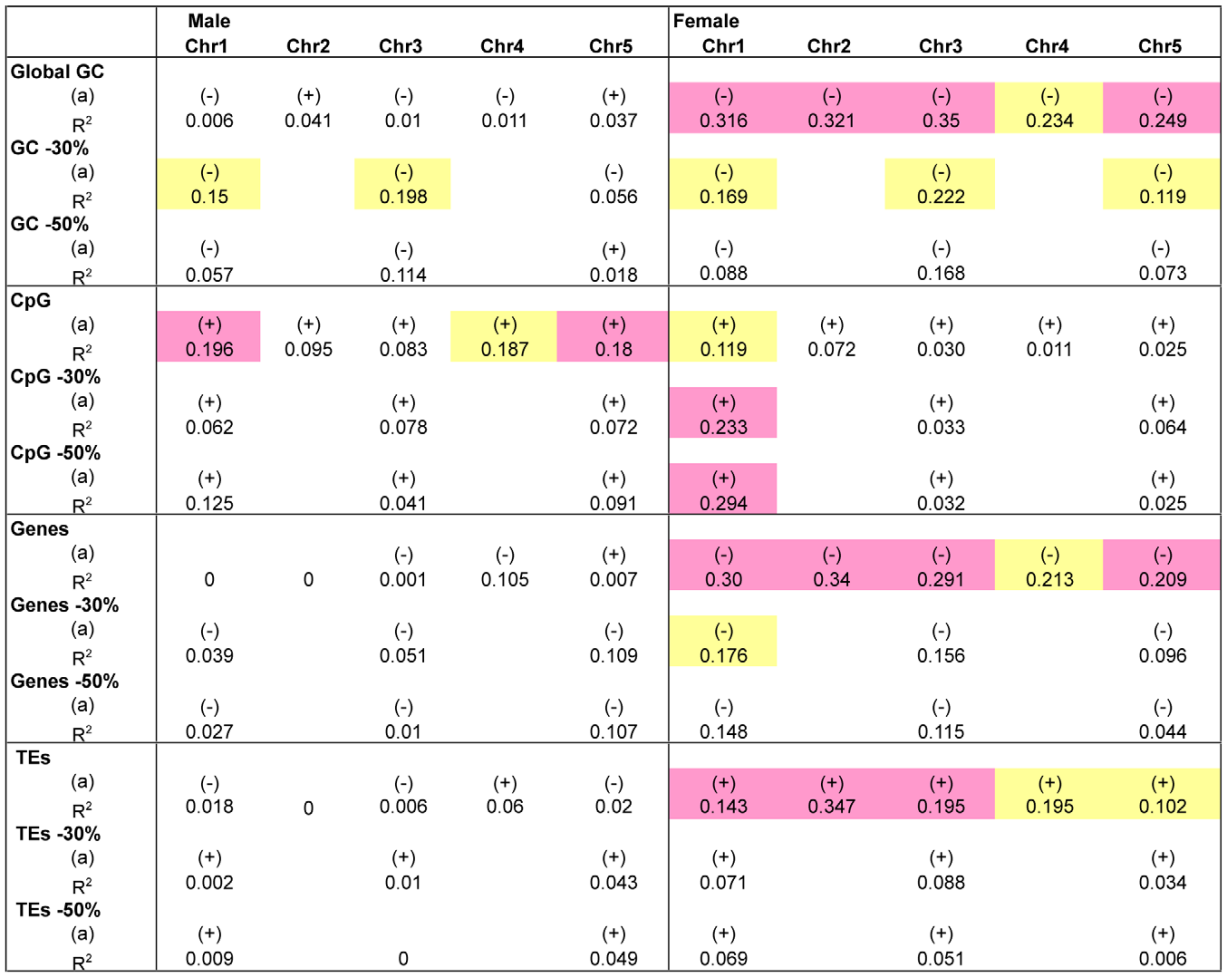
Correlation between recombination rate in male and female meiosis and several genomic features. Global GC: Proportion of G or C nucleotides in the whole interval. CpG: ratio between the number of CpG or GpC dinucleotides over the length of the sequence in the interval. Genes: proportion of bases which belong to a gene. TE: proportion of bases which belong to a transposable element. (a): direction of the correlation. yellow: *p*-values below 10^−2^. pink: *p*-values below 10^−3^

Strong correlations (*p*-value <10^−3^) were found in the female population. CO rates for all chromosomes correlated negatively (chromosomes 1, 2, 3, and 5 strongly and chromosome 4 weakly [10^−2^< *p*-value <10^−3^]) with GC content and gene density (Figure 5, Figure S1). For TEs, the correlation was the other way around: positive and strong for chromosomes 1, 2 and 3, weak for chromosomes 4 and 5. It has to be noted that the R values found were in the same range than those published in sex averaged studies for human, honey bee or zebra finch [11]–[13], [43]. No significant correlations were found for any of the five chromosomes in the male population of *Arabidopsis* for these three parameters. On the other hand, in the male population, for chromosomes 1 and 5, recombination rates correlated strongly (weakly for chromosome 3) and positively with the CpG ratio, while in the female population, only chromosome 1 correlated weakly (Figure 5).

We tested if these differences in the strength of the correlation were mainly due to the telomeric intervals. We reanalyzed the correlations on chromosome 1, 3 and 5 in the “−30%” and “−50%” cases, as was done in the comparison of the size of the genetic maps in male and female meiosis (see above). In the “−30%” case, clearly, in female meiosis, the strength of all the correlations between CO rates and GC%, gene density and TEs were weakened. Moreover, they all disappeared in the “−50%” case (Figure 5; Figure S1). *A contrario*, no change was observed on the male side.

These results prompted us to look at the GC content, the genes and the TE density along the arms of chromosomes. In fact, these three features exhibit a significant gradient, negative for TEs and positive for genes and GC% from the centromeric to the telomeric end for all chromosome arms except the short arms of chromosomes 2 and 4 (correlation *p*-values <10^−4^; Figure S1). Thus, the correlations between GC%, gene and TE densities and the recombination rate in female meiosis, and the fact that this correlation disappeared when the telomeric intervals were removed from the analysis, could be mainly due to the distribution of these features along the chromosomes.

A similar analysis was conducted with CpG ratio. Surprisingly, the weak correlation found in female meiosis on chromosome 1 strengthened in the “−30%” and “−50%” cases but, in male meiosis, all the correlations disappeared (Figure 5; Figure S1). There is no significant variation in CpG distribution along the chromosomes and thus the weakening of the correlation cannot be attributed to the architecture of this feature along the chromosomes (Figure S1).

Finally, no correlation was found between recombination and either coding GC, GC1, GC2, or GC3 (G or C in position 1, 2 or 3 of a codon) in both male and female meiosis (Figure S1; Table S3).

## Discussion

We obtained a very detailed genetic map of the five *Arabidopsis thaliana* chromosomes in male and female meiosis. For this we genotyped 380 and 386 markers on 1,505 and 1,507 plants in a male and female population, respectively, derived from a backcross of an F1 Col×Ler with the parent Col. We previously reported sex-related variations in CO rates and distribution on chromosome 4, the smallest of the five chromosomes [27]. With the present study, we extend this observation to the five chromosomes and we report marked differences between CO rates and various genomic features between male and female meiosis. Moreover, we provide a quantitative analysis of crossover interference strength.

### Co-variation between genetic length and physical length of chromosomes

In male meiosis, the mean number of COs per chromosome varies linearly with the length of the SC. Moreover, we found that the ratio of the male *vs* female genetic map length is comparable to the ratio of the total length of the SC in the same genetic background in male and female meiosis (1.69; [27]). CO rates and SC length have been shown to co-vary in several species including human, mice, *Drosophila*, and zebrafish (reviewed in [31]). The exact nature of this relationship remains unknown but recent data gave new insight into our understanding of this observation. In *C. elegans*, a mutation in a gene coding for a subunit of condensin modifies both the length of the SC and the CO rate [44]. Note that the length of the axes is modified even in the absence of the DNA double strand breaks that initiate meiotic recombination. Hence, it is tempting to suggest that the length of the SC determines the number of COs.

However, in various species, it has been reported that, proportionality between genetic and SC length was generally observed for long but not for short chromosomes. In various species such as in yeast, dog, mouse, or pigeon, small chromosomes often have a higher density of COs [45]–[48]. It has been hypothesized that this observation reflects the rule of the "obligatory" CO where one CO must occur per pair of homologous chromosomes to ensure their proper segregation at the first meiotic division [5], [49], [50]. In mammals, it has been found that the number of chromosome arms is a better predictor of CO numbers suggesting that, especially for metacentric chromosomes, one CO per chromosome may not be sufficient for the correct segregation of homologous chromosomes [51], [52]. However, two different studies suggest that the model “at least 1 CO per chromosome” rather than per arm has a better fit with human data [53], [54]. We did not observe a higher density of COs on short compared to long chromosomes in *A. thaliana*. However, there is not much size difference between *Arabidopsis* chromosomes (30.4 Mb for the longest and 18.6 Mb for the smallest) compared to other organisms where large differences have been observed such as mice (197 Mb and 61 Mb) or *S. cerevisiae* (1,5 Mb and 320 kb) (http://www.ncbi.nlm.nih.gov/mapview/).

In male meiosis, the linear fit between CO number per chromosome and chromosome size is equally good in Mb or µM of SC. This is expected since we found a very clear proportionality between SC length in µM and physical length in Mb. [38]. We observed that the male/female CO ratio differs significantly between long and short chromosomes. Long chromosomes have a higher ratio than short chromosomes. Once again, it could be an effect of the obligatory CO. All chromosomes under a certain threshold size, estimated to be 17.3 Mb in female and 13.9 Mb in male, would undergo only the obligatory CO, giving a M/F ratio of one. Above this minimal size, there would be an increase in CO number proportional to the chromosome length but in a different way in male *versus* female meiosis. Such a hypothesis corresponds to the following formula: *L_G_* = 0.5+a(*L_Mb_*−*L_thr_*) where *L_G_* is the genetic size in Morgans (half the average number of COs per bivalent), *L_Mb_* is the physical size in Mb, and *L_thr_* is a threshold physical size. This is similar in spirit to the model proposed by Li and Freudenberg [55], in which *L_thr_*  = 0. For completeness, we have fitted that particular model to our male and female set of data, obtaining p-values below 10^−13^ in female meiosis and 10^−39^ in male meiosis. Thus our data do not support that model at all. This was not unexpected because we know that there is positive CO interference in *Arabidopsis*, so the relationship proposed by Li and Freudenberg [55] should become non-linear as the genetic size approaches 50 cM.

### Interference strength seems not to vary between chromosomes

We have also confirmed that both in male and female meiosis, the distributions of CO number per chromosomes are not random. Similar results were also found by Toyota et al [40]. However, in their study, neither chromosome 4 during pollen formation in early flowers nor chromosome 5 during pollen formation in late flowers exhibit an observed CO distribution per chromosome significantly different from a Poisson distribution. This discrepancy could probably be explained by the limited number of meioses studied (92 and 93 respectively).

We found that the variance of the number of COs is smaller than would be expected under the hypothesis of no interference. Further analyses using the gamma interference model confirmed that the estimated interference parameter *nu* is always significantly higher than 1 (expected value without interference) for all chromosomes in male and female meiosis. This is not surprising since positive interference has been previously reported in *A. thaliana* (reviewed in [56], [57]). The parameter *nu* is a measurement of interference strength which does not depend on interval sizes (as opposed to coincidence coefficients), and may be easily related to the parameter *m* of the counting model [7] by the relation: *nu*  = *m*+1 when *nu* is an integer. The values of *nu* estimated in the present paper range between 2 and 5, and we do not observe any significant difference in interference strength between chromosomes, or between male and female meiosis, based on the 95% confidence intervals. Our values for *nu* are similar to previous results on *A*. *thaliana* [3] obtained with comparable methods, but in the latter case, sample sizes were smaller and no confidence intervals were given. *nu* has been found to vary between species. For tomato chromosomes 1 and 2, Lhuissier et al. [58] found *nu* = 7.9 and *nu* = 6.9 based on MLH1 immunolocalization along the synaptonemal complex. In mouse, similar methods indicated *nu* = 7.5 and *nu* = 10.1 for chromosomes 1 and 2 [59]. Estimates of *nu* were also obtained in dog (6.5 [46]), cat (3.7, [60]), and shrew (11 to 16 [60]). However, the mechanisms underlying the variations of *nu* are not understood.

### CO distribution along the chromosomes varies between male and female meiosis

We confirmed that the sex-related difference in CO distribution previously identified on chromosome 4 is a characteristic of all five chromosomes [27]. In male meiosis, CO rates are very high at both ends of the chromosomes and high on proximal parts of chromosome arms. On the other hand, female CO rates are high on proximal regions but very low at the telomeric ends of the chromosomes. This pattern is very similar to the male-female CO distribution observed in humans with the noticeable difference that in human the CO number ratio is the opposite: 1.8 more COs in female than male [61]. In humans, it was suggested that COs arise in regions that initiate synapsis in prophase I of meiosis [62], [63]. However, during *Arabidopsis* male meiosis, synapsis initiates at many sites along the chromosomes including those in the terminal part [64]. Some of these sites coincide with the future localization of COs but synapsis initiates also at loci that will not be involved in reciprocal exchange. A similar situation has also been reported in other plants (discussed in [64], [65]). Our results would suggest that there is an additional level of control of CO distribution other than the constraints imposed on synapsis initiation.

We confirmed that the difference in the size of the genetic maps between male and female meiosis first observed on chromosome 4 holds true for the 5 chromosomes. The average male/female ratio is 1.73. A similar ratio was reported in a recent study [40]. When the most contrasting intervals for recombination located at the telomeric intervals were removed, the sizes of the genetic maps were still significantly different between male and female meiosis. Thus these telomeric intervals are not sufficient to explain the differences in CO rates per chromosome. It suggests that all along the chromosomes, COs are more prone to occur on a male chromosome than on a female chromosome. However, the biological reasons of these differences are still unknown.

### CO rates do not correlate with GC content along *Arabidopsis* chromosomes

We previously reported that CO rates correlated negatively with GC content and positively with the CpG ratio on chromosome 4 but no correlation was found with genes or TE densities. However, that analysis was done only for chromosome 4 and only with a sex-averaged genetic map. In this present study, we readdressed this issue using our male and female CO maps on all five chromosomes. We found correlations mainly in female meiosis. Female CO rates correlated strongly and negatively with GC content and genes density but positively with TEs density. All these correlations weakened and/or disappeared when telomeric intervals were removed from the analysis. We observed that TEs, genes and GC% have a specific location along the chromosome arms (Figure S1). They all exhibit a significant gradient from centromeres to telomeres, positive for genes and GC% and negative for TEs. Therefore, it is tempting to suggest that the observed correlations in female meiosis could be indirect due to the specific distributions of these features along the arms of the chromosomes.

Under the hypothesis of a positional effect between CO rates and chromosomes features, our data suggest that meiotic CO rates and GC% are not correlated in *Arabidopsis*, in either male or in female meiosis.

However, when previously studied in several species, CO rates and GC% were always reported to be positively correlated [11]–[14], [18], [43], [66], [67].On the other hand, in human, Kong et al [43] noticed that the correlation became negative when the CpG ratio was included in a multiple regression model. Moreover, when the strength of this correlation was studied at different scales, such as in *S. cerevisiae* and humans, it was shown to be very strong at a fine scale (5 kb in yeast, 15 to 128 kb in human) and to weaken dramatically at a broad scale (30 kb in yeast or 1 Mb in humans) [16], [66], [68] suggesting that the relationship could be complex. The cause of these correlations is still under debate. It has been suggested that recombination could shape genome evolution through a process called biased gene conversion (BGC) [67], [69]. BGC refers to two possible mechanisms: mismatches created during the recombination process could be more frequently repaired towards GC leading to an increased probability of fixing GC alleles [70]; alternatively, the allele containing the least GC may initiate DSBs more frequently and be thus repaired by the GC-rich allele [71]. The former hypothesis is well supported by recent analysis in human [15] but *at contrario*, in *S. cerevisiae* where GC content is not driven by recombination [16]. The high level of inbreeding in *A. thaliana* populations, (outcrossing has been estimated at around 1% but could reach 14.5% in some populations ([72]) has been suggested to attenuate the effect of BGC [18] and could explain why no correlations were observed in our analysis.

In conclusion, our study provides a detailed survey of the CO landscape in male and female meiosis in *Arabidopsis thaliana*. We detected very specific sex-related patterns along the five chromosomes that highlight new differences between male and female meiosis.

## Materials and Methods

### Plant material

The *Arabidopsis thaliana* accessions “Columbia-0” (Col)(186AV), “Landsberg *erecta*” (Ler)(213AV), were obtained from the “Centre de Ressources Biologiques” at the “Institut Jean Pierre Bourgin”, Versailles, France (http://dbsgap.versailles.inra.fr/vnat/).

The Col accession was crossed to Ler to obtain F1 hybrids. Col plants were then crossed with an F1 hybrid used either as the male (Col×(Col×Ler)) or as the female ((Col×Ler)×Col). Seeds from these crosses were sown *in vitro*, and then, after two weeks seedlings were grown in a greenhouse under standard conditions for three weeks. After three weeks, whole plants were collected in 96 well plates and freeze-dried.

### DNA extraction

For the (Col×(Col×Ler)) and ((Col×Ler)×Col) populations, plant material was lyophilized then ground in 96 well plates with wells hermetically closed with plastic caps. 1 ml of Extraction Buffer (Tris pH 8 0.1 M, EDTA 50 mM, NaCl 0.5 M, SDS 1.25%, PVP 40 000 1%, Sodium Bisulfite 1%, pre-warmed at 65°C) was then added to each well and the plates were incubated at 65°C for 30 min. 300 µl of cold 60% K Ac 3 M, 11.5% glacial acetic acid was added to each well. The plate was sealed with a Thermowell film (Corning), shaken gently and placed on ice for 5 min. After centrifugation in a A-4-62 rotor (Eppendorf) at 3,220 g and 4°C for 10 min, 800 µl of the supernatant was transferred to a clean DeepWell plate and 1 mL of CGE buffer (1/3 Guanidine hydrochloride 7.8 M, 2/3 ethanol 96%) was added per well. 600 µL of the mixture was filtered with a Whatman Unifilter 800 GF/B plate placed on Deep Well plate (Greiner bio-one) and centrifuged for 2 min at 5,806 g in a Nr 09100F rotor (Sigma) at room temperature. The flow-through was discarded. This step was repeated twice. The membrane was washed twice by adding 500 µl of Washing buffer (37% Aqueous solution, 63% ethanol 96%) (Aqueous solution: K Ac 160 mM, Tris HCl pH 8 22.5 mM, EDTA 0.1 mM) and then centrifuged for 2 min at 5,806 g at room temperature. The DNA was eluted with 70 µl of H_2_O by centrifugation for 2 min at 363 g at room temperature. This step was repeated once. RNAse A was added to 0.5 µg/ml and the DNA concentration was determined using the Quant-iT dsDNA BR assay Kit (Invitrogen) with an ABI 7900HT real-time PCR system (Applied Biosystems, Framingham, MA, USA).

### Selection of single nucleotide polymorphism markers and genotyping

For the (Col×(Col×Ler)) and ((Col×Ler)×Col) populations, a set of 384 SNPs markers (Table S1) were chosen from Monsanto database and the Salk Institute data-base on the basis of an even physical spacing along the chromosomes. Markers were validated according to Illumina with their Assay Design Tool http://www.illumina.com/. Support and genotyping was carried out at the Plateforme Génomique de Toulouse using BeadXpress technology http://www.illumina.com. BeadXpress raw data were processed using Illumina's BeadStudio Genotyping Module V3.2 software and report files produced containing normalized intensity data and SNP genotypes were transferred to a Microsoft file for analysis. Genotypes were checked using a genotyping cluster file automatically generated by BeadStudio. Nine additional markers (Table S1) were genotyped at the CNG using TaqMan probes (assay-by-design Service Overview, Applied Biosystems) according to the manufacturer's recommendations and end point fluorescence was detected using an ABI7900HT reader (Applied Biosystems, Framingham, MA, USA). Scatter plots for each SNP locus were obtained using the SDS Software Workspace (Applied Biosystems). Fluorescence data were transferred to a Microsoft Excel file for analysis.

Markers and plants with too many undetermined genotypes were removed from the final dataset. The resulting populations comprised on average 1,505 and 1,507 plants with genotype data from 380 and 386 markers for the male and female populations, respectively (380 markers in common). We used PCR and DNA sequencing to verify 222 and 163 singletons in the male and female populations, respectively.

### Analysis of segregation distortion

For a given population, we call *N_C_* (respectively *N_L_*) the number of plants with the Col (respectively Ler) allele at a particular locus. To see the statistical significance of the segregation distortion at that locus, we tested whether the hypothesis of no distortion (a fraction 0.5 for each allele) resulted in a *p*-value smaller than 1%. This defined a region outside of an interval centered on the value 0.5; the half-width of this interval is 2.33 *s* where *s* is the standard error satisfying *s* ^2^ = 1/(4 (*N_C_*+*N_L_*)). The associated bands for all chromosomes (cf. Figure 1) were slightly irregular; this is because the number of valid data varied at each locus.

### Chromosome-wide genetic lengths

Two methods can be used to determine the genetic length (*L_G_*) of a chromosome: (1) the lengths of all the intervals are added, using Haldane's formula to go from recombination rate to genetic distance; (2) the number of COs for each plant is averaged, assuming that one never has more than one CO at a time in the same interval. Both approaches are excellent approximations given the small interval sizes in this study. These two methods are in fact very similar, but when data are missing, the second method is more precise as it can detect recombination events that are missed by the first approach because it uses more than two loci at a time. Given the number of COs for each plant, extracting the 95% confidence interval on *L_G_* is straightforward; it is 1.96 times the standard error.

A slight generalization is necessary for Table 1 in which we display confidence intervals for the fractions *f* = *L_G_*(male)/*L_G_*(female). Noting that one is in the limit where both the numerator and denominator are well estimated (each has a small relative variance),

we can use the approximation whereby the relative variance of the ratio is replaced by the sum of the relative variances:


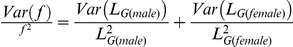
. Furthermore, in this same limit, *f* has a Gaussian distribution so from the variance of *f* we extract in the usual way the desired 95% confidence interval.

To test the hypothesis that male and female genetic lengths were the same (Table 2), we applied the *t*-test using the "t.test" of the software package R. We did this for whole chromosomes and also for chromosomal regions obtained by removing telomeric parts.

### Gamma model measurements of interference strength

To estimate the intensity of crossover interference, we have fitted the gamma interference model to our crossover data for each chromosome in male and female meiosis separately, following the procedure described by McPeek and Speed [8], and Broman and Weber, [74]. Such models parametrize the distribution of distances between successive crossovers. They may be fitted to experimental data by using a classical maximum-likelihood approach, taking advantage of the fact that the gamma model in particular makes it possible to compute the likelihood of a set of experimental crossover positions as a function of the parameter *nu*. The estimate of this parameter is thus a measurement of the interference strength. It can also be thought of as a generalization allowing a continuous interference parameter satisfying *nu*  = *m*+1, where *m* is an integer associated with counting discrete events in the counting model [7]. To fit the model to our data, we used what is referred to as “thinning” [8], [74]: the gamma model describes the crossovers at the level of the bivalents, so to get a model for crossovers at the gametic level, it is necessary to thin, *i.e*, remove with probability 0.5 crossovers on the bivalent. Then using such thinning makes it possible to fit the gamma model to marker segregation data, and we used this procedure here.

### Comparing recombination rates between different intervals

The recombination rate between two adjacent markers is estimated from the number of recombinants, using only plants that have no missing data at those two markers. If *N_r_* (respectively *N*) is the number of recombinant (respectively all) plants, the recombination rate r is estimated as *N_r_*/*N*. The corresponding 95% confidence interval is given by 1.96 *s* where *s* is the standard error satisfying *s* ^2^ = *r* (1−*r*)/*N*. The recombination rate *per base pair* (and the associated confidence interval) is obtained by dividing by the number of base pairs. Finally, the mean recombination rate per base pair on a chromosome arm is calculated using a weight for each interval, which is simply its length in base pairs. Intervals belonging to heterochromatic regions (see below) are excluded from the calculation. Intervals are defined as hot or cold (cf. Figure 4) if their 95% confidence intervals do not contain the mean calculated for that arm. Male and female recombination rates are considered as significantly different when the statistical test of equality gives a *p*-value of less than 5% (Benjamini correction included) [75]. The same method as above was used to compare male and female recombination rate from [27] to those obtained in this study (Benjamini correction included).

We use epigenetic features to infer the heterochromatic regions. We first take the levels of H3K4me3 and H3K27me3 modifications as measured in plantlets for each gene, one at a time (data provided by [76]). Both are markers of euchromatin, so we first test for the presence of either of these. Then 2 kb sized windows are used to obtain average levels of presence. Finally, we consider an interval to be heterochromatic if the average in that interval is below the threshold 0.2. As expected, in all chromosomes, the centromeric region is then labeled as heterochromatic as well as the pericentromeric regions. Furthermore, following this procedure, chromosome 4 has a large heterochromatic region on its short arm, again in agreement with inferences in previous works.

### Association of CO rates with genomic features

To test for possible associations between recombination rates (per base pair) and genomic features, one must first remove the centromeric regions (which have low recombination and have unusual genomic content), otherwise they would dominate the analysis. We thus exclude all the heterochromatic intervals (defined as explained previously). Then for each remaining interval, we use the TAIR9 data files to determine the following contents, measured per base pair: GC, coding GC, GC1, GC2, GC3, CpG, and gene density. The potential linear association between these quantities is examined via the R^2^ of the fit and the *p*-value for the hypothesis of no association using the implementation provided by "lm" in the R software package.

### Correcting the genetic length from effects of segregation distortion

Consider the segregation distortion along chromosome 1 for male meiosis, the profile indicates strong distortion around two loci with a relatively smooth behavior between the two, making it plausible that only those two loci are under selection. Clearly, such a segregation distortion can bias our estimate of genetic lengths; we present here a simple model for correcting for such a bias.

As a first simpler case, suppose that only one locus is under selection. We parameterize the selection process by having the meiosis happen normally (no segregation distortion) but follow it by keeping only a fraction *s* of the gametes that carry the less favored allele at the locus under selection. The gametes carrying the favored allele are all kept. Say we want to examine the recombination rate between two markers; let *r* be this rate before selection. The four possible genotypes of a gamete at these two markers are AB, Ab, aB, ab and before selection their frequencies are (1−*r*)/2, *r*/2, *r*/2 and (1−*r*)/2. Among both the recombinant and non-recombinant genotypes, exactly half of the gametes carry the favored allele and half carry the unfavored allele. The selection process changes the number of recombinants by a factor (1+*s*)/2, but the same is true of the non-recombinants. Thus the naive estimation of the recombination rate, given by the fraction of *observed* recombinants, is an unbiased estimator for the true recombination rate *r*.

Now to deal with the case where two loci L_1_ and L_2_ are under selection, we generalize the previous parametrization by having two selection coefficients, *s_1_* and *s_2_.* In our context, the less frequent allele is Col for the locus L_1_ and Ler for L_2_. If a gamete has both favored alleles, it is kept; if it has one unfavored allele, selection keeps it with probability *s_1_* or *s_2_* depending on the locus with that allele; and finally if the gamete carries both unfavored alleles, selection keeps it with probability *s_1_s_2_* (no epistasis). In contrast to the single locus case, the selection here *does* change the ratio of recombinant and non recombinant gametes. It is thus necessary to use a more sophisticated estimate of the recombination rate between two markers than the naive estimate (the fraction of measured recombinants). We do so as follows. For each interval delimited by adjacent markers M_i_ and M_i+1_, we enumerate all possible genotypes for those markers and for the two loci under selection. If these markers are distinct from the two loci – which we assume here for simplicity of presentation –, there are 16 possible genotypes. When we order L_1_, L_2_, M_i_, and M_i+1_ along the map, we define three consecutive intervals. Within the standard Haldane model of CO formation, the frequencies of the 16 genotypes are simply determined by the three recombination rates r_12_, r_23_, r_34_ of these intervals. To go from these frequencies to the ones after gametic selection is a simple affair and of course involves selection coefficients. Our computation is decomposed into the following steps. Assuming *s_1_* and *s_2_* given, we first use the 16 measured frequencies to fit the three unknown parameters *r*_12_, *r*_23_, *r*_34_. Minimizing the weighted chi squared between the 16 observed and theoretical frequencies performs this fit. Then, we add the chi squared for all the intervals, defining a total chi squared for the pair (*s_1_*, *s_2_*). This total chi squared is then minimized, leading to the inferred values (s*_1_, s*_2_) of the selection coefficients. Finally, using (s*_1_, s*_2_), the recombination rates for all the intervals (M_i_, M_i+1_) are recomputed and from that we extract the corrected total genetic length. In practice, when the two loci under selection are far away as in the case of chromosome 1, the correction vanishes outside of (L1, L2) because effectively one then has only one relevant marker under selection. We thus used the procedure just described only for those intervals (Mi, Mi+1) between (L1, L2).

This approach to correct for segregation bias in the genetic length *L_G_* was only necessary for chromosome 1 (male meiosis), slightly increasing the naïve estimate of that chromosome's genetic length (see Table 1). As a consequence, the difference between male and female on chromosome 1 is slightly enhanced by the correction, and so omitting this correction in such tests is conservative. In particular for Table 2, where the test is performed on whole and truncated chromosomes, we see that even without this correction, the male/female ratio is significantly statistically different from one.

### Comparison of chromosome-wide genetic length to the model proposed by Li and Freudenberg

This model [55] stipulates that the genetic length rises linearly with physical length but has an offset associated with the obligatory CO. This corresponds to the relationship 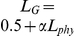 where α is a proportionality constant.

We have fitted this formula for the 5 chromosomes of Arabidopsis, treating separately the M and F cases (there is thus one value of α for M and one for F). In the case of chromosome 1 M, the genetic length has been corrected to take into account the segregation distortion (see previous explanations). The fits have been implemented by linear regression minimizing the weighted chi-sqared 
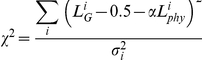
where 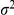 is the variance of the estimator of the genetic length of chromosome *i*. Explicitly, 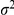 is calculated as the variance of the number of COs on chromosome *i* divided by the number of plants used in this experiment. The test of the model of Li and Freudenberg is obtained by using the value of 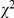 after the fit taking into account the number of degrees of freedom.

## Supporting Information

Figure S1 Distribution of along chromosome arms, and correlation with recombination rate. Global GC: Proportion of G or C nucleotides in the whole interval. CpG: ratio between the number of CpG or GpC dinucleotides over the length of the sequence in the interval. Genes: proportion of bases which belong to a gene. TE: proportion of bases which belong to a transposable element. For each of the four genomic features, the figure shows (1) the distributions of the genomic feature along chromosome arms and (2) the correlation between the genomic feature and recombination rate in male and female meiosis for the entire chromosome (solid lines and “plus” symbols) and when 30% and 50% of the physical length were removed from both extremities of the chromosome (dashed lines and “circle” and “plus” superimposed symbols). Each point corresponds to one interval between markers.
(PDF)

Table S1 List of SNPs used for genotyping. * Genotyping performed with Taqman technology. (1) SNPs genotyped only in female population.
(PDF)

Table S2 Characterization of the intervals along the 5 chromosomes.
(XLS)

Table S3 Correlation between CO rates and chromosome features. (a) positive (+) or negative (-) correlation.
(PDF)

Table S4 Observed Number of chromosomes having 0, 1, 2 or more COs.
(PDF)

Table S5 Effect of the truncations on the distribution of chromosomes with 1 or 2 or more COs. (a) Based on the genetic map length. Observed loss of chromosomes with one CO. (c) Observed loss with chromosomes with 2 or more COs.
(PDF)
