# Supplementary material for: Genome-Wide Crossover Distribution in Arabidopsis thaliana Meiosis Reveals Sex-Specific Patterns along Chromosomes
Source: PLoS Genet. 2011 Nov 3;7(11):e1002354. doi: 10.1371/journal.pgen.1002354 (PMC3207851; doi:10.1371/journal.pgen.1002354)
Supplement: Figure S1 — Distribution of along chromosome arms, and correlation with recombination rate. Global GC: Proportion of G or C nucleotides in the whole interval. CpG: ratio between the number of CpG or GpC dinucleotides over the length of the sequence in the interval. Genes: proportion of bases which belong to a gene. TE: proportion of bases which belong to a transposable element. For each of the four genomic features, the figure shows (1) the distributions of the genomic feature along chromosome arms and (2) the correlation between the genomic feature and recombination rate in male and female meiosis for the entire chromosome (solid lines and “plus” symbols) and when 30% and 50% of the physical length were removed from both extremities of the chromosome (dashed lines and “circle” and “plus” superimposed symbols). Each point corresponds to one interval between markers. (PDF) [file pgen.1002354.s001.pdf]

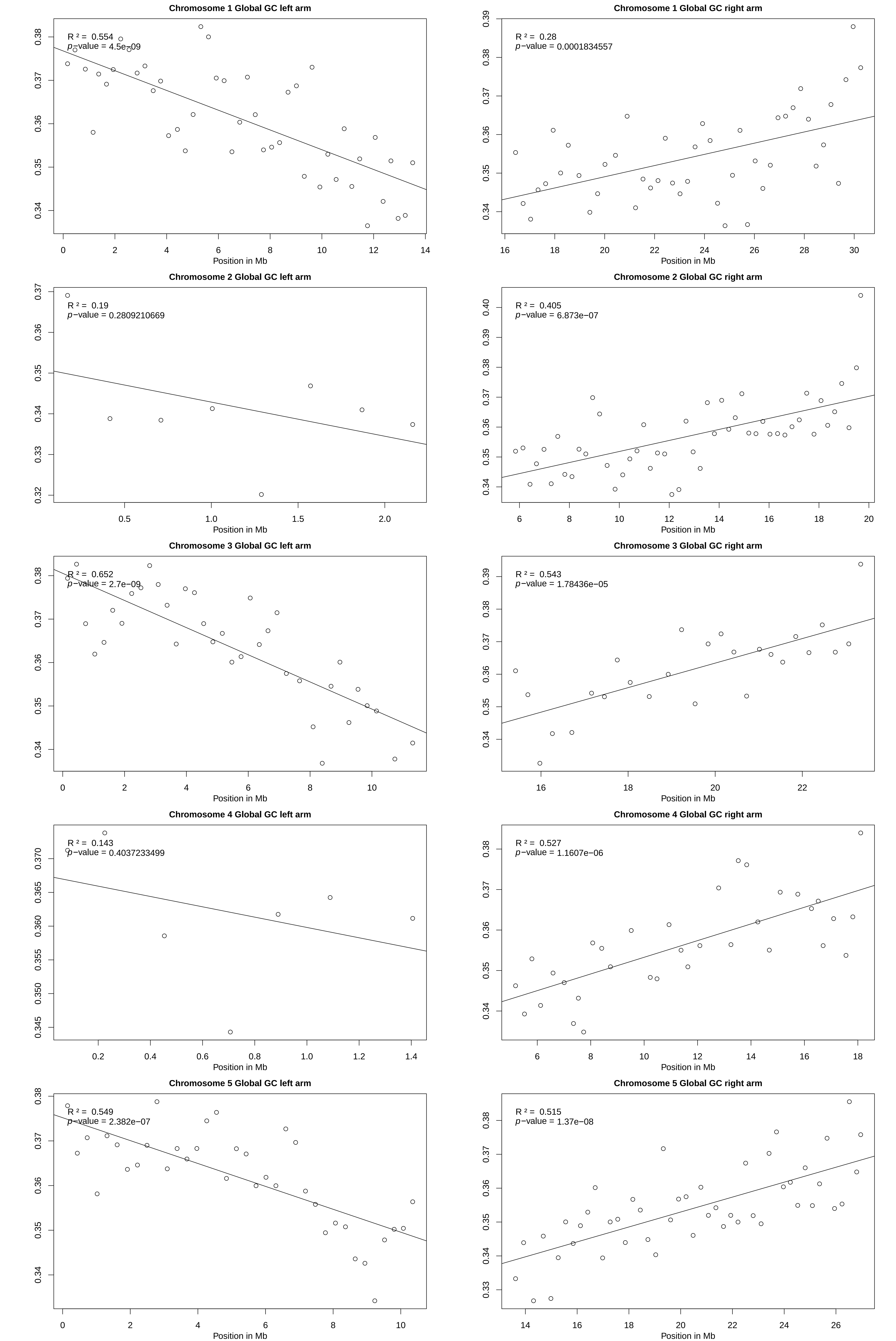
Chromosome 1 Global GC left arm
R ² = 0.554
p−value = 4.5e−09
Position in Mb
Chromosome 1 Global GC right arm
R ² = 0.28
p−value = 0.0001834557
Position in Mb
Chromosome 2 Global GC left arm
R ² = 0.19
p−value = 0.2809210669
Position in Mb
Chromosome 2 Global GC right arm
R ² = 0.405
p−value = 6.873e−07
Position in Mb
Chromosome 3 Global GC left arm
R ² = 0.652
p−value = 2.7e−09
Position in Mb
Chromosome 3 Global GC right arm
R ² = 0.543
p−value = 1.78436e−05
Position in Mb
Chromosome 4 Global GC left arm
R ² = 0.143
p−value = 0.4037233499
Position in Mb
Chromosome 4 Global GC right arm
R ² = 0.527
p−value = 1.1607e−06
Position in Mb
Chromosome 5 Global GC left arm
R ² = 0.549
p−value = 2.382e−07
Position in Mb
Chromosome 5 Global GC right arm
R ² = 0.515
p−value = 1.37e−08
Position in Mb

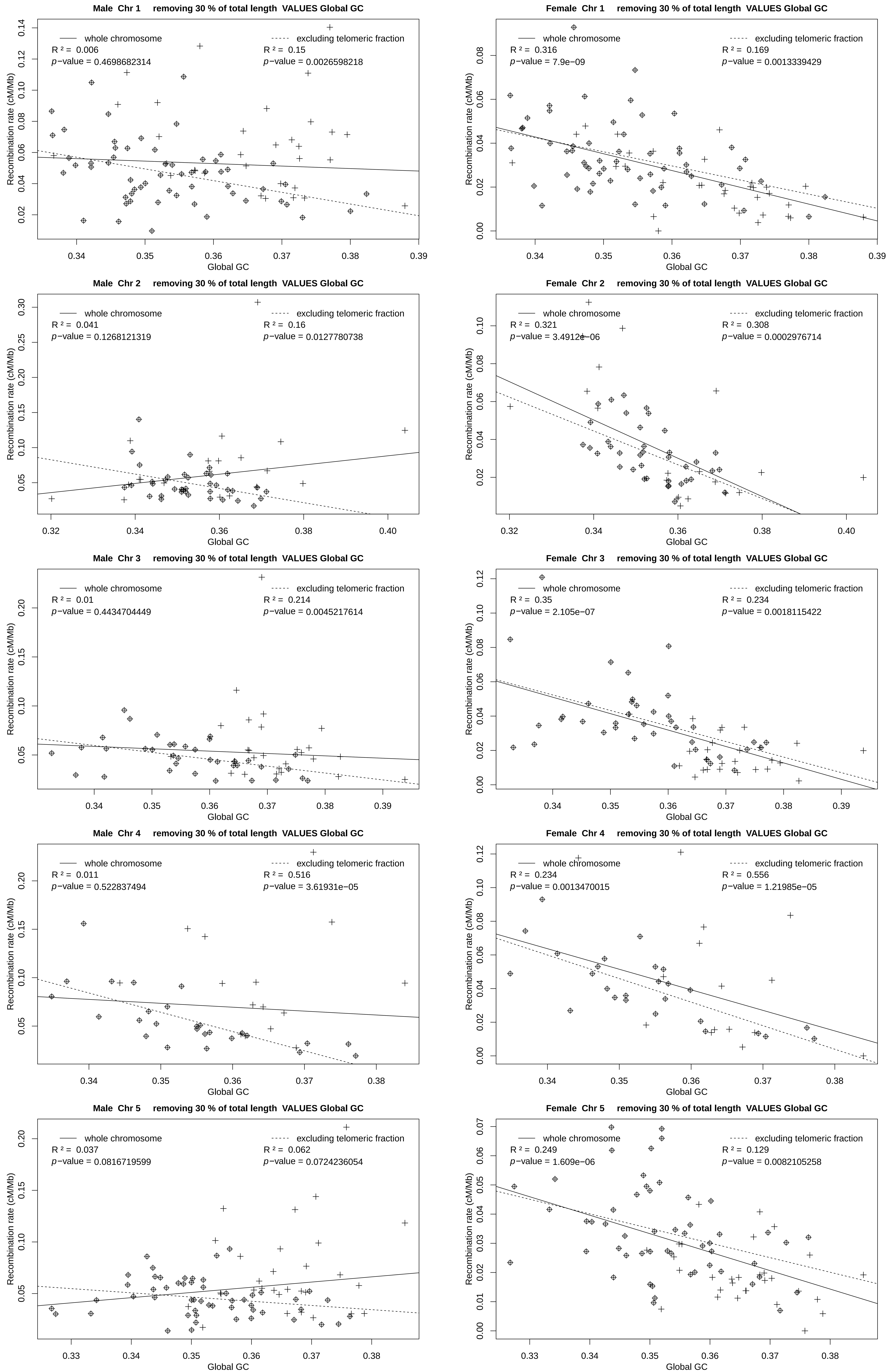

Male Chr 1 removing 30 % of total length VALUES Global GC
whole chromosome
R ² = 0.006
p−value = 0.4698682314
excluding telomeric fraction
R ² = 0.15
p−value = 0.0026598218
Female Chr 1 removing 30 % of total length VALUES Global GC
whole chromosome
R ² = 0.316
p−value = 7.9e−09
excluding telomeric fraction
R ² = 0.169
p−value = 0.0013339429
Male Chr 2 removing 30 % of total length VALUES Global GC
whole chromosome
R ² = 0.041
p−value = 0.1268121319
excluding telomeric fraction
R ² = 0.16
p−value = 0.0127780738
Female Chr 2 removing 30 % of total length VALUES Global GC
whole chromosome
R ² = 0.321
p−value = 3.4912e−06
excluding telomeric fraction
R ² = 0.308
p−value = 0.0002976714
Male Chr 3 removing 30 % of total length VALUES Global GC
whole chromosome
R ² = 0.01
p−value = 0.4434704449
excluding telomeric fraction
R ² = 0.214
p−value = 0.0045217614
Female Chr 3 removing 30 % of total length VALUES Global GC
whole chromosome
R ² = 0.35
p−value = 2.105e−07
excluding telomeric fraction
R ² = 0.234
p−value = 0.0018115422
Male Chr 4 removing 30 % of total length VALUES Global GC
whole chromosome
R ² = 0.011
p−value = 0.522837494
excluding telomeric fraction
R ² = 0.516
p−value = 3.61931e−05
Female Chr 4 removing 30 % of total length VALUES Global GC
whole chromosome
R ² = 0.234
p−value = 0.0013470015
excluding telomeric fraction
R ² = 0.556
p−value = 1.21985e−05
Male Chr 5 removing 30 % of total length VALUES Global GC
whole chromosome
R ² = 0.037
p−value = 0.0816719599
excluding telomeric fraction
R ² = 0.062
p−value = 0.0724236054
Female Chr 5 removing 30 % of total length VALUES Global GC
whole chromosome
R ² = 0.249
p−value = 1.609e−06
excluding telomeric fraction
R ² = 0.129
p−value = 0.0082105258
Recombination rate (cM/Mb)
Global GC

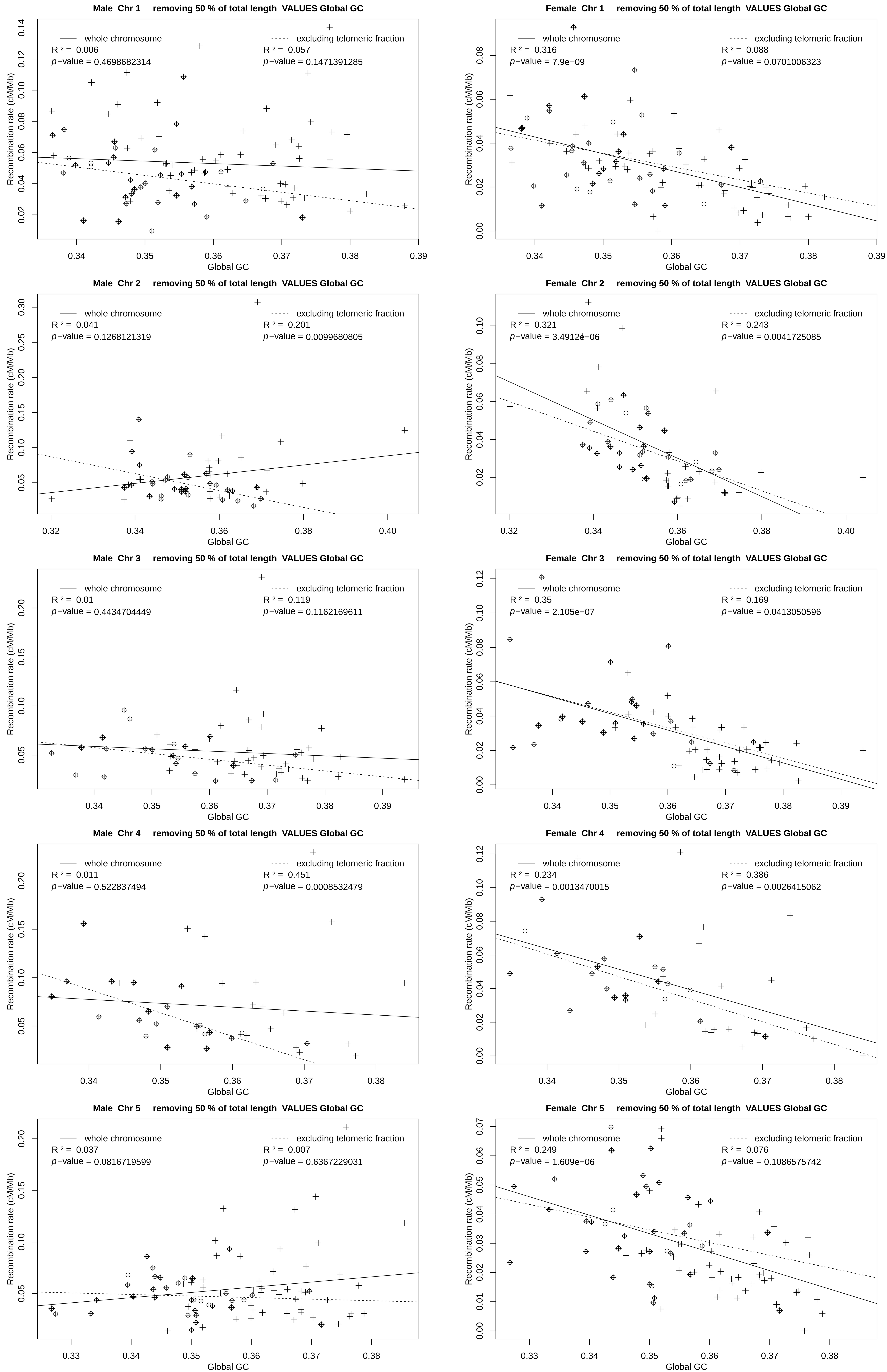
Male Chr 1 removing 50 % of total length VALUES Global GC
whole chromosome
R ² = 0.006
p−value = 0.4698682314
excluding telomeric fraction
R ² = 0.057
p−value = 0.1471391285
Recombination rate (cM/Mb)
Global GC
Female Chr 1 removing 50 % of total length VALUES Global GC
whole chromosome
R ² = 0.316
p−value = 7.9e−09
excluding telomeric fraction
R ² = 0.088
p−value = 0.0701006323
Recombination rate (cM/Mb)
Global GC
Male Chr 2 removing 50 % of total length VALUES Global GC
whole chromosome
R ² = 0.041
p−value = 0.1268121319
excluding telomeric fraction
R ² = 0.201
p−value = 0.0099680805
Recombination rate (cM/Mb)
Global GC
Female Chr 2 removing 50 % of total length VALUES Global GC
whole chromosome
R ² = 0.321
p−value = 3.4912e−06
excluding telomeric fraction
R ² = 0.243
p−value = 0.0041725085
Recombination rate (cM/Mb)
Global GC
Male Chr 3 removing 50 % of total length VALUES Global GC
whole chromosome
R ² = 0.01
p−value = 0.4434704449
excluding telomeric fraction
R ² = 0.119
p−value = 0.1162169611
Recombination rate (cM/Mb)
Global GC
Female Chr 3 removing 50 % of total length VALUES Global GC
whole chromosome
R ² = 0.35
p−value = 2.105e−07
excluding telomeric fraction
R ² = 0.169
p−value = 0.0413050596
Recombination rate (cM/Mb)
Global GC
Male Chr 4 removing 50 % of total length VALUES Global GC
whole chromosome
R ² = 0.011
p−value = 0.522837494
excluding telomeric fraction
R ² = 0.451
p−value = 0.0008532479
Recombination rate (cM/Mb)
Global GC
Female Chr 4 removing 50 % of total length VALUES Global GC
whole chromosome
R ² = 0.234
p−value = 0.0013470015
excluding telomeric fraction
R ² = 0.386
p−value = 0.0026415062
Recombination rate (cM/Mb)
Global GC
Male Chr 5 removing 50 % of total length VALUES Global GC
whole chromosome
R ² = 0.037
p−value = 0.0816719599
excluding telomeric fraction
R ² = 0.007
p−value = 0.6367229031
Recombination rate (cM/Mb)
Global GC
Female Chr 5 removing 50 % of total length VALUES Global GC
whole chromosome
R ² = 0.249
p−value = 1.609e−06
excluding telomeric fraction
R ² = 0.076
p−value = 0.1086575742
Recombination rate (cM/Mb)
Global GC

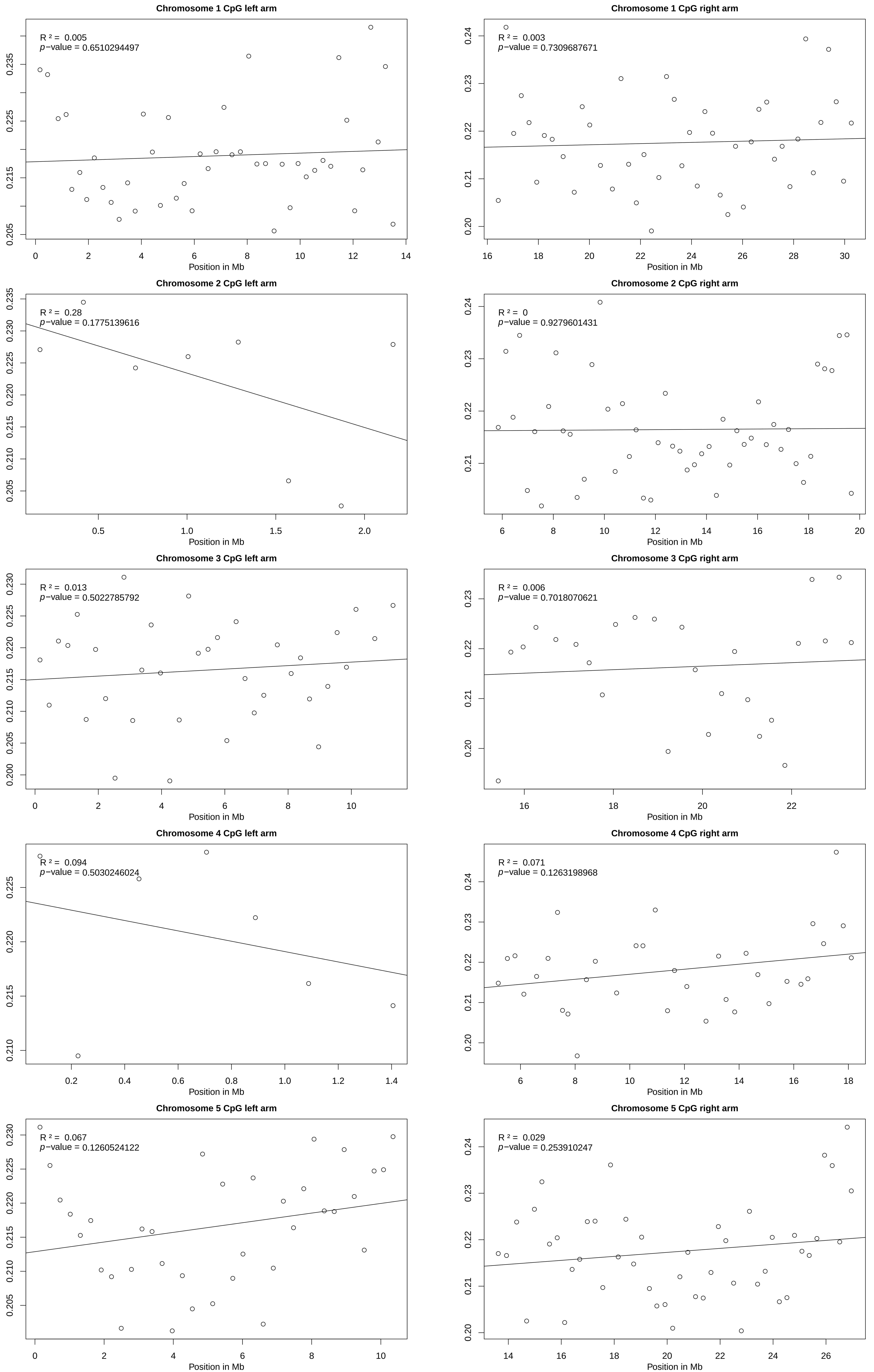
Chromosome 1 CpG left arm
R ² = 0.005
p−value = 0.6510294497
Position in Mb
Chromosome 1 CpG right arm
R ² = 0.003
p−value = 0.7309687671
Position in Mb
Chromosome 2 CpG left arm
R ² = 0.28
p−value = 0.1775139616
Position in Mb
Chromosome 2 CpG right arm
R ² = 0
p−value = 0.9279601431
Position in Mb
Chromosome 3 CpG left arm
R ² = 0.013
p−value = 0.5022785792
Position in Mb
Chromosome 3 CpG right arm
R ² = 0.006
p−value = 0.7018070621
Position in Mb
Chromosome 4 CpG left arm
R ² = 0.094
p−value = 0.5030246024
Position in Mb
Chromosome 4 CpG right arm
R ² = 0.071
p−value = 0.1263198968
Position in Mb
Chromosome 5 CpG left arm
R ² = 0.067
p−value = 0.1260524122
Position in Mb
Chromosome 5 CpG right arm
R ² = 0.029
p−value = 0.253910247
Position in Mb

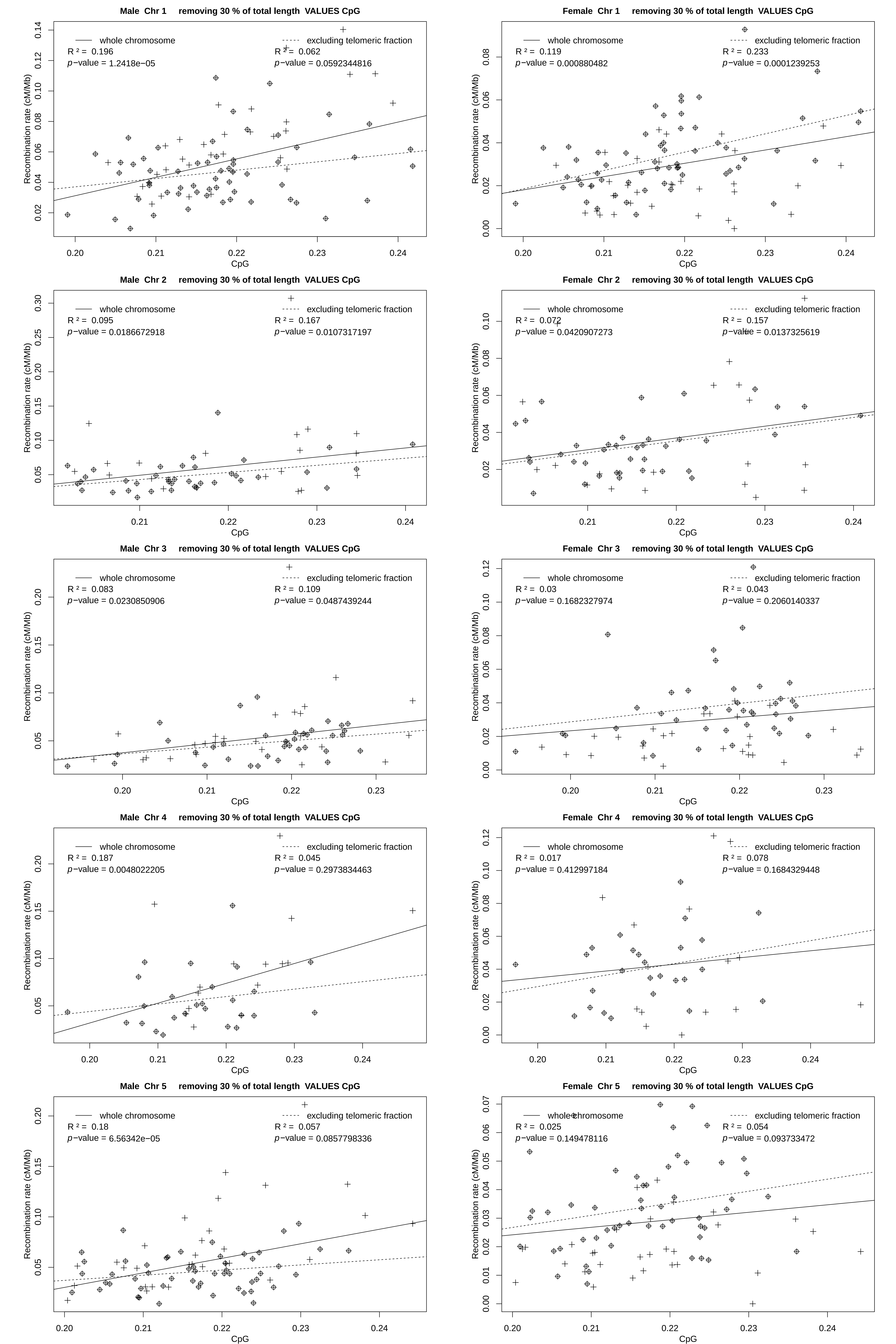
Male Chr 1 removing 30 % of total length VALUES CpG
whole chromosome
R ² = 0.196
p-value = 1.2418e−05
excluding telomeric fraction
R ² = 0.062
p-value = 0.0592344816
Recombination rate (cM/Mb)
CpG
Female Chr 1 removing 30 % of total length VALUES CpG
whole chromosome
R ² = 0.119
p-value = 0.000880482
excluding telomeric fraction
R ² = 0.233
p-value = 0.0001239253
Male Chr 2 removing 30 % of total length VALUES CpG
whole chromosome
R ² = 0.095
p-value = 0.0186672918
excluding telomeric fraction
R ² = 0.167
p-value = 0.0107317197
Female Chr 2 removing 30 % of total length VALUES CpG
whole chromosome
R ² = 0.072
p-value = 0.0420907273
excluding telomeric fraction
R ² = 0.157
p-value = 0.0137325619
Male Chr 3 removing 30 % of total length VALUES CpG
whole chromosome
R ² = 0.083
p-value = 0.0230850906
excluding telomeric fraction
R ² = 0.109
p-value = 0.0487439244
Female Chr 3 removing 30 % of total length VALUES CpG
whole chromosome
R ² = 0.03
p-value = 0.1682327974
excluding telomeric fraction
R ² = 0.043
p-value = 0.2060140337
Male Chr 4 removing 30 % of total length VALUES CpG
whole chromosome
R ² = 0.187
p-value = 0.0048022205
excluding telomeric fraction
R ² = 0.045
p-value = 0.2973834463
Female Chr 4 removing 30 % of total length VALUES CpG
whole chromosome
R ² = 0.017
p-value = 0.412997184
excluding telomeric fraction
R ² = 0.078
p-value = 0.1684329448
Male Chr 5 removing 30 % of total length VALUES CpG
whole chromosome
R ² = 0.18
p-value = 6.56342e−05
excluding telomeric fraction
R ² = 0.057
p-value = 0.0857798336
Female Chr 5 removing 30 % of total length VALUES CpG
whole chromosome
R ² = 0.025
p-value = 0.149478116
excluding telomeric fraction
R ² = 0.054
p-value = 0.093733472

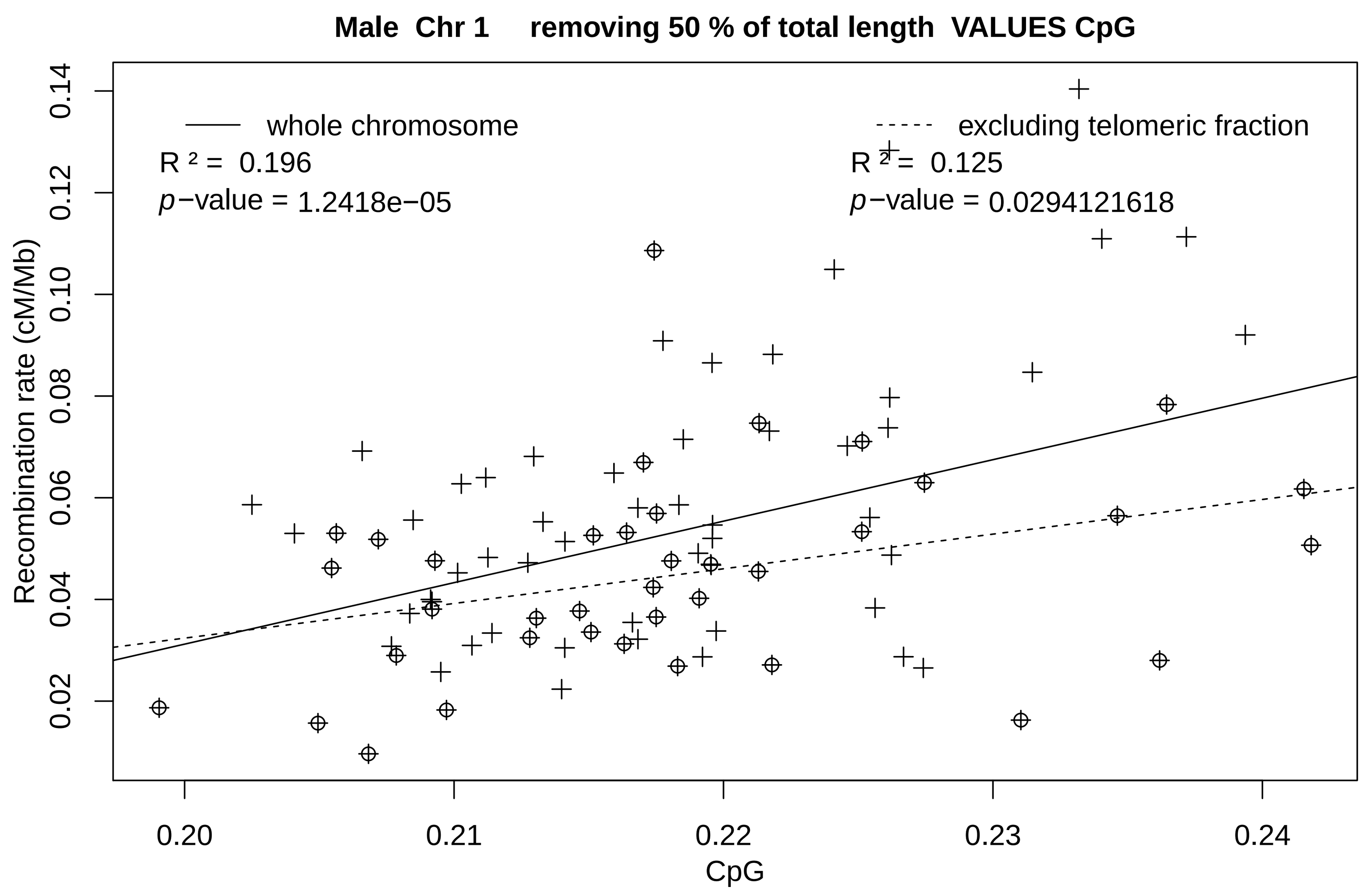
Male Chr 1 removing 50 % of total length VALUES CpG
whole chromosome
R ² = 0.196
p−value = 1.2418e−05
excluding telomeric fraction
R ² = 0.125
p−value = 0.0294121618
Recombination rate (cM/Mb)
CpG

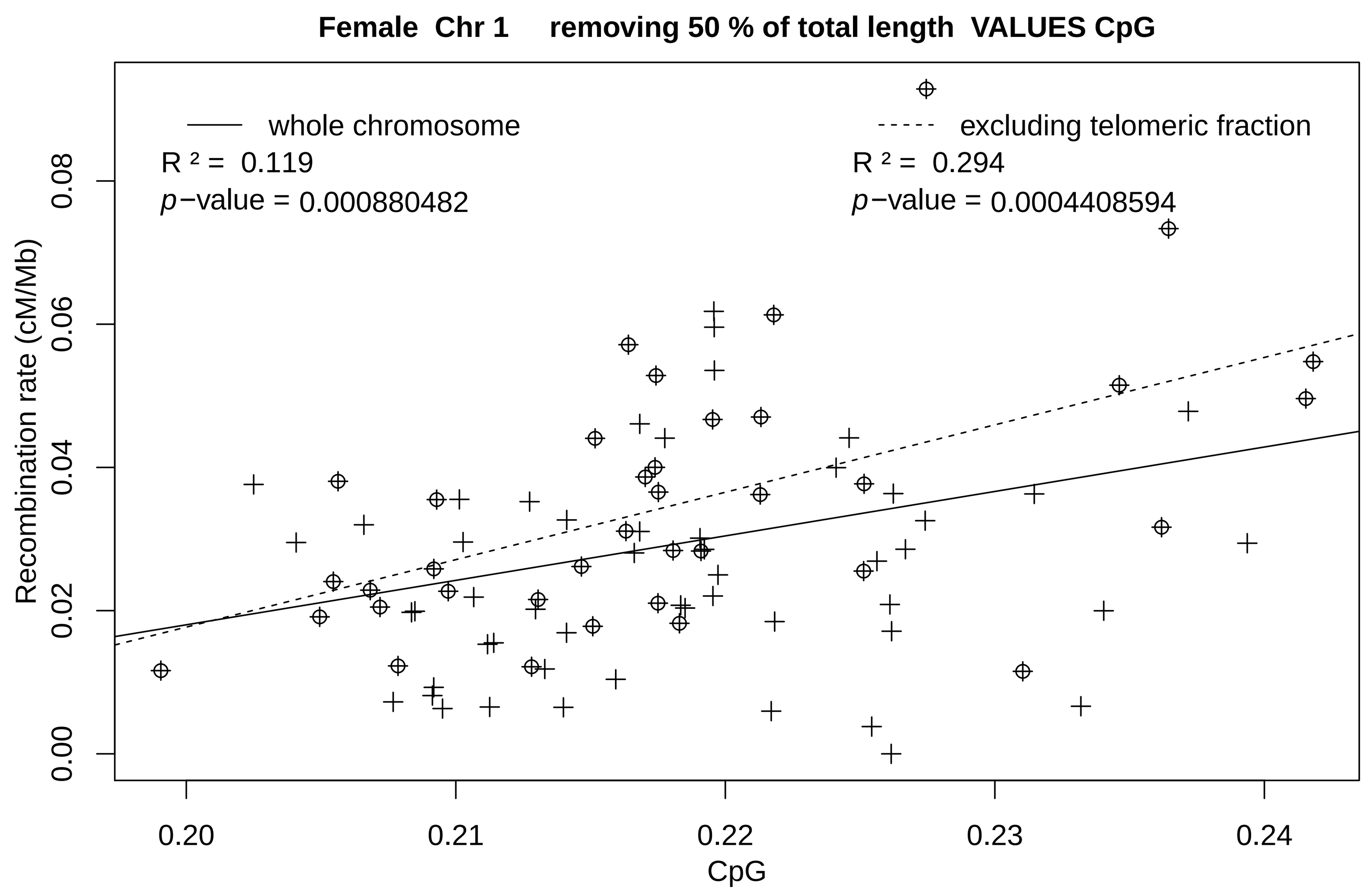
Female Chr 1 removing 50 % of total length VALUES CpG
whole chromosome
R ² = 0.119
p−value = 0.000880482
excluding telomeric fraction
R ² = 0.294
p−value = 0.0004408594
Recombination rate (cM/Mb)
CpG

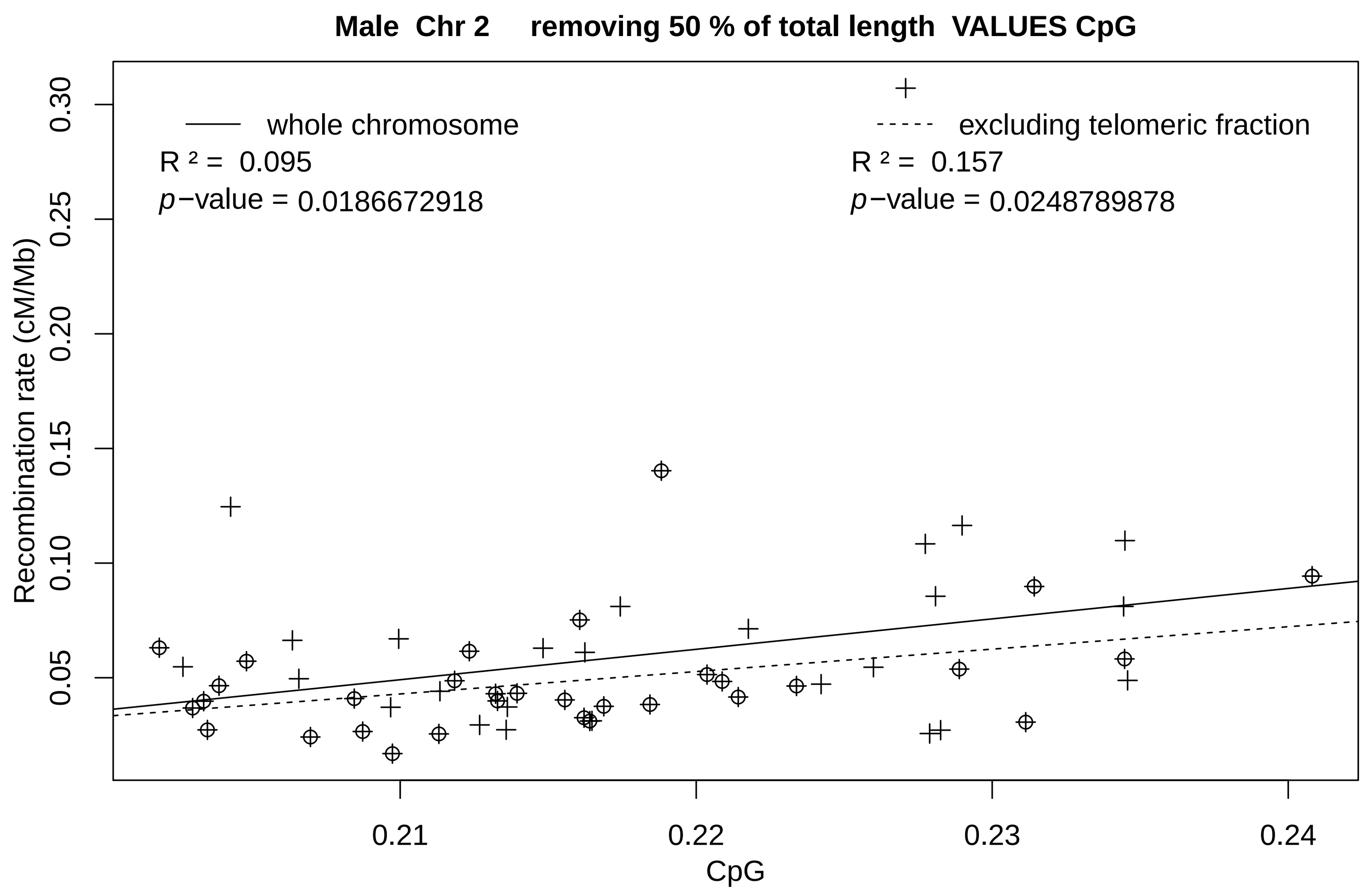
Male Chr 2 removing 50 % of total length VALUES CpG
whole chromosome
R ² = 0.095
p−value = 0.0186672918
excluding telomeric fraction
R ² = 0.157
p−value = 0.0248789878
Recombination rate (cM/Mb)
CpG

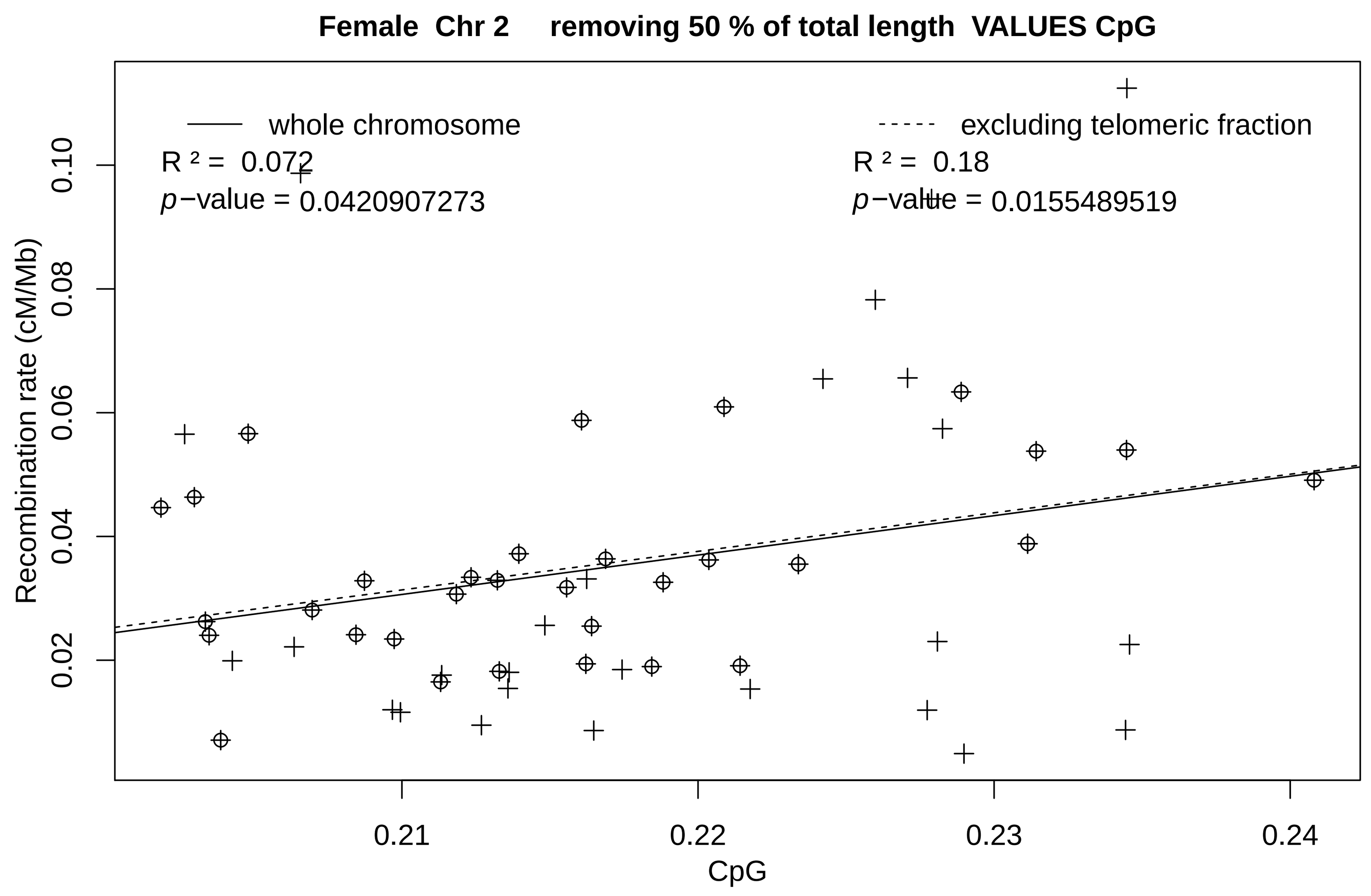
Female Chr 2 removing 50 % of total length VALUES CpG
whole chromosome
R ² = 0.072
p−value = 0.0420907273
excluding telomeric fraction
R ² = 0.18
p−value = 0.0155489519
Recombination rate (cM/Mb)
CpG

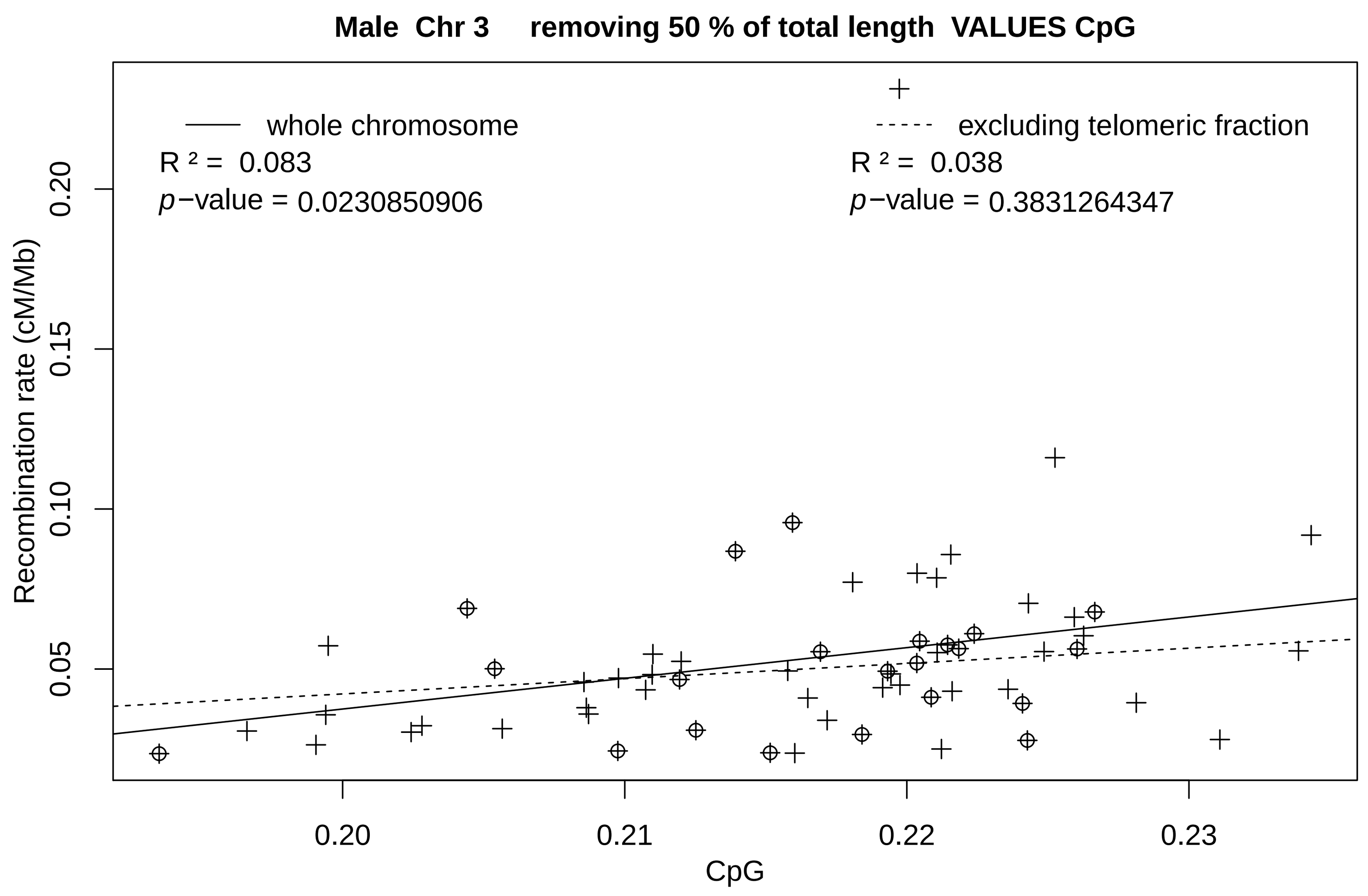
Male Chr 3 removing 50 % of total length VALUES CpG
whole chromosome
R ² = 0.083
p−value = 0.0230850906
excluding telomeric fraction
R ² = 0.038
p−value = 0.3831264347
Recombination rate (cM/Mb)
CpG

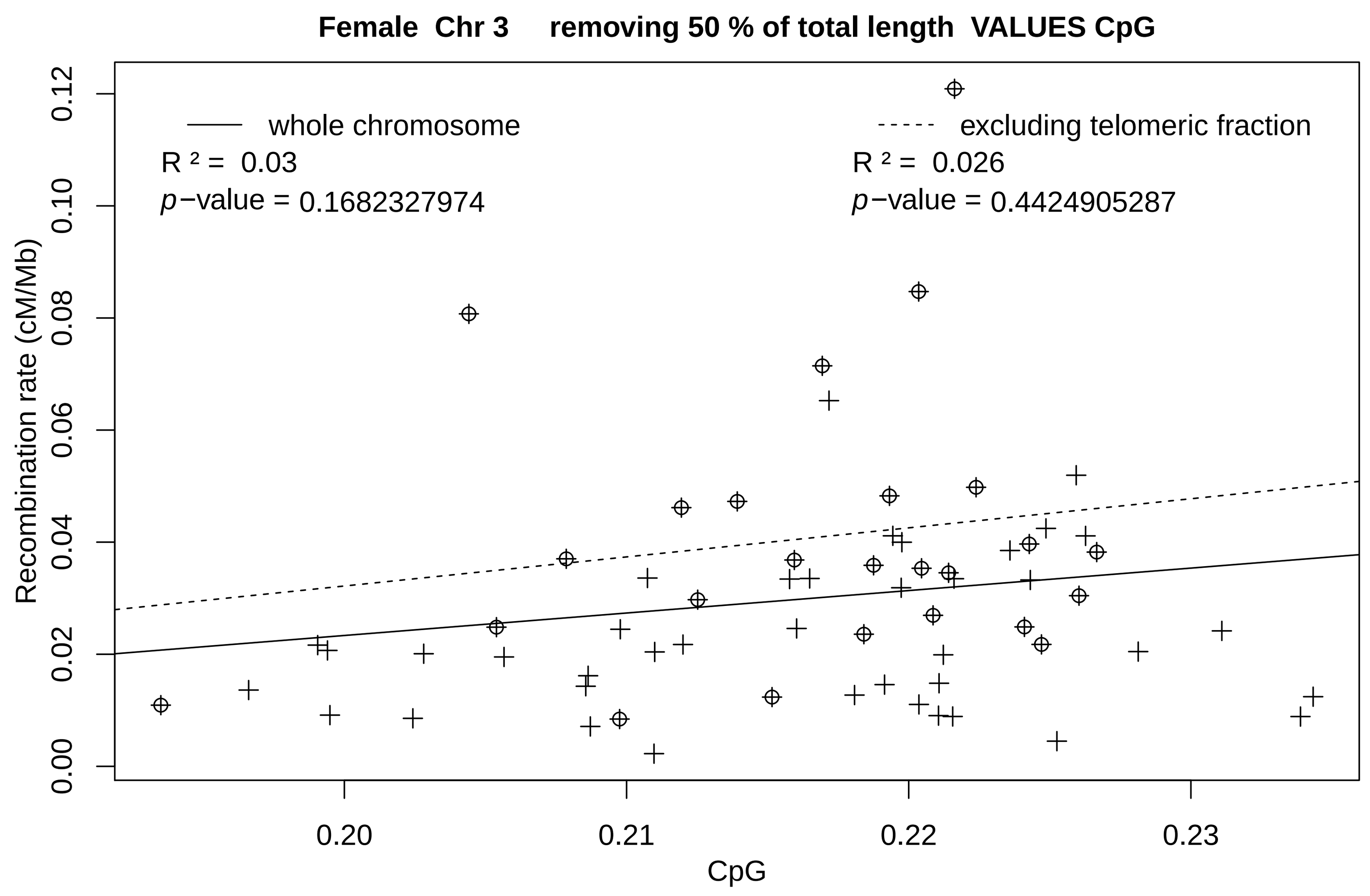
Female Chr 3 removing 50 % of total length VALUES CpG
whole chromosome
R ² = 0.03
p−value = 0.1682327974
excluding telomeric fraction
R ² = 0.026
p−value = 0.4424905287
Recombination rate (cM/Mb)
CpG

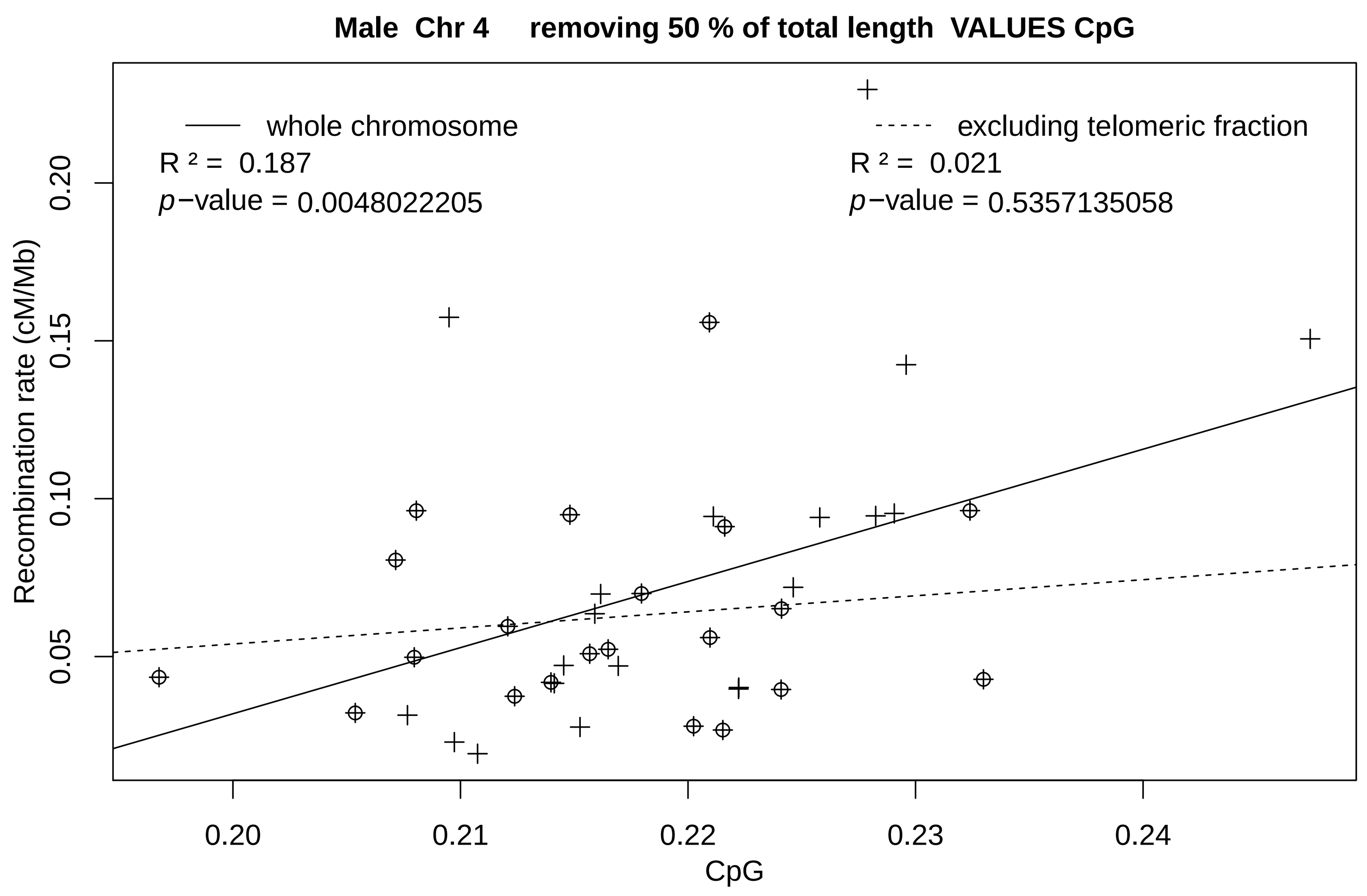
Male Chr 4 removing 50 % of total length VALUES CpG
whole chromosome
R ² = 0.187
p−value = 0.0048022205
excluding telomeric fraction
R ² = 0.021
p−value = 0.5357135058
Recombination rate (cM/Mb)
CpG

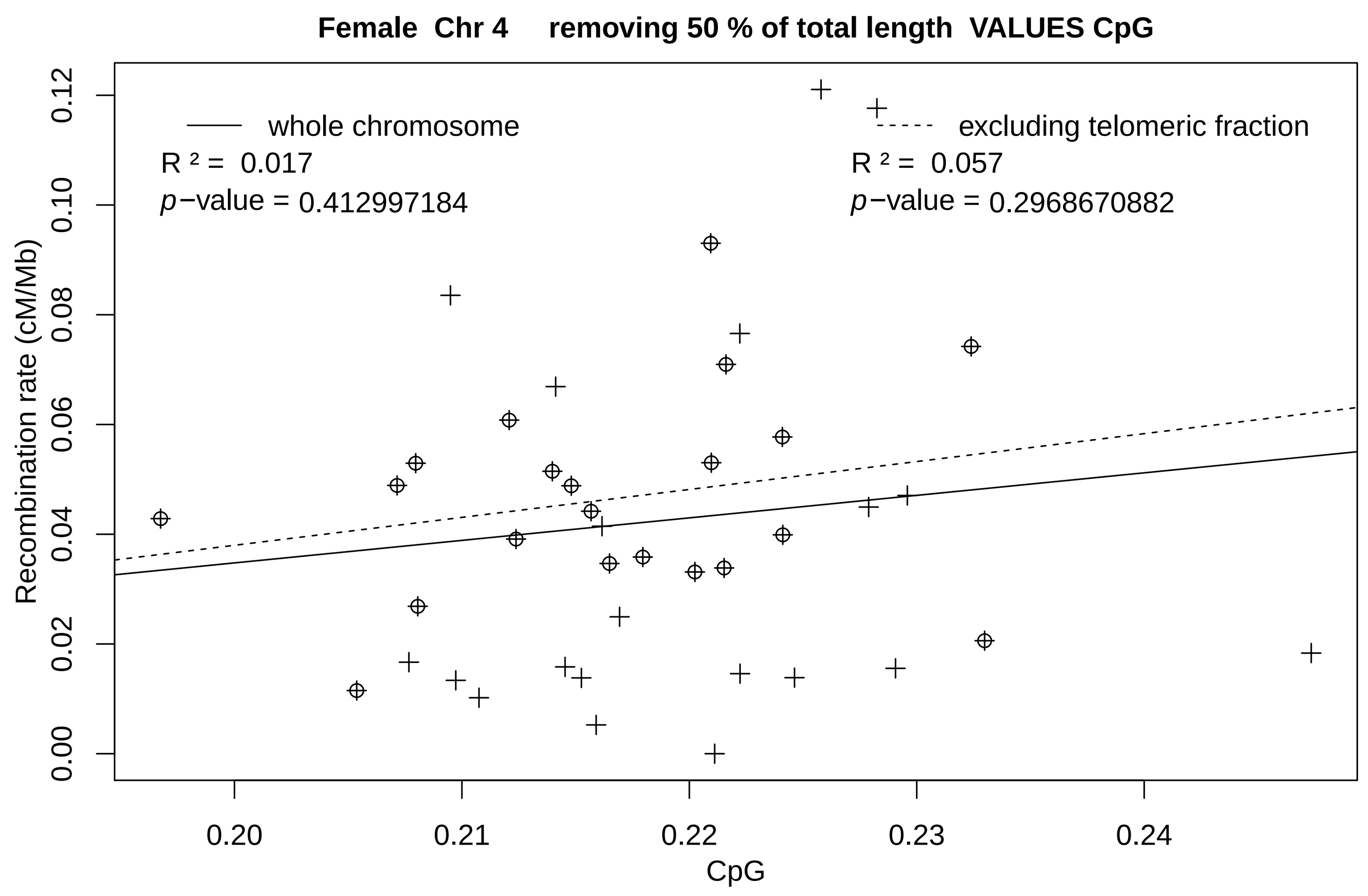
Female Chr 4 removing 50 % of total length VALUES CpG
whole chromosome
R ² = 0.017
p−value = 0.412997184
excluding telomeric fraction
R ² = 0.057
p−value = 0.2968670882
Recombination rate (cM/Mb)
CpG

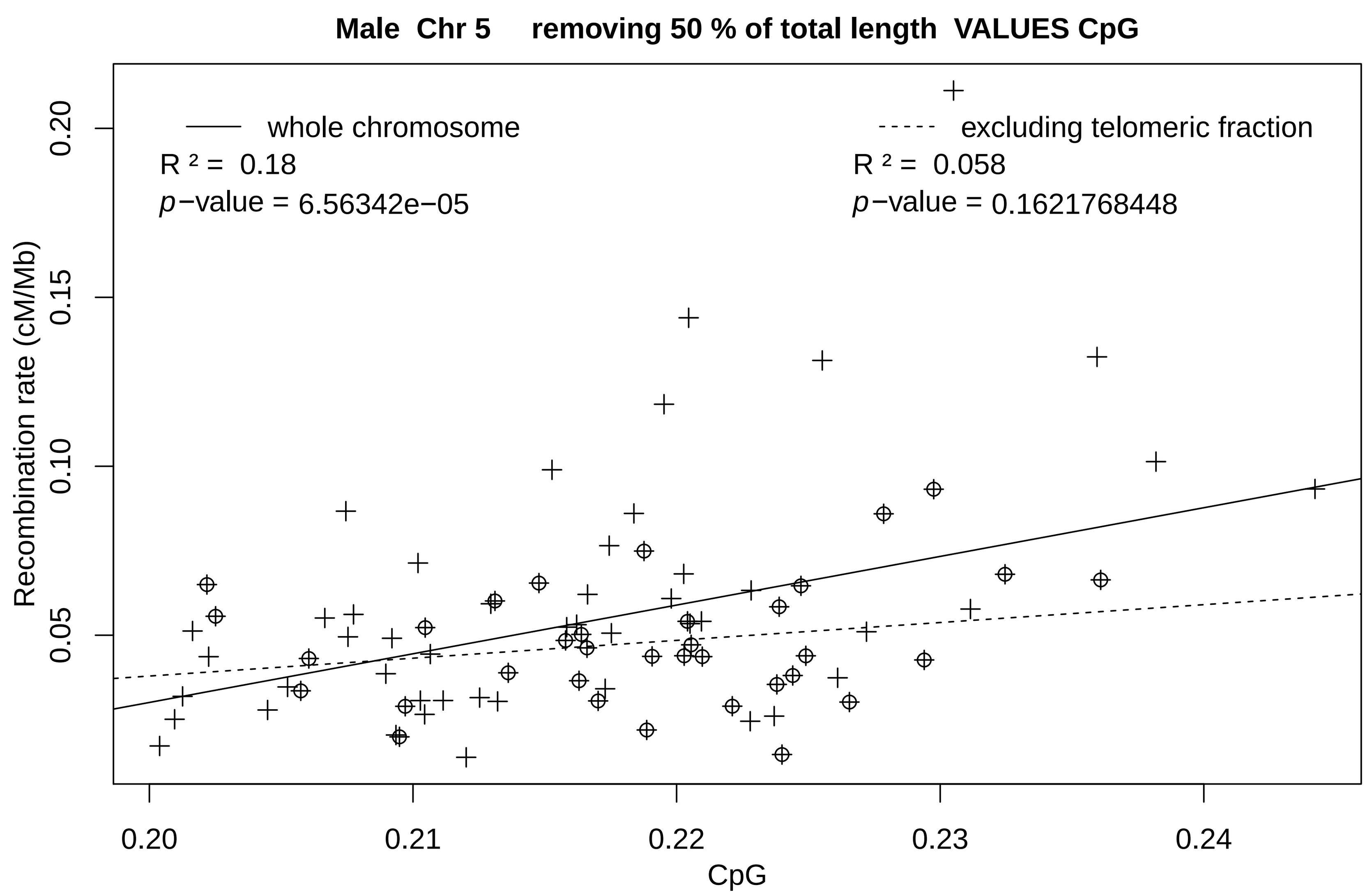
Male Chr 5 removing 50 % of total length VALUES CpG
whole chromosome
R ² = 0.18
p−value = 6.56342e−05
excluding telomeric fraction
R ² = 0.058
p−value = 0.1621768448
Recombination rate (cM/Mb)
CpG

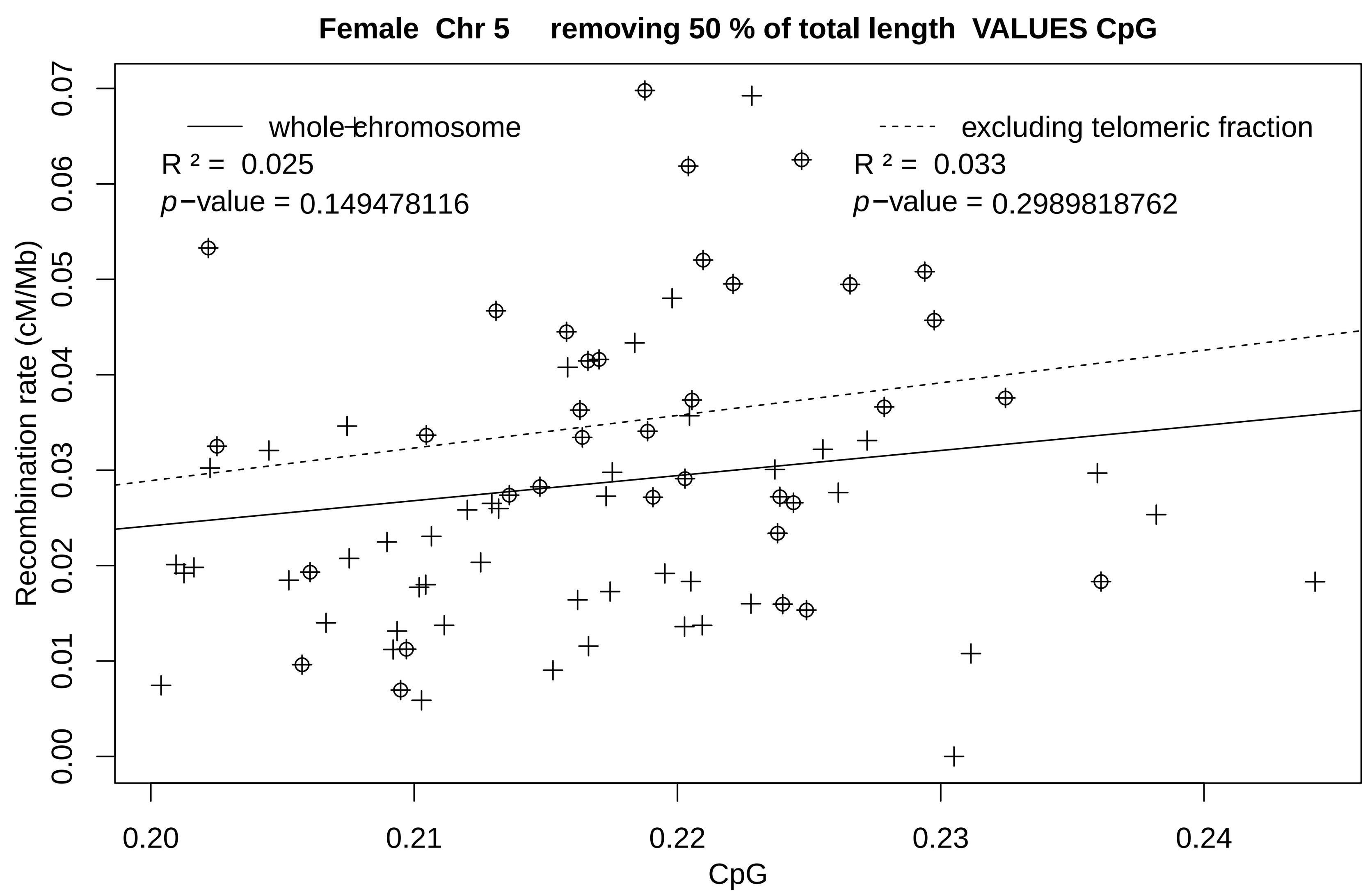
Female Chr 5 removing 50 % of total length VALUES CpG
whole chromosome
R ² = 0.025
p−value = 0.149478116
excluding telomeric fraction
R ² = 0.033
p−value = 0.2989818762
Recombination rate (cM/Mb)
CpG

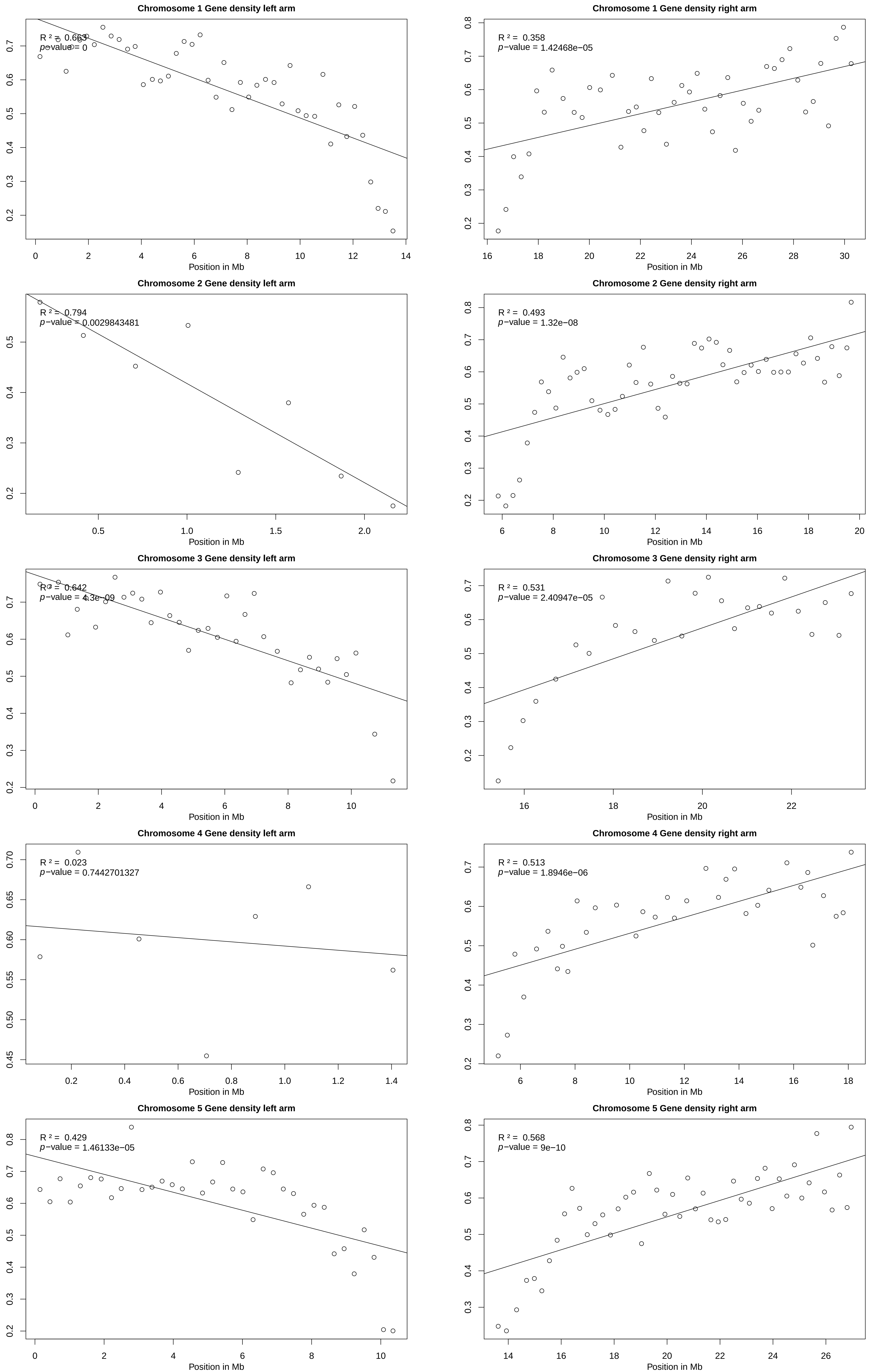

Chromosome 1 Gene density left arm
R ² = 0.663
p−value = 0
Position in Mb
Chromosome 1 Gene density right arm
R ² = 0.358
p−value = 1.42468e−05
Position in Mb
Chromosome 2 Gene density left arm
R ² = 0.794
p−value = 0.0029843481
Position in Mb
Chromosome 2 Gene density right arm
R ² = 0.493
p−value = 1.32e−08
Position in Mb
Chromosome 3 Gene density left arm
R ² = 0.642
p−value = 4.3e−09
Position in Mb
Chromosome 3 Gene density right arm
R ² = 0.531
p−value = 2.40947e−05
Position in Mb
Chromosome 4 Gene density left arm
R ² = 0.023
p−value = 0.7442701327
Position in Mb
Chromosome 4 Gene density right arm
R ² = 0.513
p−value = 1.8946e−06
Position in Mb
Chromosome 5 Gene density left arm
R ² = 0.429
p−value = 1.46133e−05
Position in Mb
Chromosome 5 Gene density right arm
R ² = 0.568
p−value = 9e−10
Position in Mb

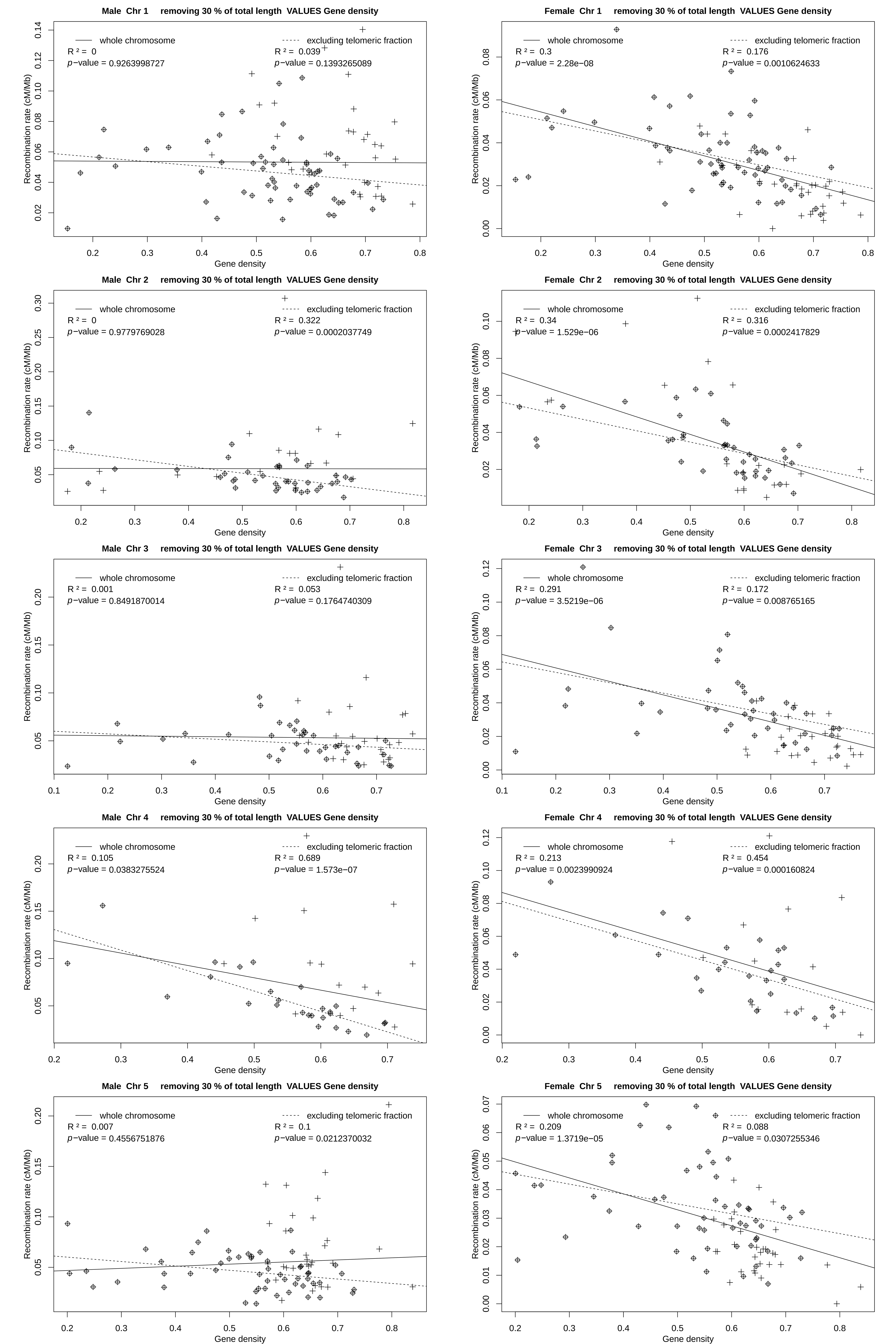
Male Chr 1 removing 30 % of total length VALUES Gene density
whole chromosome
R ² = 0
p−value = 0.9263998727
excluding telomeric fraction
R ² = 0.039
p−value = 0.1393265089
Female Chr 1 removing 30 % of total length VALUES Gene density
whole chromosome
R ² = 0.3
p−value = 2.28e−08
excluding telomeric fraction
R ² = 0.176
p−value = 0.0010624633
Male Chr 2 removing 30 % of total length VALUES Gene density
whole chromosome
R ² = 0
p−value = 0.9779769028
excluding telomeric fraction
R ² = 0.322
p−value = 0.0002037749
Female Chr 2 removing 30 % of total length VALUES Gene density
whole chromosome
R ² = 0.34
p−value = 1.529e−06
excluding telomeric fraction
R ² = 0.316
p−value = 0.0002417829
Male Chr 3 removing 30 % of total length VALUES Gene density
whole chromosome
R ² = 0.001
p−value = 0.8491870014
excluding telomeric fraction
R ² = 0.053
p−value = 0.1764740309
Female Chr 3 removing 30 % of total length VALUES Gene density
whole chromosome
R ² = 0.291
p−value = 3.5219e−06
excluding telomeric fraction
R ² = 0.172
p−value = 0.008765165
Male Chr 4 removing 30 % of total length VALUES Gene density
whole chromosome
R ² = 0.105
p−value = 0.0383275524
excluding telomeric fraction
R ² = 0.689
p−value = 1.573e−07
Female Chr 4 removing 30 % of total length VALUES Gene density
whole chromosome
R ² = 0.213
p−value = 0.0023990924
excluding telomeric fraction
R ² = 0.454
p−value = 0.000160824
Male Chr 5 removing 30 % of total length VALUES Gene density
whole chromosome
R ² = 0.007
p−value = 0.4556751876
excluding telomeric fraction
R ² = 0.1
p−value = 0.0212370032
Female Chr 5 removing 30 % of total length VALUES Gene density
whole chromosome
R ² = 0.209
p−value = 1.3719e−05
excluding telomeric fraction
R ² = 0.088
p−value = 0.0307255346
Recombination rate (cM/Mb)
Gene density

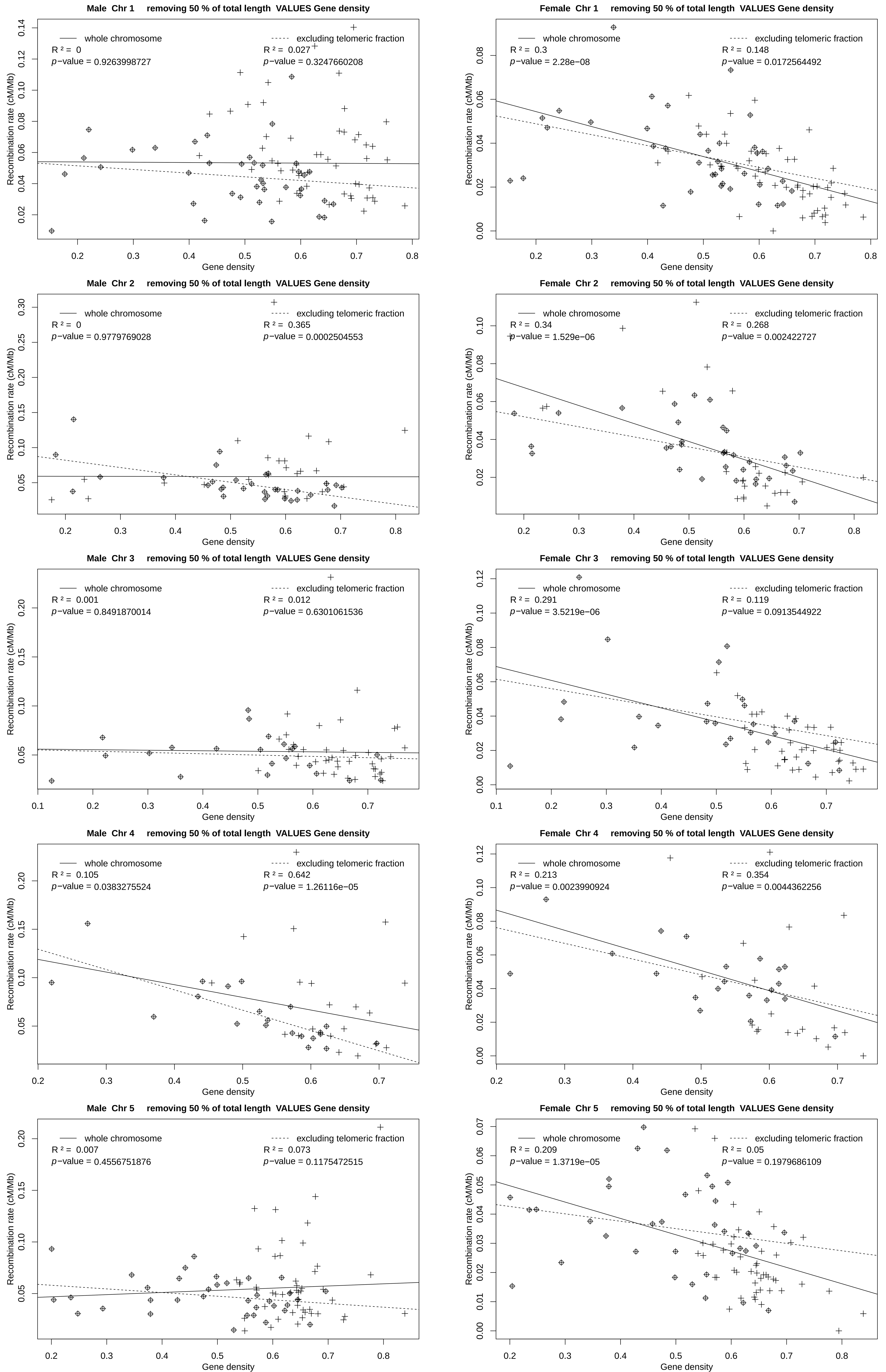

Male Chr 1 removing 50 % of total length VALUES Gene density
whole chromosome
R ² = 0
p−value = 0.9263998727
excluding telomeric fraction
R ² = 0.027
p−value = 0.3247660208
Female Chr 1 removing 50 % of total length VALUES Gene density
whole chromosome
R ² = 0.3
p−value = 2.28e−08
excluding telomeric fraction
R ² = 0.148
p−value = 0.0172564492
Male Chr 2 removing 50 % of total length VALUES Gene density
whole chromosome
R ² = 0
p−value = 0.9779769028
excluding telomeric fraction
R ² = 0.365
p−value = 0.0002504553
Female Chr 2 removing 50 % of total length VALUES Gene density
whole chromosome
R ² = 0.34
p−value = 1.529e−06
excluding telomeric fraction
R ² = 0.268
p−value = 0.002422727
Male Chr 3 removing 50 % of total length VALUES Gene density
whole chromosome
R ² = 0.001
p−value = 0.8491870014
excluding telomeric fraction
R ² = 0.012
p−value = 0.6301061536
Female Chr 3 removing 50 % of total length VALUES Gene density
whole chromosome
R ² = 0.291
p−value = 3.5219e−06
excluding telomeric fraction
R ² = 0.119
p−value = 0.0913544922
Male Chr 4 removing 50 % of total length VALUES Gene density
whole chromosome
R ² = 0.105
p−value = 0.0383275524
excluding telomeric fraction
R ² = 0.642
p−value = 1.26116e−05
Female Chr 4 removing 50 % of total length VALUES Gene density
whole chromosome
R ² = 0.213
p−value = 0.0023990924
excluding telomeric fraction
R ² = 0.354
p−value = 0.0044362256
Male Chr 5 removing 50 % of total length VALUES Gene density
whole chromosome
R ² = 0.007
p−value = 0.4556751876
excluding telomeric fraction
R ² = 0.073
p−value = 0.1175472515
Female Chr 5 removing 50 % of total length VALUES Gene density
whole chromosome
R ² = 0.209
p−value = 1.3719e−05
excluding telomeric fraction
R ² = 0.05
p−value = 0.1979686109
Recombination rate (cM/Mb)
Gene density

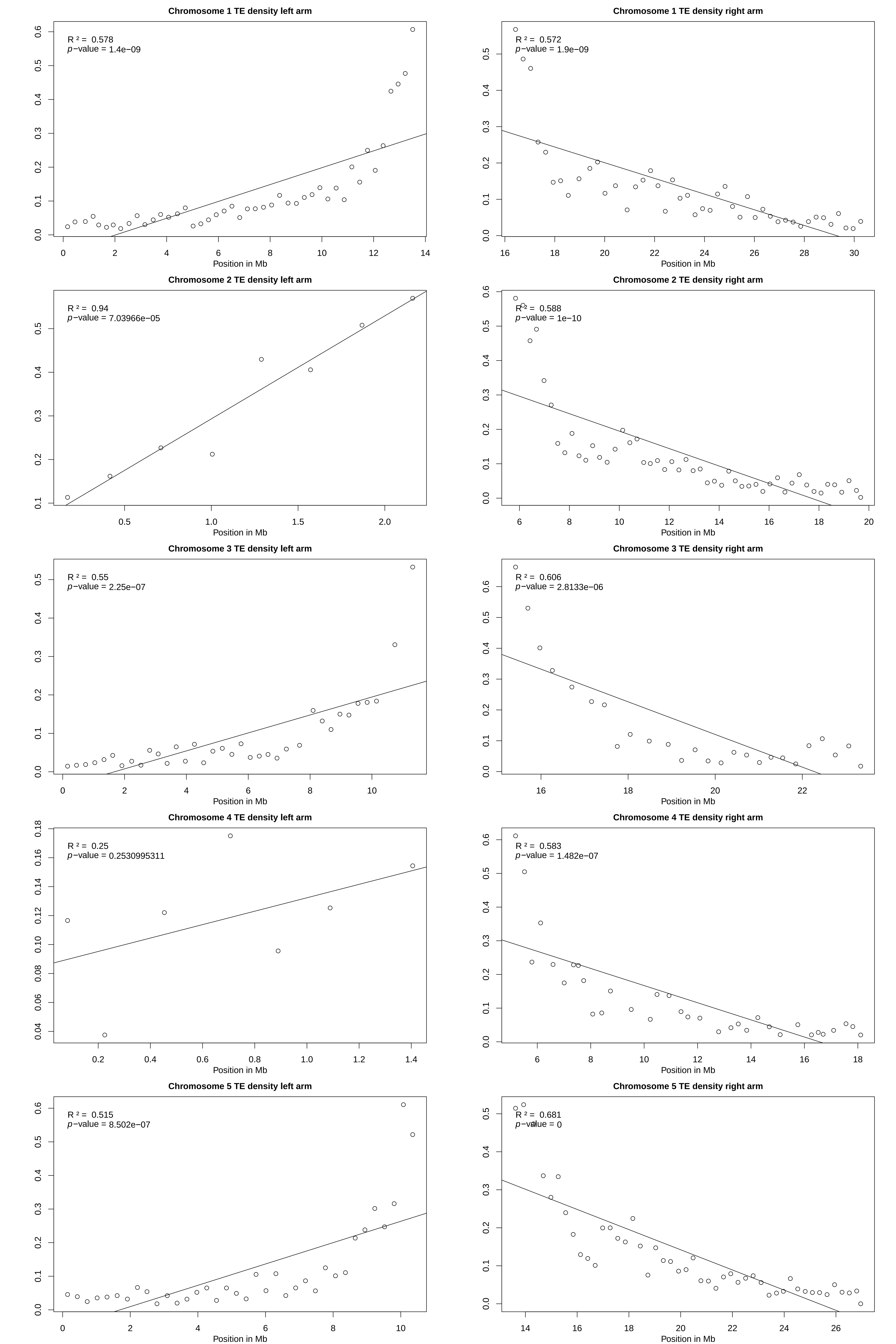
Chromosome 1 TE density left arm
R ² = 0.578
p−value = 1.4e−09
Position in Mb
Chromosome 1 TE density right arm
R ² = 0.572
p−value = 1.9e−09
Position in Mb
Chromosome 2 TE density left arm
R ² = 0.94
p−value = 7.03966e−05
Position in Mb
Chromosome 2 TE density right arm
R ² = 0.588
p−value = 1e−10
Position in Mb
Chromosome 3 TE density left arm
R ² = 0.55
p−value = 2.25e−07
Position in Mb
Chromosome 3 TE density right arm
R ² = 0.606
p−value = 2.8133e−06
Position in Mb
Chromosome 4 TE density left arm
R ² = 0.25
p−value = 0.2530995311
Position in Mb
Chromosome 4 TE density right arm
R ² = 0.583
p−value = 1.482e−07
Position in Mb
Chromosome 5 TE density left arm
R ² = 0.515
p−value = 8.502e−07
Position in Mb
Chromosome 5 TE density right arm
R ² = 0.681
p−value = 0
Position in Mb

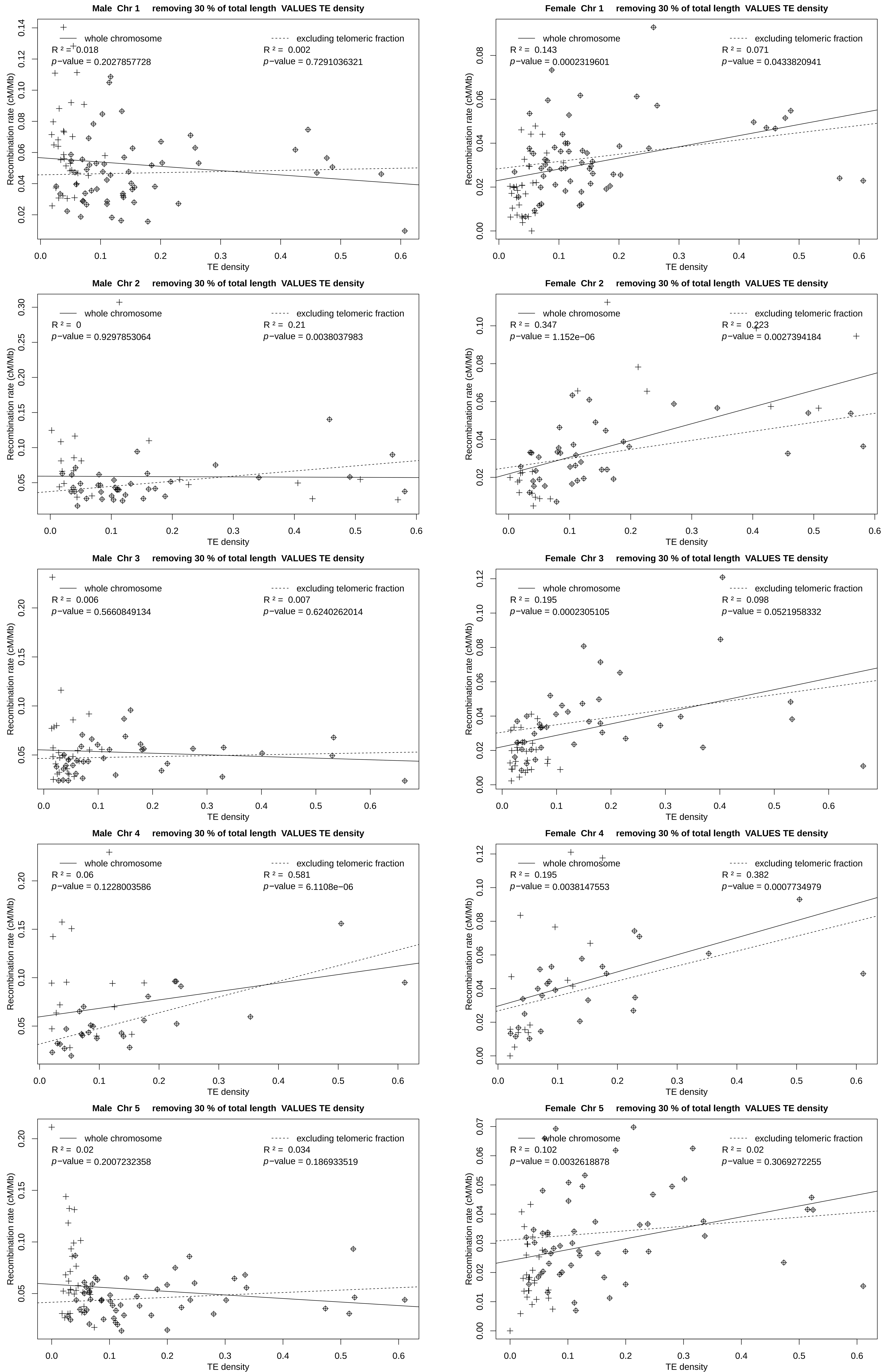

Male Chr 1 removing 30 % of total length VALUES TE density
whole chromosome
R ² = 0.018
p−value = 0.2027857728
excluding telomeric fraction
R ² = 0.002
p−value = 0.7291036321
Recombination rate (cM/Mb)
TE density
Female Chr 1 removing 30 % of total length VALUES TE density
whole chromosome
R ² = 0.143
p−value = 0.0002319601
excluding telomeric fraction
R ² = 0.071
p−value = 0.0433820941
Recombination rate (cM/Mb)
TE density
Male Chr 2 removing 30 % of total length VALUES TE density
whole chromosome
R ² = 0
p−value = 0.9297853064
excluding telomeric fraction
R ² = 0.21
p−value = 0.0038037983
Recombination rate (cM/Mb)
TE density
Female Chr 2 removing 30 % of total length VALUES TE density
whole chromosome
R ² = 0.347
p−value = 1.152e−06
excluding telomeric fraction
R ² = 0.223
p−value = 0.0027394184
Recombination rate (cM/Mb)
TE density
Male Chr 3 removing 30 % of total length VALUES TE density
whole chromosome
R ² = 0.006
p−value = 0.5660849134
excluding telomeric fraction
R ² = 0.007
p−value = 0.6240262014
Recombination rate (cM/Mb)
TE density
Female Chr 3 removing 30 % of total length VALUES TE density
whole chromosome
R ² = 0.195
p−value = 0.0002305105
excluding telomeric fraction
R ² = 0.098
p−value = 0.0521958332
Recombination rate (cM/Mb)
TE density
Male Chr 4 removing 30 % of total length VALUES TE density
whole chromosome
R ² = 0.06
p−value = 0.1228003586
excluding telomeric fraction
R ² = 0.581
p−value = 6.1108e−06
Recombination rate (cM/Mb)
TE density
Female Chr 4 removing 30 % of total length VALUES TE density
whole chromosome
R ² = 0.195
p−value = 0.0038147553
excluding telomeric fraction
R ² = 0.382
p−value = 0.0007734979
Recombination rate (cM/Mb)
TE density
Male Chr 5 removing 30 % of total length VALUES TE density
whole chromosome
R ² = 0.02
p−value = 0.2007232358
excluding telomeric fraction
R ² = 0.034
p−value = 0.186933519
Recombination rate (cM/Mb)
TE density
Female Chr 5 removing 30 % of total length VALUES TE density
whole chromosome
R ² = 0.102
p−value = 0.0032618878
excluding telomeric fraction
R ² = 0.02
p−value = 0.3069272255
Recombination rate (cM/Mb)
TE density

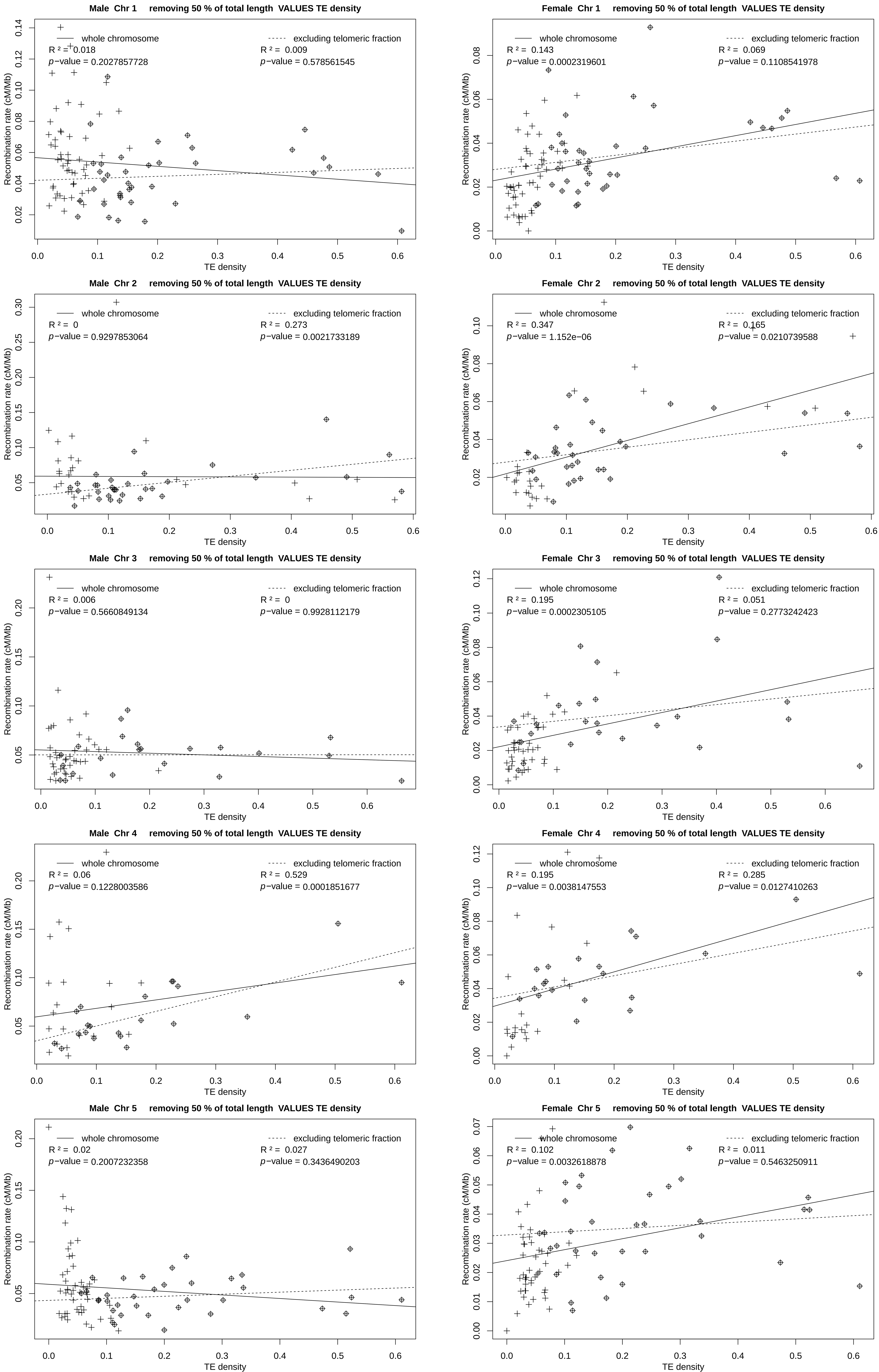
Male Chr 1 removing 50 % of total length VALUES TE density
whole chromosome
R ² = 0.018
p-value = 0.2027857728
excluding telomeric fraction
R ² = 0.009
p-value = 0.578561545
Female Chr 1 removing 50 % of total length VALUES TE density
whole chromosome
R ² = 0.143
p-value = 0.0002319601
excluding telomeric fraction
R ² = 0.069
p-value = 0.1108541978
Male Chr 2 removing 50 % of total length VALUES TE density
whole chromosome
R ² = 0
p-value = 0.9297853064
excluding telomeric fraction
R ² = 0.273
p-value = 0.0021733189
Female Chr 2 removing 50 % of total length VALUES TE density
whole chromosome
R ² = 0.347
p-value = 1.152e−06
excluding telomeric fraction
R ² = 0.165
p-value = 0.0210739588
Male Chr 3 removing 50 % of total length VALUES TE density
whole chromosome
R ² = 0.006
p-value = 0.5660849134
excluding telomeric fraction
R ² = 0
p-value = 0.9928112179
Female Chr 3 removing 50 % of total length VALUES TE density
whole chromosome
R ² = 0.195
p-value = 0.0002305105
excluding telomeric fraction
R ² = 0.051
p-value = 0.2773242423
Male Chr 4 removing 50 % of total length VALUES TE density
whole chromosome
R ² = 0.06
p-value = 0.1228003586
excluding telomeric fraction
R ² = 0.529
p-value = 0.0001851677
Female Chr 4 removing 50 % of total length VALUES TE density
whole chromosome
R ² = 0.195
p-value = 0.0038147553
excluding telomeric fraction
R ² = 0.285
p-value = 0.0127410263
Male Chr 5 removing 50 % of total length VALUES TE density
whole chromosome
R ² = 0.02
p-value = 0.2007232358
excluding telomeric fraction
R ² = 0.027
p-value = 0.3436490203
Female Chr 5 removing 50 % of total length VALUES TE density
whole chromosome
R ² = 0.102
p-value = 0.0032618878
excluding telomeric fraction
R ² = 0.011
p-value = 0.5463250911
Recombination rate (cM/Mb)
TE density
