# Supplementary material for: Genome-Wide Crossover Distribution in Arabidopsis thaliana Meiosis Reveals Sex-Specific Patterns along Chromosomes
Source: PLoS Genet. 2011 Nov 3;7(11):e1002354. doi: 10.1371/journal.pgen.1002354 (PMC3207851; doi:10.1371/journal.pgen.1002354)
Supplement: Table S1 — List of SNPs used for genotyping. * Genotyping performed with Taqman technology. (1) SNPs genotyped only in female population. (PDF) [file pgen.1002354.s002.pdf]

Chromosome	SNP #	Sequence	Col/Ler alleles
1	*CER445015	**TGGGGTCATTGTCTTCACACAATGTGCGAGGAACAAAACCATAGTTGTGAGGGTACACAACTGATGAGTAGAGAATACGATCAACCTGAATGAGAGAAT[T/C]CAAACTTGTTGAGATTGATTTTGCTATAAGAAAACCATTCATATAAAAAATAAACTTTGTTCTCATCTAACCTTGATGAGTCCTGTCTTTTTGTCAAGC**	T/C
1	CER469132	ATCTCTATAGATAATGCCACAAGGTGCTGTGGTGTAGTGGTTATCACGTTTGCCTTACACGCAAAAGGTCTCCAGTTCGATCCTGGGCAGCACCAATTGT**[G/T]**TTTTGCAATTTTTTAATAAGAAAAATGCAAACTTCCTTTTTTCTTTTTTATATACAGACCAAAAAATTGGTATGATTTACTCAAGAATGATTGTTCTATC	G/T
1	PERL_4640_Ler	CTTTAGGTCTAGCAATTAGAACCGTCAAATCCATAGTGCTAAGACAGTGTTCATCAACTTGAACCTGAAAGGTTTGCTGCGAACGGCTAACTGGATCTTG**[T/G]**GCAAGTAAAGTTATGTAGAAAGGGGACAAATGCATCATTAACTTGTTGAATTTCATTACATCAACGAGCTTGAAGTTCGTCCCCTGAGAAAAAGAGAGCA	T/G
1	PERL_7259_Ler	AGCATGACAGGAAAGACGCCGGTTCTTGACATCGGTGCTCTGGAGAAGATAAAATCCGGCGACGTAGAAATTGTACCTGCAATCAAACAGTTCTCGCG**[T/A]**CACCACGTGGAGCTCGTAGATGGTCAGAAGCTAGATATCGACGCTGTGGTTCTCGCCACCGGTTATCGTAGCAACGTCCCTTCTTGGCTTCAGGAAAG	T/A
1	PERL_8033_Ler	ACCCTAATCCTGATTTTCAGGCGAGGAATCAGTTTGACGTGAATTATTACCGTAGAGGTTACTTAGATGATCAAGGGAGGTTCCATAAAGGATCTCCTCA**[T/G]**GCGCCGGCGATGAGTCCGACCAGAAAATATAAGTTTTTGCTGCCAAAGATGCCGCCGACGACGATGCCGGAGACTCCGAAACTGATGGATCTTTTTCCGG	T/G
1	PERL_9896_Ler	GTTTTGAGAAGACGGCAAGGGACGCTCAACAAAGACGCAGCCTCTCATTTTCTGAGCATCCCACCAAAGCTTAACGTATTCTCTACGTGCACGCCACGAG**[A/T]**TCGCGCTTGAGCCTATCCCAAAGACGATGTGCTCGATTTTTGTCGGGCTCTGAGATTGATCTTGATCAGAAGATGAGAAGTATGCATGAATAGAGTTTTT	A/T
1	PERL_11886_Ler	AGCGCAAAGAGATAGGTTTAGAAAAATAGTACCTTTATACCATCATCTAAATCCTTTAAGCTATCCAAGGACTGACAGCTAAGCTGTTGACTGGTCTGTC**[C/G]**TGATACACTTGGACGAGATATGTGGTTTACATCTTTACACAGTTCACTGTTTAAGGATTTCTCAACTTTTTCTTTCTCTTCCTCCTGGTTCCTTACTGTA	C/G
1	PERL_13312_Ler	AGGTCTGAACCTAAAATCCTTGAGCCTCCCTAACTCCTCAAGCGCAATACTAAATGACCCATTCCTGCAATGTTTCCTTACCAACACATTGAGAAATT**[C/G]**GCCAAACACTTCCTTATCATCATCTCTAATCTGTTGCAGGAATTCTTCGGGAACTTTTTCGTCATCATCACGTACAATAAGGTCAACCAAAGCGTTAT	C/G
1	PERL_15479_Ler	TGGAGGATCTTCTAATGTTTCTTAAATTGGCAGCTTCGATCTTGTGCTGTATTATCCAGTAGCACATCGTCCCTAACGTCGTAATCATTATAGTTGCCCC**[G/A]**ATAGCAGTCACCCAAATGGCTAGCCACTTCTCTTCTTCACCAACAACAACAAACGACAAAGCCAAAAAGGCAACAGAGATGAGAACACAGGCAAGCCACA	G/A
1	CER428759	TTTATCTGCATTCAGGTCAGGATTCTCTAAGGTAATCAATCATACTAGTCTTGTGTTTCAATACAAAATTTGGTATTTCGATGATCTCATTGCTTTTGAT**[C/T]**ATGTCAGGGGATATTTGACATTGTGCCATTACCATCAAAGAATGAGCTGAAAGAGCTGACCGCTCCGCTGTTGCTAAAACTCGTGGGTGTTTTAGCTTGC	C/T
1	CER446172	AATTTGCCAAAATTGTATGAAGTCAAAGGTATCTTCTCAATCCGAGTGTATTTCACATACAATTTAATTTCTAAACATGGTGGCATTCTCCGTAATAAGA**[T/A]**CAGGAAACCAAGGGCAAACCCACAACACGTCAGCTATATATCACTAGGCTAAGTCCCTTACCTCTCCACAACACAACTTCAGCTTTAAGACAGCTAAAAC	T/A
1	CER445501	CTTGTTTAGTTCTGATCTTCTTCTTTAAATTTTTTTGCATGTTTCTTCACATGATCTGCAGTACTGCCTGAAGAGAAAAGTAAGTTTGTCAATAACCGCC**[T/C]**ATGATAATGGATGCTTACTTTCATAATTGCTTCAGTTTTATTTCTAATCCCTGATCTACCATTATGGTTTGGGAGAACCTTAACTGTTTTAGCGCTGTGA	T/C
1	PERL_24716_Ler	AGGTAAATAGAGACAATTAGGGTTCCAGTTAGCTTACTATCATCCTCAATCATTACTGAGAGGGAGAAGCGAAATGGAAATAATTCACAATTAAGTACCT**[G/T]**GAGCAACGATGACACAGATCCATACATATCACTTTTCCTAACATCTTCCACAAGAATATCCTCTGTTACGAGATAATGCAAGATACGAGAGACTTCAATT	G/T
1	CER465299	TAGTTAGTGTTTTGGCTTTCAATACTTGAACATGCATATAGCCAAGTTTATAGTTTAAAGATTCAATAATTGCAGTTTAGGATTCACGAATTTGTGCTAA**[C/T]**TAGAGGAATGATTGCTCGAAGACTTATAATGCTTATGGTGTTGCTTTGCATCCATTGAGAAAGAGTACTCTACCAAATAATCGTTGCTTTACTATGTGTG	C/T
1	CER474495	CATTCCCTAAAAAAACGAAATGACTCCTTTTGTAAATTGCCGTAAGACAAAATCCAATGAATGATAATAAATGATTACAGCACGTCACTTAGTAAGAAAC**[A/C]**TTTAGAATGATGAATGTCTACGTATACTTATGATATCATCAAATCAACTAATTCACTAGCTAAAGCTAATCCTTTTATTTATGTAGTAGTTTAGACTAAA	A/C
1	CER475313	CAGGGAAGAATGTGCTGGATAGCGAGTTCAGGGTTTAAAATGCGACAAAACTTGGTAACTTTTCCAGCAGCTGCTATCCGAACTTCTGCCTCATTATCAC**[A/G]**AAGTAGACGAGCATATGCAGGCACCAGATCCGTCCTTAATACATAAGGCACATGAGAAAGTATAAAAAACGACTCAAAGAAATTTATTAAGATGACTCAA	A/G
1	CER468122	TGCAGAGTTTTTTTTTTTACAAGTATTCGATTCTAAGAAAGATTGTGTATGTAGATTAAAGAGTTTCTGATTCTGTAGCATTAAGATTTTCAATAACACT**[T/C]**GATTGAGAGATGAGCATTTCACTTTTTAGGTAAAGAAAAACGAGACACATAATTTGTAATCTGTGTAAATAAATTTGTTTTTTGTTCCAAAAAGATTCAT	T/C
1	CER465478	GTGCGAAATTAGGAGAGTTAGGGCGGACGATAATCCTTTTAGTTGGGCCGAGAAAAAAATTGGGTTCAGAGTTAGGTAACTATTAAGCCCATTGGGCTACT**[G/T]**TGTATAATCTATGTCCATCGTTTGACCTCACCATTGTTAGAACAAACAAAAAAAAATAGACATCACTTCACCATTATTTCTTAAATTAATGTTAAGTATGA	G/T
1	CER465333	AACTCCTCCGTAAGTCAGCCATCGATTTGGAGAAGAACAACGAATGCGGTGTAGACGCAAAGAGATTCGCAGAGCTTCTAATGGGTTCAGGAGTAGTGCT**[A/T]**AACGATAAGATACATTGGGTGACATTCCATTGCGGATACGATTTTGGGTACTTGCTGAAACTCTTGTCAGGGAAAGAATTGCCGGAGGAGATATCGGACT	A/T
1	PERL_41814_Ler	GTGATTTGCTCAATCCATCAAGGTCATAACTTTAGTTTTGCCAAATAACTCACCCAAACAAGCAGTGTCATTTTTACACTTCCTCGTCCTCAGAGTGAGA**[C/T]**TCGGAATTTGAAGTTGTCTCGGTCTTGGTCTCCATCAATTGAGGTTGAACACCTAAACCCAATAAACAAAAACCATATGTCAACGAATGACCAACAATCA	C/T
1	CER475177	GTTGACGACAAGTTTCATTGCTGCTCCATTACCAACTTCACCCAAATAGAATTTTGACTGGATCATATAATGCAAACTAGTAACGAAAATGGTTCATTTG**[C/T]**AGAAGAAAAAAGAACTAAAAGCAAAACACAGGCAAAAGAGATACCTTTCCCATGATGTCTAAGAAAGGAGCAGCTTTTTCGTATAGCGGCTTGTCACCTA	C/T
1	PERL_46706_Ler	GGCCTGTGGATTCAGCTTACAAGGCAGTAGATCTTATCGTAAAGGTACTGTTTATACCGCTGAGGCTCTCATCTACAAATAACTCACCAGTGGGTCATCG**[C/G]**TTATTGAAAACCTTTTTCCCCCTTTAGGAACCGGCTACTCTGCTTGAGTACTCAATGAATGCAGTAACAGAAGGCATTGATGCCATCGCAACCACAAGAG	C/G
1	PERL_48654_Ler	GAAATTATTGAAAATTATGTGGCTAGTACTTAGTACGATTCACAGTTGCAGAAGAATAGGGGGGAAGATATAGAAACGTGAAATGAATGTTTTGAGAATA**[A/G]**TTTAACGAGCTGTTCAAAAGAATGAAATATAATTATATACATGACTTGGTCAATAATTCTTATGGTTGAGATGTTATGTTTAGCTTCAAGCTTTATAGTA	A/G
1	PERL_50660_Ler	AACTGTGGTCTTATAGTTTTAGAGGGTAATACGAAAATGAGAGTGCGATTAGTAATGGATAACTGAATTTAAGAAAAGTTCAAAGTGTGTGAATGAGAAG**[G/A]**AGTCGTAAGAGACAAGCATCTTCTTCTTATAACCATCTAAACCACATGCTTATGTCCACTTTTTTTCTTTCTTTCTGAGTGAAAAACACTTTAGTCTCCC	G/A

1	CH1SNP24	GCAACGGAATACGAAAATACCAGAAGTTATAAAACGCGCGAAATCGAATTGACTTACCAGAGATCAGTCTCTCGCCGGAGATGACAGTTTCTATGCGACA**[A/C]**TTGGATCGGCGAATACGATTCCGGCGACTTCGGAGAAAGACTCAAAATCGTTGTCGACGATCGCTAGCGTGAGACTTGGGCCTTTGATTAATAATGTACT	A/C
1	CER432978	ATGGTTTGACAGCACCTAATTCAACTGCCAACCATATAACAAAACCACATATAACACCTGCAGTAACAAGCTCTACAACAACCAGACCCCAAGAGTCAGC**[G/C]**CCTGTGCACAATCCCCAAGAATCATCTGCTACTTTCGGTTCTCGTGTATCAAATGTTCCATCCAATCAATTGGTGCCCAAGGATCCCAAGGAACTGGCTG	G/C
1	PERL_58052_Ler	ATAAACCCTTCTCGATGAAGAGATAAAAGTGTAGATAAGCCATGAAGCCACAAGCACTAGTGGTCCAAAAATGAATCTCCGGAACACCAAGCTCTTCCGC**[T/A]**ACGTCAAGAGTAAAGCTCATCGAACCATCTGATACAATACAGCTCACCGGAGGGACATCCTCTCTCGTGACAATCCGCTGGAGAAGCTTCTTGAACGGAA	T/A
1	PERL_61102_Ler	AATGGTGTCACCGGGAATCTAATTCCCAATGGTACTTTATCCAATGGAGGAGGCAACGTCTTCCGTTCTCTTGAGGAGCAAGCTGAAGGTCGTCATCTAC**[A/G]**GATCCTCGCCGCGAAGAAAGCTTCCGATACGGCGGATGTGTCGGACGGGGGAAGATCTAAATGGCGATCTAACGGGTACGGCGACGAAGGGTATGATTTT	A/G
1	CER444670	TGCATAGCTTCACGTAGTTGTGCTTCATGCCTTCTTTAAGAGCTCCGGCTTTGTAGCCATTTCTGTTGTCTCTCCTCTCACACTTTATATTTACACCTTT**[G/A]**CTTTCCCATTTTGCACACTCTGTGTTCACCAGCTCCTGAAACTCAATCCTCCTCACATTAAATATCTTACAATACATTATATGTGTTCAAATAAATCAAC	G/A
1	PERL_68371_Ler	CTCCATGGATGCTGATAACATCTCAAGATCCAGCAACTCTTACCTCGGAAAGCTCAGGTTCGTTTTCAGTCCTAGACATAGTAGTTTGCTACTGTTGAAT**[A/T]**TTATGGTCGTTTATCTATTGTTCAAATAATCTTTTGTGATTCCCATTTAATCAAGCTTAATGAGGTTTCTTTATCTCTCTCTCAGATCAAACTTCCTTGG	A/T
1	PERL_70718_Ler	AACCAAAACCCAACACGTAAATCAGTGAAAGGAAGGGACTTACAGTGGATTTTCCCGCGCCGAGATAGCCAGTGATAACAGACACACCAACTGAAACAGT**[A/G]**TCGCTTGATGAGTGAACCTTTCCGACAGAAACATCCGGATGGATTTGAACAGCCATCGGTGGTTCTTCATCATCATCTTCCATTTTCACTCCCTCAGCTT	A/G
1	CER426758	TCACTTGAACCTGCTTGTTCGTTTCTTTGACTTCTTTCCAGCTGAGGCTAGCGAAGGGTTAGCCCAACTTCAAACCATTGCTGCAACAAGCGTATCACCG**[G/A]**ATTCTACTATTGATGATGCGGTTTCATACATCAACACTGTCAAGGAAGCATTTCATGATGAACCTGCAAAGTATTACGAGTTTTTCCAGCTCTTTTATGA	G/A
1	CER480379	GCACTTGCCTTTTTCTACAGTTGGCGTGTTGCAGCTGTCGTAACAGCTTGTTTTCCTCTTCTCATTGCCGCTTCACTTACCGAGGTAACTCAAAATCAAA**[G/T]**TTCCTCTCAAACCAAATTTTGAATCTTGAAACTGAATAAGCTTGAATGTTATATTTTACAGCAATTATTTCTAAAAGGCTTTGGAGGAGATTACACAAGG	G/T
1	PERL_77651_Ler	CGACCCGGGAGGAAGGAATGGTCAATCCTTGTATCCTCCAGGTCACTATGTTTCAGTGTGGTGGTTGGGTTCTAGGTGCATCGATTCATCATGCGATCTG**[C/T]**GATGGGTTAGGTGCGAGTCTGTTCTTCAACGCTATGGCGGAATTAGCTCGCGGAGCGACAAAGATTTCGATCGAACCGGTTTGGGACAGAGAACGTTTAC	C/T
1	CER479670	AGACTGATGTTGTCTGATGTATACTACATCTCTTCCACCAATGGTCACCGAAGTTACAATATGAGTGCCGTAATTTTCAATAAAGCTGTACCAAAAGAAG**[G/A]**AACAAGTGGAGAAACCACATAAGAATCATGATAGATGTCATCAAGAATCACATAAAAGTGTAGCAAAAGGACAACAAAAGGAAACTGACTCTCAATTAGA	G/A
1	PERL_82572_Ler	TGCATGAAGCCCAAAATGAAAGGTTTTGAAAGAAAATCTGCACTGTGATGACGATGAGACAGTCTTATCCAGAGTTGAAATCAGCCAATAGACACTTGAC**[A/G]**CCTCAGCAAGAGGTAACCTAAACAGAATCCATTTCTGTGATCGCCAAAGCCACACGATGATTCTTGCCTAAATCATTTTAAAGACTGTATAGAGGAAAAC	A/G
1	CER426610	TGGTATTGGAGGTCAAGAGCCAATTGAGACTTTGAGGACTTTCAGGTCAGACAAAGTTTTACAGCCAAAGAGTAAACCACACGGAAAGGTAAAGAAAGAC**[C/T]**GTGGTCCGGTTTATCGTCATAACAAATGAATACGAGGGTTTTGAATGGTAGATTTAAATATGATGCAGATATACTTTGGGCAGAACATGGTTTGGAAAGA	C/T
1	PERL_89383_Ler	GTTATTAATATTTTACTTTATCAGTTAGGTTTATTAGGGGTGTTGAGTTATATATGTATGTGTAAACCGTTTAATTGGTACTGGTGCAGGAGAGATAAAT**[T/C]**ATGCTGATCCACTAGGTTTTACTCGGCCCATTGTCCCCATTCATGTTCCGGGTTTTCCGCCTCGGCGGCCAACTATTCCGCAGTTACCACCATATCGTCC	T/C
1	PERL_94924_Ler	CCTTGTACGCAATAAAGAAAATATTTTTCAATGTCGATAGCTATCTCCATTCCAATGGCCCCCCTAGTCTATTCATCGATTCACAATCTTATCAATCATG**[T/G]**TCCGTCAATACACACTTTTCTAATTTCAACAATTTTATAATTGTTGCCCTCGTTACATTTAATTGTAATATTTTTGTTCTCCTCATGATAATATTATTCC	T/G
1	PERL_98015_Ler	AAGACAGGGACGTACTTGATTTGATCATGAGAACCAAGTAAGGTCGCATTTGAAATTGCGCATACAGATTCTTTCTTCATGTCAAATTCAGCATTCTGAG**[T/C]**CAGTTTGACCAAGGTTGGAATCAAATTTGCATCGATTACTGACTGTTATCACCAGGGGAAAACAGTTTAGTTTAGTGAGATAGCAAATAGACAAATTGTT	T/C
1	PERL_101417_Ler	AACAAATGAAATAAACGCATAAAAGAGGGTCGCAAAAACCGTGAAAGAGACCAAAAATAAGTATGGATATCTGAACAGAACTTGGATGTCCTGAGACGCA**[A/T]**AGTTAGGTCGTGGCCTGGCCTATAGGCGAATCGTCTTGAGGAATGTCTCATACGCAGTCTGGAGGCGGTTTCTATGGTCCTTTCCCTTAGTGTTTTTCAG	A/T
1	CH1SNP41	GTCAGTTTTGATAGAAACGCACGAATTCTTTCAGAGTAGACTTCTCTCTAAAGGGCCACCATACTGTATGTCCCACATTTGGAATGTTTGTCAGGAGGTC**[A/G]**CCAAATCGCTTGTTACTAACTACCAGAAAAACTGGTTGAGCCTTTGGTCCAAGTTTAGACTTCTCACACATATGTGAGTTTTGTTTTTAGAGTGGATTTA	A/G
1	PERL_108653_Ler	TGGTTGTTTTTGAGATATGGCAATGTGCATTTGTAAAGAACAATCGGGTATGTCCACTATTTGTTCATCAAATTCTCAATGAGAAGCAAGAGGCACACCG**[G/A]**GTGTTAGATAAGCTGTCCCTTAGAAGACACAAACATCAAGGTGGGAAGAGAAGTATATTTCCTAAGGGCATTTCCAACCTTATTTTATTTTAGAGTAAAA	G/A
1	PERL_111792_Ler	ATTGGATTTATAATAATAATAATTTTTTTTCTTGGATTTTATAACACTAATAATTTAGCTAACGAGATAATATAGACAAATTTATATGGGATGACGGGTG**[G/A]**TCATATGCGTTGGGGTATTTTCACGGATGTAAACATTTGATTAAGATGGTAGTTAACTCATTGATGTCTTCGTAGTAGATGAACTACTGTTGAGGGCTAG	G/A
1	CER480992	CGGCATCTTCCAGTGACAATCGCGGTCGGGAAATATCTAGCCACGCCTTTCACTGCCTCTCTCATCTGAGAATCAAAAGTTGTCAGAAAACCAAAAAAAA**[A/C]**CTAAAACAAGGGAATGAAGAAGTAAGAAGGGAAGCAAACCTCTTCAGACATGTAAGCTCGATCAGGGTTTTCAACAATGGGGGATAATGTACCGTCATAG	A/C
1	PERL_119867_Ler	CAGTGTTTTTCACTGTTATAAAATCAAATAACAGTAAAAGAAGAATTATATATACATGTATAAATAAAGTAAATTTCTTTAAGGAAATTAGTACCAACAC**[T/C]**GGCGGTTCCCCATCTCCTACCGGCGGTCTCTCCATTGTCGATAGGTTCTATGTTTTCTCTTTTCCATTTCTTTATTCTGTTATTGTTGTTGTTAGGTTTT	T/C
1	CER480893	ATATTGTCAAAAAAGAATTTATAATAAAAGTCCAATATGATTGGAAATAAGAGCTACACAATACTAAGTCCATCTTTTGAGAGCAACACATGGTTTCAAA**[G/T]**CAGTCGCTTGCCAATGTAATAAACAATATCAATTTGCCATTTGAGCGATCAAAAGGGTATTGACTCGAAATAGACAATAGCCTATTGTCTCTACGACCAA	G/T
1	PERL_125322_Ler	GATAGTCCCAGTTCTAGAGTCTGCGCAAAATTCTGTTGGCAAGCCTCCAACATGTCCTAGTCTCAGAGACTATCCACTTCCGACGATGCTTTTCCAGTCT**[A/T]**GCAAAGGTTCTCCTCGAGGCTCTGAATCTACTCTGTCTCGAGCCTCTGGATCAACTCTAGTTACAGGCTCTACATGATCTATTTAACCTCACGACTCTAC	A/T
1	PERL_125426_Ler	GAGTTTCGGCCAGAAGAGACGAGATAAATAGAGAACGGCGAAGAAATTAGGGTTCCGTCGACTGAACGAGAGAAGAGAGCGATGTTTGTTTTATATGCGC**[A/G]**GAAGAACGGTCAAATATCGTGTGAATAATAAGAAACCAGAAGTTTTATAGAAATTTTTGGTTTCAGATGACGTGGATTAGGGTAAATGACGTGGATTGGT	A/G

1	PERL_126101_Ler	CAACAAGTCTAAGTACCTACCATTCATCATTAGTAACTTAAGTCATTTCCATAATATCTCTAGACAAGTAGAAGTATATACAATCTATACACCGCTCTAC**[G/A]**ATGTATGGTCCGCCATTGAATTCATCTAGAAACTGATGTGATTCCCAACACATTGGGCTGTCGGTGAAACACCACGTCTCACTATACGTTCCAAACCGGT	G/A
1	PERL_126577_Ler	GGGCTAAGACCAGCTTGAACAACAGTAAGAGAAGAAGAAGTTACTGTTATGGGTGCCTTCACGTCTGATTTGGGAATCCATTTCTGTTTAGGCACCGCTG**[A/C]**TAACATCCCGGATAACAGCTCCTTGGATCCATCTGATGACTTGATAGGACCAGGAGGCAAGCTGTTTGATCTCTTTTTTGAACGGGACCTCCTTCTTTTG	A/C
1	PERL_127515_Ler	GAAATGAAATGTGAGTGGAGTAGAGAAAATGAAAAACCTGCTGACGAACACCGAGTGGGTAAATGTCGCCGTATGTTACATTCCAGTCAAAGAATCGGTA**[T/G]**GGACTCTCCGCGGTTACCGCGGCGAAGAATGATAGGCAGAACAATAGGATGGTGCCTCCGGATCTGCCCACCGCCATCATTTTTAATGAACGACGGCGTT	T/G
1	CER480480	CATACCAGGTGCCAAATGAACCTAAATACATAGGGTTTTAGAGCAACAAACAATTGGATATTAATGGAGAATTAATGTAAGATATTAATGCACTCACACT**[T/A]**ATGAATCCATGTCGCATTGTCAAGTAATCAGACTTCCTCACTGATCTAAGTATTTGACGGAAGAAGCACATCTGAAAATTAACCTCTAGTTCCATTAGAT	T/A
1	PERL_133623_Ler	GAAAAAAGAAAAATATGGATTAGCAAAAGACAAAAGTGTAGAAGAACAATGCATAGAGTTACTATCATGTAAGGCAACATACCTCCTGTATCACCATAGC**[A/C]**AGGCTGCAAGAGAAGAAAATCAGAACATTAAGACGGTATAAAAGGGCACAAAAGTGTATGTAGTTAATTCGTAAAACAGGTCATACATGCGATGAACGGC	A/C
1	CER467835	ACCTAGCCTAGACCAAACAAGACAACTCTTCCACCAGACTAGAACCACTAGCCCACCACCTCTGGTTAAACTGATAAATTAACTAAGTTTTTGTTGACCA**[A/C]**TCCGGACAATCCGATTTGACCCGATCCAAACCAAAACGAAAATAATTGATTTAGAAAACTCGATCGAAAACCTATTTTTATAACAAGTCAATAATATGTT	A/C
1	PERL_140292_Ler	CTTTGATGCAAACCACAGCTTGTAGACCACTCGCATAACGCCTGTCTCCCATGAGCCGTATTGATGATCATTGGTTTCGAATTTGCTTGTCTAGCCATCT**[A/G]**ACTAGCCGAGCTGATGGGTTGAGATCATCCCCATGCATTCTTTACCATTCCTCTCTCTCCATAATGCATAGACTACAACCTGAAACACATCTGTAGTGCG	A/G
1	PERL_143530_Ler	CTTGCAACTTCTTAGCTATCTCTGTGCATTTCTCCTCTCTTGTGTCTTCTCTGTTTCTCTGTCTTGTTTCCGATAAATCATGCTTCTCTCTCGCGAGTTC**[A/G]**GACTGCAATGATCCGACAATTATCTTCAAAGTTTTCACCTCGGAATTTGCTTTCTCTATGTTGGATATAACTTCTTCAAGCTCCTTCCTAGCAGAATCAA	A/G
1	PERL_147948_Ler	CTAATTAAAAACCGATGGACTTCTGTGCTTGAAGCTCTAGCGGTTTCCGGTGGTTTTGGAGGTGCCTTTCGTTGCTTATCAAATCGACGATGAACGGCGA**[T/C]**GACTTATGCGTCATCTGTCCTCGGGTCTCAACTTTGTCGACGACTATTACTTCCTTACAGGAATTAGTTTTCTCTGGTGGGCTTTCATTAATTTGGGCAT	T/C
1	PERL_152362_Ler	TTTGAAATCCACGAAATAGGCGATTAAATCCATAACAAATTATACAGATCTGTTTCGATCTCAAGCATAGAGAACCCAAATCGTATACATGCGCAAGAAG**[A/G]**AAGATAACTACTATTTGACCCCACGAAAGATCAATGGATGCTGAATTTAACACAGAGAGAAGAGAAACGAACCAGGGATCAGATTCGGAAAATTCAGGAA	A/G
1	PERL_155480_Ler	ACGGCTTGATTTATCTGCAACTGAATTCAAGACATTTTCTTGCCGATCTAATCACTCTTTTTGATTACCAAACTCTTTTTGTATATGTGGAGCGTCAAGC**[G/A]**TTAGGGCACCCAACCCAATCAGCATCCGAATAGGCAGCAAGACAGAGATTCTTAGAGCGAGTCATGTGAACACCATGGGTAATGGTTCCGTTTATGTATC	G/A
1	PERL_158268_Ler	AAAAAGAATTAAACATCTTTATATAATTCAATGAACAAATGAAAGAAACAATAAGACACAAGTAACATAAAACCTTTAGAGGCAGCATAGATAGGATCAA**[C/T]**GGGCATTGGATAAAGACCCGCAGCTGATCCCATGTTAATGATAACTCCAGGCTTTTGTTTTGCTTTCATAGCTTTGATCTACACAAAAGTTGAAAATCAT	C/T
1	PERL_160456_Ler	TATTTACTTTTCATTTCAAGTTCTCAGGATTTCCGAGCCGTTAAATTTGTTTGCATAAGGGAATTACAGTTGTGCTTGATCATCGGAGACCGTAGAGTAG**[C/G]**CGATTTGGTCACTAGGGAGCTAACAAAACGAGGCTCTTGCTCGTTAATCTTTCCACCAACTCATGGTATCGGGGCCTTCCTAACCCAAAAATACGGTTAA	C/G
1	CER481644	ATTCTCACATGGTGTTTAGAAGAAACCATTCAAAAATTCAAAAAGAAGCTGAATTCTTCATGTAAACTACCGAGCCAATGGGGTCACTTCGGTACCAAAG**[G/A]**TCAAGCTTACTTCTATAGAACCTTCTGAGTCCATGTCTCGAATCGCTCCTCCCCTTGAATTTTCAGATGTCAAACACTCGTTTAATTCAATAACAACTTG	G/A
1	PERL_166550_Ler	AACACCATTGAAAGAGTTTCTCGATAAGTACGAGCTAGCTCTTCAGAAGAAGCACAGGGAAGAAACTCTCAGCGACATAGAGTCTCAAACCTTGAACACC**[G/A]**CGGAGCTTAAAACAAAGTGTTCTTTCGAGACACAGCTCTCGAGAATCTACACGAGAGACATGTTCAAGAAATTCCAAATAGAGGTAGAAGAAATGTACTC	G/A
1	PERL_168980_Ler	CTCGTCGAGAAGCACATCTGCCACCAGCTTGACTTTCCGAGATGCTACCACTTCCAAACTGTTACCAAGCGCAGCTACGACAACATTTGCTTTTGCCCGC**[T/C]**TCAGGACATCAATGATAGCAACAGCTTCCATTTCCTCCGAGCCATCTGCAATGGGTACAAGAATCTGAAAAGGAAAAACATGATCTCTCAGTGTACGGAA	T/C
1	CER425903	ATCCAGTTATAATTTGACTCATAACCCTTATAGAAACCCCGATCGATCCCTAAATATTGATTTTAGAGACTCAGTAATACTCGTAACCGCGGCTCTCCCT**[G/T]**AGTTGAACGGTGGCTGACGTTGGGGGCACCCAAGACTCACGCATAGGTTCATCGGCAGGGTAAACGACAAGCTCACGGATGTTCATCGGTTGGATAACAT	G/T
1	PERL_174514_Ler	TTATGTCGTTGTAGACAAAAACTGCGATTAATCCTCCTTCTTTGCGAAGAACACGTTTTGCTACGTTGTAGAATGGTGCGAGATCGAAGTAGTGAACGGC**[T/C]**TGTGCTGCCACGATTAGATCAACGGAGTTATCTCCGCCGACTAGAGCAACCATTTGGTCTTCGGACATTGTTGTGGGAGTGTGATGGTAACTGATTCTTT	T/C
1	CER480463	TTGTTTATTCTTCACTGACCTTTGTCACAACAAACATAAAACTGGTGACAATGAAACAAAAGAAAAATCATAGAAGCCTAGCACAAGTTGTTGCTGCAGA**[C/T]**TTCGTATGAGAAACACATTGTGCTATCGAGAGTTCAGCGCAAAAATGGACTATCATATTGTGTAGTTTACAAAAAGAAAGGACGAGAAAACAAAAGCAAA	C/T
1	PERL_180543_Ler	GAGAACTAGAAGTGGATTCTTCGACGAATCTCACTGTGACGTGAAGGTACATGTCAAACGGCGCTGGAACTCGTTCGGATGCTAAAGCCATCTCAACGCA**[A/G]**AGACGAGTGCCTGAGATACTGTCTGGAAAGATTGTTTCGAGGAGAGGAGAGGTCTTGTGTGGATCAAAGTTAACGACGTCTTTTAAGATAAGTTGAGTGA	A/G
1	PERL_185054_Ler	TGGCAGATTCATTGATGTGTTTGATTATGTTTAGCTGAAGAATTCTGTGATTCATCTTTAACCATTGTGTTGATGTTGTTGGTGTTATGATGCAGGCGAC**[G/A]**TTTGACTGCTTACAGAAGACATATGGGTTCCTTACACCAGAGTTCTGGAAAGAGACTAGATTCTCCAGATCGCCCTACCAAGAGCACACTGATTTCTTGT	G/A
1	CER428933	TCACGTTTTAACAGTAAGACCCACAGTACGCAGACAATGAACTTCAGGTTGTAAAATACACATAAAATAAAACACACCACAGAGTACGCACTACAAGGTG**[C/G]**AGGCAAACACACTAGAAAAATCATAAAAAACAAAATTTGAACAGTCTATACAAACAAAAAATATAGAGATTGAAGAGACCGTTGCTGCAATGTAATAACA	C/G
1	PERL_191685_Ler	AATAAGGAGCAAGCAAAAGCCAATCCGGTTAGGAGAAGAATGGTGGGTTTCATTTGGTTAGGTGAAAGTGAAAGAAACTTACTTTGAGAATGCCCTTTTC**[T/C]**GGGTCTGAAGCTATATCGGGAATAGTTGAACCCGCCATGAGATGAAACAGATTCAAAATAAGGTTGCTCGATTTCCGGAGAATGTTGTAGGCTTCGCAAC	T/C
1	PERL_195268_Ler	CATTTATTACAGAAGAGGTGGTCTTATAGCAAGAAAGGCACAAGAACTAAACCTAGTTCTTCAAACACCATAGCTCCTGGTTCAAAGTCAAGACCACTCG**[G/A]**AATCACTTGCGAACTGTAACCCCCTCCTGCTGAGTTTTCCAATTTGTACAATGTACATTATCAAGAACCATCTTCATGTTCCTCCCGGTGCTTTTGTATT	G/A
1	PERL_199396_Ler	TTTTTATACCGGACAAAACAGGCTGAATTCCATTGAGGACAATTGTGATGGCTAAAAAGGGAGATAAATCCGCAACGGCCTCAGCCACTGCTGGACTATC**[A/T]**GTAAACGCATAGCTGATGACGTGACGCCACGATAAGACCACGATGGCTTCGAAAACCGATAGTAAGAATGATACTCCCGTTGTCACGACTGTGGAGAACG	A/T

1	CER446517	CGAATATGCTATGGTAATATATATCAACTTGAAAAAGAATATAGTTTCAATAATAAATTTTGTGTCTTTTAGTGTGAGTACCTCGATTGGATCGCAGACC**[T/A]**GTGTGGATTCCCACATGTTGAACATTGGAGAGATCAAGAAGTAACTCGCGGTTACCAGAGACTTGGTAATCAACCAGAAACTTTCCGTGATGAATGGGAT	T/A
1	PERL_206982_Ler	AATTGAGATTCGGAGAAGAAGAAGAAGCTATAAAGGTTGTTACCATAATGTCCATGTGAAGCCAATTGGCGCCCAAATCGATCATCCGCTTAGCCTCCGC**[G/C]**GCAAGATTTGCAAAGTCCGATGACAACATCGACGGAGCGATCTTCGGCGACACACCCACCATCTTCTCTGGTTTCAACGAATATGGTAGAGAAGAAGAAT	G/C
1	PERL_211165_Ler	GTGCATCAACTTCACCGGGAACTGAATACTCCATTAACGGTGATCAAGAGTTCGTTGAAGTCACAATCGATCTTCAAGACGATGACACAATCGTTCTTCG**[T/A]**AGCGTCGAGCCAGCAACCGCCATTAATGTCATCGGAGATATCTCCGACGACAACACCGGAATAATGACTCCGGTTTCGATTTCGAGATCTCCGACGATGA	T/A
1	CER425307	ACTCCTTAGGCCAAAGCCACTAGGAACCAAAGGTATAGTAAAATTTGTTTCGGTAAGGTCATAAAATATGTAATTAACATACTTGCTTTGATAACACAAG**[T/C]**GGAGAAAGGCATGTCGAGACAGCACGTTGCACAGATTCTGATGGTGTATTCAACACCTCCAACAGCTTTTCCACAACATTATGTACTTTAGGATCATCCT	T/C
1	CER431810	CCTGAGAAGGGCAAGAAGAATCGCCAGGGCAATAGTTCTGATCAATGAGGATAGGATATGAGACATTAACCATAATAGCTCCTAAGAACCGGACCCTCTC**[C/T]**ACGAACCCGTTGCTATGTCGCGGCCACGACTTGATCCTAAGCCCATTGTCCGTCCGTACAAACACAGCATTCTTCACAGTTACGTTTTCCACGCCTTGCT	C/T
1	PERL_223567_Ler	CTCACTGGAGAGGTCACTGTGACAAGAACCGTGAGAAATGTTGGACCGGCCCGTTCAGTCTACAGACCAGTGATTGAGTCTCCTCTTGGTATCGAGCTTG**[A/C]**TGTGAAACCGAAGACTCTTGTGTTTGGTTCCAACATAACAAAGATTACTTTTAGTGTGAGAGTGAAATCGAGTCATAGAGTTAACACTGATTTTTATTTT	A/C
1	PERL_227196_Ler	ATGTATTCTCATTGATCACCACCGTCACCTCAAATCCTTTTCTTAAAACACTTTCCAAATTGGTCACATTCAACTCTTTCTCTTTTGTATCGAAACTCGT**[G/A]**GGAACCTTCACGGTGAAACTGGAACGACAGCTCTCAGGATATTTTTCAGGGCTTTCAGACACTGAGATGGGACCTATCTCAGGACATGTATAGCTAGAAA	G/A
1	PERL_230392_Ler	TAACTTTCTCACCACACAAAGCAACTCAACGCCAATCTTATCATCCTCTCCATTATCACTCTCAGGTACATCTTGAACAATGACGCGCATGTAGTCAGCT**[A/T]**GAGCGGTGGCGATTGTACCGATGGCTTGAAGCTCAACGAGGTTAGGTTTTTCGTCTAACGGGAGAACCGGAGCTAAGAAGGATCTGAGAATAGTCTTGTT	A/T
1	PERL_232983_Ler	TATCAACTAGTCTCAACAATTGTCACAAAGCGATGCACAAAAACTCTTTCAATGGTGAGAGGAGAACTCGCGGTAATAGCGGCGGGTAATGGTGAAGTCT**[C/T]**ACTCTACATTTGGGCAGCTGTTGTGGCTTTCTCCGTCATAGCAGCCGTGATTTTCTCATGCAGTGACCGAGCTTCTAAACCTCACACTAACGACGATGTC	C/T
1	CER465324	CATTAATTGACACAACCAGAACACACGGACTATGGGCTTGTCAAAAGATAGTTGAACATGCGTGAGTCGAAAATCCAGAGAAAACTAAAGCCAATTGGTG**[A/T]**ATCGCTGCTTACCATTTGAAGGAAGATGAGGCCAATGCTATAGATATCGAACCTATCTGGCAAATTCATCTGTTTGGACAAAACAATAAGTATCAAATTC	A/T
1	PERL_238431_Ler	TATGGCCATTCAAGAAACATACGCTTGAATGTGACCCGAGTGAGAGACTTGTGAAAGAAGCAAGCAGAGACTTTGAGACTGTCTATGTAGGCCAAGTTTG**[T/C]**CTCTCGTGGGAGATGCTTCGATGGCAATACGACAAAGTCTTGGAATTTGATTCACAGGTTACTACTTATCAATACAACTTAGTAGCTGGAGAGTTTCAGT	T/C
1	PERL_241367_Ler	CGCTGATTAGGAGCTCAAGCTCTTTATCATTGAAAATTGATACAAGCTCACGAGGTATCAACTCATTTAAGCCTTCTAGGAAAGCGTTGATTTGAGGGCG**[T/A]**ATAGCACTGGTAAGTATGTGGTCGGCCACAAGGTCAACATATTCATGTTTTGTTTCTTCTGTTACCCGTATGTTTCGTCCTCCAGGTTTAAGTTCATAGT	T/A
1	CER425902	TGTTAGACATGAATCTGCTAGAGACACTGCGCACAGAACCGATATTCCTAGGAAGCAAGGCCAAGAGAAGCTTGGAAAGAGCGATATGGAACGACACCAA**[T/C]**TTCTTGGCTGTAAGTGTTTTCATTACCCCCAATTCATATTTTGTTCTTAAGTGTTTTGATTAATCTGTTTCCCCATTATCCTCTTGTGGTTAGTCTGTGG	T/C
1	CER473460	AGCCCTCTTTTTCATGGCTCTTCGATCCGCACTCTCAATGCTGAAACATGATTCTGAGGGTAAAGAGTTCATGGAAAAGATAGTGAAGCGGCTGCACGCA**[C/T]**TAAGCTTCCATATGAGAAGCTACTTTTGGCTCGATTTCCAGCAGCTCAATGACATTTACCGTTACAAGACGGAGGAGTACTCTCACACCGCGGTAAACAA	C/T
1	PERL_248424_Ler	ACGGTGGAGATTCTACGATGATACACGTGTAAGTCAATGCCGTTATGAAGATGTAGATCCGATCTATCCAAATCAAAGTTACAGAAACTGGCATGAGGAA**[G/C]**GTAAACCACCGCCGGAAGATGTAGATCAAACAGAGGACGGTGATAATGGAGAAGGAAGTAAGGTCCGTAACGGCGGTTCGGAAACTGAGAAAGTTGAGGT	G/C
1	PERL_251951_Ler	AATTGCAGATATATAGAAGAGGAATCATTGATTTTGACTTACTCTCCAAGGCAGCCATGTCTATAAGGTGAGGTCTATCTTCTCCGTCGATATCTTGAGC**[C/G]**ACAAGGTTCCTCATCCAATTAGGGTAGTTCCACAATGTCAATGCGCCACAAGATTGGTGTCCCATTGAAACAAGTAACTGCTCAAACCCAAGTTTCGAAG	C/G
1	CER468291	CATGGACATTGATACCTAACATGTTACCTGAAAGAAGCAGTGGAGGTGGAGGAGATCAAGCAAAAGAGATTGCTGCTGCAACAGCGGCTTCAGAGGCTCC**[A/G]**CCGCTTGTAGCAGTTGTAAAAGACGAGCTTTATGCTGCGAATTACGCGCAGCAAGAGGTGAAGAAATACGATAAAAGGCTTAATGTATGGAATAAGGTAG	A/G
1	PERL_255454_Ler	CAATTTAATTTGTGAAAATATATTGTTTTTCTTTTTGTCTTTGTAATGTCTTTTTCAGATCTACTCTTAGCTTATGAAACGAACTCGCGCTCATTTGGCT**[C/G]**CAAGTGTGGAGATCTTGTAAGTTTGTTTCAGCAAATTAAGGTTCAAATTTTTTATGATACAAAGTACAAACCCTTATTCATATAAAATGGGCCATTTTTC	C/G
1	PERL_257106_Ler	ACAGAAGATCAAAATTCCCACTATAAGATTCGTTAATGCTGCAACTGTCTTCTACAAATGTTCAAAGAGTTTTTCTTCTCATAACTCTACAGTATCACGC**[G/A]**CAGAGCATAGCGAGTGGATAAGGACATGAAATGGATGAATGGGAAAGAAGTGGGAGATACCTGAAGCATTAGCTGCTCCGCTAGCTCAGAGTTTTTAGAT	G/A
1	PERL_258427_Ler	ATTACAAATACCTAGGATCCATGTTGATAAGATCACACAATTCAATGTTAATAGCCCAATCTGGACCAATCAGCATATCATTTGTAGCCCTTTCTGCACA**[T/G]**GCAGCCGCATCATTTGCCATCTTAACAACTCTTATCAATCAACTCCCACAAGTTTCCAAATTTTTCCTATGAAGACACCATACTCTACTTTAGTCCAAAG	T/G
1	CER468885	AGACAGATACGTCCAAGCAGCAACAGATCACCACAGGACTTGAAGCTCCAGTGGGATCTTCCATTTATGGGAGCGACGGACAAGCGAATGCTCGTCTGCC**[C/T]**GAGGAGTTGCATACCAGTGTTAGCTCACAGCCACAAAAATCAGATAAGCATGAAAAGGTTGCAAGTAGCAAAGGGCCATCAGCGCCTCGTAAAAGAAATT	C/T
1	PERL_262340_Ler	GATTTGGTTGGTTGTGGCAGGGGAAAGAAATGATTTGGGGATCTAAAACGTTCATGGCGTTTGGATATGGAGTTAGACTTTGTGTAGGTGCAGAGTTTTC**[A/G]**CGGCTTCAAATGGCAATCTTCCTCCATCATCTTGTGGCATATTACGATTTCTCAATGGTCCAAGACTCCGAGATCATTCGTTCACCATTCCATCAATACA	A/G
1	PERL_264296_Ler	AACAATGCCTTGACCTCTACGATTTAAAACGACAAAAACATCAGGTAATAGGGAAATTAAAAACCTTAAAAAGAAAACAAAGGGAGATAAGACACCGCCA**[G/A]**AAAGGTAACAGGAACCACGACAGATCAAAGATATCTTTCAGAATACACTGAGACAGAACTAGCCTTCTTTGCATCTTCAAAAAACCTAAAAGAATGGGAC	G/A
1	PERL_266525_Ler	AGGTGGTAAACCTGACGAGCGAGCCGTCGATAAGCAGCTTTAATCTCCTTGTTATTGGCAGATTTAGAGACACCGAGAGTGGCGTAGTAGTCTCCGGAAG**[T/C]**AGCAAATACGACGAGGGAACGGCCCCGTCGATTTCGATAAGGCGTAGACGAAGAAGACGAAGATGAAAGCGACAAGAAGCTCTTGTGATACCTGAAATTG	T/C
1	*PERL_268547_Ler	TTCTGTCCGAGAAAAGGAAAGCTCTGTTCGAGCCACTTGAACCCATTACGAACTTGAACGGAAAGCGACCAACTGCGGCTGATTCATTGTTGCCACCGCC**[G/A]**GATTTCGAGACTGCAAACTACCCAAAAGGCTGGTTGATCGGTAAGAAGAGGAAGCTTGTGAATGTTGATGTAGTTGAGAGCATGCGTAGAATAGCTGTCC	G/A

2	*PERL_269150_Ler	ATTCCCCACTTCCGATCCTTCATGACTCACAACAGCTATTTCAGACATCAAATCCTCCCGCTGGAACCGCTGAGACAAAGTCGACGATGAATCCAAGTC**[G/C]**AAACTAGCCTCATGGGCATGCGAACCCATCGCACTTGAATACACACCACCTGGTCTCCCAACATCTCTTTGTTCCACACGCAGTCGCTTCCCGTTGGAT	G/C
2	PERL_270864_Ler	TCCTCATGGTCTCATGGTCTCATGGTCATGGAGCTCCATTGTTGGCGTAAGTGTTGACAAGGGCAAGAGCATTACTCGTTAGCCTCTTCACTTCCTCGAG**[C/A]**CGATCACAAACGGTCGTCTTAATCAATCCTCCTTCGTCCATATCTTCAAATCCATCCGTACACGTGTCCTCATCCGTCAATGCTGCACTCATCCACGTCT	C/A
2	PERL_273021_Ler	TTGGCGTTGTTGCCCTAATCGGTGTCCCGTTTCTAGAAGGTTCTTGGCTCGCTATAGGAGTCATTTCAGTTCCCATGTCTCTCATTGACACAGACCTTGC**[C/T]**GTTGAAGGCGGTTGAGCAAACGCAGTTGCGACTGACGAGTCATGACGGCTGAGATTAACTGCAAGAAAGCAAGTAAAATTGGCTTTAGAAGATAAATAGA	C/T
2	PERL_276304_Ler	ACTTGTTCTGTTACATACCACCTCGTGTGAAGGTAAAGAGACTAGACTATCTTCAGTACGTGAGGAAAAAAGAAGATGGAGCATTGATATACGCGGCTCA**[T/C]**GCAGATTACTACATTCTTTTGCGGGTTTGTGCTAAGGTAGCTGAGATTGATGTGAGGAATATGCATAGAGGTGTGTTGAGCTTTGAGAGGAGATTGGCTT	T/C
2	CER427280	CATACCAGTACTCCAATATATGATCCCAAAAGGGTCCACCTATAAACTTCCCTTCACAAAACGCTTCAACCGCTTTTTCGATAGGATAATCCGCGGTTTC**[C/T]**TCAGGAGCTAGCTTTTTCCCAAAATGCCATAAGGACACAAACATGTCCTTAGGGTTCCTACAACAATACACAATCTTACAAGACGAGCTCTTAACAGACT	C/T
2	PERL_281027_Ler	CGTTTACTAAATACTAAAACAAAAAAAGAATATATATTATCTCCGACTTATAATTACATATACAATATTTGGGCCCTTAAACACATAAGACCAATCGAAT**[A/T]**GCCTTGCTGTATCACCATTCAACCCCATTGTATATGGTTGAGTTTATCCTTATAGTTAAAACAATCCAACTATTCCCGACATCTTAAAAAAAAAAAAAAA	A/T
2	PERL_284251_Ler	TCACCCATGACCCGAGAAAAAGACGACGTCGTTTTCTCATTTCTTTAATTAATCGAAACGTAAGTCTCTATCACCACCTCTGTCATCTCTTTCATCGGAA**[T/C]**CGTCGTAATCTCCCTTGTCGTCTCCCTCATTCTTAACCACACTATCATCGTCCCCCGAACTTCTGTTTTCACCACCAACATTTTCACTCTCGACATCACT	T/C
2	PERL_288159_Ler	ATTCACTTTAACATTTGCACTTCGGGAAATTAAAGATAAATTGAAAAATCAAACTCATCATTATCTTCTTGAATCGCTTATAGGTACGTAGTAAAGGCTG**[G/T]**CCACTTTGTTGGAGAAGACACTATCTTCACCAGGTCTGAGCAGTTTGTAAAAAATGTTACTTCTCGCTTATCTTGGCCAATCATGCATCTCATTGCCCTA	G/T
2	PERL_291873_Ler	ATTTGATCCGTCAAGAAGTTGAGGGTGTCGATCGACACCCATCTGGTATCGCTTGACACCTACGTCTACGTTGCGAGCAACGAGTTGCGCGTTGAGTTGC**[A/G]**CCTGCTCCATTATCTGATCCTCATCATGGATGATTATGTGATCATCTAGTCTCTGTCCAGTGACATTATAGGCTTGTCCATCATCATCTCTCAAAACTCA	A/G
2	CER442147	CGGTTTATCGATTTTTTCAATTTTTCTAGGGTTTCGCGCCGGGATCTAAAAAATCACAATCATGCCGGTGAGTTTGACTCCGAACGCAATCACGGCGATA**[C/A]**ATGACGGCGACGTGAATTTGAAGCCGTTGTTACAGGTTTTAGAAATCAAAATGATAGGAAGAAGTCAAGAGAGAAGCCAGGAGAGGTATCGTTTCCTCAT	C/A
2	PERL_298019_Ler	TGTCAGCCCAGTACATATATAATCTTATGAATACAAAGACTCATCGTTAACGCTGAATTACATATCTCCTTCGTACCATTCCTTCAGGTCTTCCATGGCT**[T/C]**CCGAATCAACATGGACTCCAGCTGACTTCGGAAGGTTCGAGATTATGCTATCATTGTCAACACCAGACAAAGTAGACTTTGGATACATTGCCAAGTATCT	T/C
2	PERL_301285_Ler	ATTCATCAAACTCTCCTTCCTTCTATTCACGTCTCCAAAGACTTCACGGTTCCACTTTTTTAATTTACTCTTTAGAGCTGCCAATGCTACAGGCGTCTCT**[C/A]**CTTCCGTATTCCAAGAAGCCTGAAGCAAATCCTTAAAACCACTATGGGTAAGCCAAGCCGCTTCAAACCGGAAGGGTCTCCTCAGCGGATTACTCCGCAC	C/A
2	PERL_301533_Ler	TAATAGCAGTTAAAACAACATGTTATATAAAATAATAACATGCTAACATATTTAATATCTAGATAATATTTGTATGAACGTGCCATCTCAATCCACGTTT**[T/C]**CATTCACGCCACCTCTTTTTTTGTCAACTCACCAATACTGATTTTAATATCTTTGTTTCGATTTGATTTTATGTTTCCATTATTATTCTTATAGTTTTGT	T/C
2	PERL_302774_Ler	TACCAAACCTGCACTAGATAACCTGAGCATCCTTAAACTGTTCAAGCAGGGAAACTCCAGTGAGATCATTGTCAAAACCATAGATGTCACACAAATCTCT**[T/C]**GGTGGTAGACTCTATCTGACACCGCGAATGAAGTAGATAAGAGCTCCTTCCTGAGCATTGCGTTTCCTGTCATTCACATAGTAGACCCTAACAGAAACCA	T/C
2	PERL_304972_Ler	AGGATTCGATTAGAATTGAAGCAACAAATGAAAGATTACTTCCAATTCACTACAAAATTATTTCAATAAATCAAGGATTCAAGAAGAATTGAAGCGCGAA**[C/G]**GATTGAACGACGAAGATGCAAAGGTCTTTTTCGGGTCGCCCGCAAACCCGCTACATTTAATGGGCATGTCTTGTTTAATCCGCAATCCAAACGGATCATA	C/G
2	PERL_307094_Ler	CACATTTTTTGGATCAGTAATTGTACGTATATACATTGAAAATTTTCTGATCTCCGAATTCTTGTTATTTATAAAACATGACTTCTCGCGAGTCTTCTAA**[G/T]**CACGGAACCATCCGTTCAAACCCTCCCATAGTCCAAAAAAACAGTCCTCTCCTTCCATCTTCATCCGCAAAAAAAAAAATTTTCCTCGTCGCTTACTTGCC	G/T
2	PERL_309922_Ler	TAAATCCAAGACCACGATTGTCTCTCCAATCAAAATTTGAATCCATTCAAAAAGTCGTTGACACAAATCTTCCGCAAAGCTATCGAGAGAATGGCAGCTT**[C/G]**TAACGGTCAAATCTGCAGCTCGATTGGCTTCCGTAATCGTTTGTTAAGTCTTCCTTTCTTAGCTTCTGAACTAATTGATTTAATGCATTTCCTACATAGT	C/G
2	PERL_312769_Ler	GATCTTTTCCATGATGGTTGTGCACTTAGCAGCGTGAATCTCCTGCTTAGAACGACGCTTCCTACGATAAGGGACAAGAGGAGTAAGCAGATTCGAGGTG**[A/C]**ATTGGGATTCGCACCTCATATGGTGCAGTCGGACCCAATGTCGATCGACACTCTAAGCAAGTTATGAACTGAAACTAACTTAGCATTCACCTCTGCCATC	A/C
2	CER445429	ATCATTTTTTGTAATGTAATGTTAAATTAAATTCAGAAAAATATAAAAAATGGATCAAAAATTTTTTTTTATATAATATTCATTGTTTTTAAACTCGACC**[T/C]**GGATGGTAAACCGGACTACCTTCTGAGTCACGGGTCATTGGTTTGCTTGGGTTCGATCATAGATTAATTGATTAAAATTTATTTAATATATTTTATATTT	T/C
2	PERL_319622_Ler	ACAAAGACATAAGTCATTTGCCCAATATCACATGTGGCAATTAAGACATAATATTTTAGTGAATACCTTGAGATATTCTTTGTTCAGAATATGTCGAGAG**[G/T]**GAACAACACCAGATGAGGCTGTTTGGCTAAGGCCATTCAGACAATCATCCTCTTCCATAACTTGTTTAGCCGCTTCCTCCACTTCGAGAACAAGAGACTC	G/T
2	PERL_323687_Ler	AATTTGTTTACCTACAAGTAGATGATTCTACACCTCTAAGGAATGACCTACGCCACCATATCTGAAACGCCTTGCTGAGATTAGGGCACTCAGTCCCATG**[C/T]**TTTGAGAACCACCTGAACCATTCCATGAACACTTGATACTGCTCTTTTACTGGTAGCGTTAAAAGCGTTTGCCCCATCCCTTCTTCTACTTCCCTCATGT	C/T
2	PERL_326605_Ler	AAGAAATTTAGAAAGAGCAGAAAGTTTAAACATAACCTACATTGGTTCCGTGAACGATATTGAGAACCCCATCAGGTAAACCAGCTTCCATTGCCAACTC**[G/C]**GCAAGTATTACAGAAGCACCTGGAGAAAGCAAGTAATATCAATAATCAACCAATCAATAGAAGGGACCTCTATAGCTATAGTGGAAAACAGTCAATCAAC	G/C
2	PERL_328856_Ler	TCCCTATTACAGAAGTCACATGGACACAATTGACAAGAACATAGGATACACTGTTTGGTACAAAGAGTTACAGAGATACAGATGCGAAAAAGAACACCGG**[T/C]**ATCAAGGGCCACATCAATTAAAAGAACGCTTCGCATTGATTCCTTTCCGGGGGAATAAATAGATGGACCATTTCGTTCTCTTAGGCACGTTCATCATTCG	T/C
2	PERL_330675_Ler	TTTTACCGCCGTTGATCTTCATTGATGAATTTGGGCATTCTTGACATTTAAAAAGTTTTCCGCCGTTAATCTAGAGTTTCCTCTTGGTCTGCAGTAGTGC**[G/A]**GTTTTGTCACCGATATAGTCGTTGCTGATCAGGTTTTAACGGAGGTGAAGGTTTAAAGCCGTGTTACGCGGTTGAAGACGTCGTGAAGTGCTTCGAGTTC	G/A
2	CER433367	GGATTCTGTTTCGTACAATATTTTGATCCGTGGTTGTAGTAACAATGGGGATTTGGAGATGGCTTTTGCTTACAGGGATGAGATGGTGAAACAAGGTATG**[G/A]**TGCCGACGTTTTATACTTACAACACGTTGATACATGGTTTGTTTATGGAGAATAAGATAGAAGCTGCTGAGATATTGATTAGGGAGATTAGAGAGAAGGG	G/A

2	PERL_336286_Ler	TCGGCTCCTCGGCTCGGTGTTTAAGAGTCTGAAACATGAGCTCAGGCTCATGCTGCATTGCTCTGAGGACATCTGCAGAGGATTGGCCCATCACAGCACG**[G/A]** GTCACTGCGAATCCGTGAGGTCCATCGCTCTGCAATACGCGAACCACGCTTGGTCCACCGAACAGATTGTGGACTCCACCGAGAGCTTCCTCATAAGCTC	G/A
2	CER432380	AGAACACGGTAGTGAGTATGTTCATCTCCTCCACCACCAACTGGATTTCATTTCATTAACATTTGGTTAGCCAGTTACAAGATTCTTGCGGGTTACATT**[G/T]**CAAA GGCGCATGCACATACCTGTACACTGTCAATCTCACACCGATCACCACATCGTAAGGGTGTACGATGAATAAGAAGATGATGGACTCGAAGACGGT	G/T
2	PERL_340933_Ler	TGGATAAAGGGATGGAGTTATATGAAATGATGAAATCAAGTGGTGTAAAGCTTGAGAGTAATGGTTATGCTACACTTGTGAGGTCTCTGGTTAAAAGTGG**[C/T]**AA GGTCGCAGAGGCTTATGAAGTGTTTGATTATGCAGTTGATAGCAAGAGTTTGTCAGATGCTTCTGCGTACTCTACACTTGAAACTACCTTGAAATGGT	C/T
2	PERL_343158_Ler	TGATCTGGACCAGATATGGTAAATAACTAATGCAAATTCCTTTGCAAGAAAAAGTTGAAGAACTTATTGTTGCCACACTCCCACAACTAGTTTCATAGCG**[C/T]**CAC CACCAGTAAACATGCATGTGTTTGATTCCTAAGGGGTTTTTAAGTCGACATTAAAAAGGCTAAATTGTTCAAATAGTTATCTTACAGATTGTTTTCT	C/T
2	PERL_345444_Ler	TTTCTTGGGCATAAGTCAAACAATTCTTATGGAGTGGGAACCGTAAGACTAATGTTGTAACGCCTCAGTCCCTTCTTAGTGAGGCCCACGTCTTCTCCGG**[T/A]**TT GTGAGTCCATCCTTATCCGACAATCCTTATCCGACAGTCCGTATACTATGTCTAAAGATTTTAAGGACTTGTTTATCCATCCTACAAGTTATCATATA	T/A
2	PERL_347732_Ler	AAAAATGAGCAAAACTAGTGGTTCACGTTGCGGTACAACACATCATTTGCATGCAAACACATTTCTCTTCTTCGTAGCTCTCTTTAGCATCTGTCAGGCT**[C/T]**AAT GTCGTCGATTCATGCGAAGTCTAAAAAGAATTTAAGAATTTTATCTATCTCAACCCATAAACTTTAAAGATCCGTTTGGTTATCTATCTTTGTATTG	C/T
2	PERL_349483_Ler	TCTACCCAAGAACTACACAACCTACTCGGTATTCATCCCCGAGGATGATGTTCGGAAGAGAAACATAAGCATCGAGGAGCGACTCCTCCAGATTCCAGCC**[A/G]**A GCAGGTCAAGATAATGAGAGAGAATGTCATCAACCTCATCCCAAGGCTGATCTACGCAGACCCGAGATCAGAACTGGAGACGCAGAAAGATGCATTTGA	A/G
2	PERL_351339_Ler	GGTTCATCATTTGTGCAGTTAAACATTCCTTTGATAATCTCCTCTTTTACCAGAAAACCAAAAAGGTTTCCAAGGTGGAAGTAGGAACTAGGTCTACCGA**[G/T]**GCA TCACTCGATGAATGGCTCGAGGTTCGCTTCTCTCTTTGTACTCTTCCTCCTCTTCCTCCTCTTCAGTGTGATTGAATAATCTGATTGAAGGGTATGT	G/T
2	PERL_353122_Ler	CTCTAAAAGTACAGAAGCAAATCTATGAATTTTACTACACCGGCAAGTGTTTCATCTTGGCTCAGGGCATTAAGCCAAGCTATACTCTTTGTCGATTCAC**[T/C]**AGTT CCGGCAAAGTAGTCTAAACCACTATGCTCATAACAATGATTGTTGCTCACGGACATACATAACACAACCAATAACGATGAATCAGAAGCAGACCTG	T/C
2	PERL_354658_Ler	TCTTTGAGAGTATTATCAGTATCAATCAATCTAATGTATTATTATTTTTAATTAATATTATCAACCAGTATCTATTTGTATTTGATTGCCTTTCACGCCT**[G/A]**CACATA AAATTCAGAGTCGTCGTATATATGTATAAAAATATAATCGTGTTTAGCCATTAGCACATGAGCAAGTCCAAATCTCCCTCTCTCTCTCTCTATC	G/A
2	PERL_356299_Ler	CAGCACATTCTCCAGAAACTTCTGCTTGATAGAGCAACCATCGGTGGTACGGTTACAATTCTCCATCAAGAAACTGTTCTCCGACGTTGGTGCGAAATTC**[T/A]**CG CCGAAAAGCGGATTCATATCTTCAATCTTCCGAGTACAATTGTGAGAGAGACCGACAAATATACTGTTTTCCGTCACGTTTTGAACGGAAAAGTCTCC	T/A
2	PERL_357977_Ler	TTTAAAATTCATATCAGTCTTAACAAATCCAGGACAAACCGAGTTAACGCGAATCTCGGGATGTTTCTTCGCTAAGATCCTCGTGTAAGCATTCAAACCG**[G/A]**CTT TCGAAACAACGTAAGCCGACATGACTTTAGCCCAATATTTTGTCTTAGCCGTATCTTCTTTCAGATCATTGAGAAGTTGGTTGATCACTTGGTCAAT	G/A
2	CER429760	GCATTAAACCAACATCGTTAGATGTAGCTAGACATGCCACATCAGTAGAATCATTTGGGAATCACCGACCGCCGATATCGCCGTGGCACTCACCGGTGCC**[G/A]**T ACCTATTCGGTGGTCTTGCGGCGATGTTAGGCCTCATAGCCTTCGCTTTACTTATCCTAGCCTGCTCTTACTGGCGCTTGTCAACCTCCGGTGATGACT	G/A
2	PERL_362547_Ler	TTTTTAGAGAAATGATTATTGGTAAGTAAATAAATTAATACTCTTATTTTGGGCCTTTCGGGACTTATTTTTTTACTATTCGAGGGTCCTAAGTCATTTG**[G/A]**CTGCC GACCTGCTAAATATGGGAAAGGATCCTCCCTTCTTATTTCTCATCATACTAATCTCATAAGATATAAAATTTACTCACTCAATGATTTGTGTCCA	G/A
2	PERL_364614_Ler	ACTGCTTGAAACAATTGGAGTGACGAACACTCGTATCGCACATGTAAGGATGACATCCACTAGAAGAAGAAGAGCATGTCAAAAGAACAGCATTGTGAGG**[T/C]**G GTTCCATGCAGATTACACACCGCACATCCTCCCAACCTGTCTCAATAGGCGACTCTGACGCCTTGTGTTTGTCGGTCCTCGAAGACCGAAGCGGGTATG	T/C
2	CER427000	TGTCTTACCGGCCTCATTGGGTCACGTCGCTGTTCCGGTTCCTGGACCGGTCCGATCACAAAGCTCTTCTTCGTCTCCTTCACCGTCCCCATTGGTTGAT**[C/T]**CA CCCGTTAATAATGCTCCACAGTATCAGATGGGACCAACACCAGCTTCACATAGCGCAGCTTCAGCAGACTTTATCTTTACTTTCTCTTTTGATCTTAC	C/T
2	PERL_369404_Ler	GCAAATAGAATAAAGTAAATGAGACATAAGCAATCAACCAATAAGAGAATGTAAACACCCTAAGAAACTGTTAGGCTCTAAGACTTACCTTGTGCTCTTC**[A/G]**CGA TCTTGCATTCGTGGAAGAGCCGCTTCATGTTTAATGTCATCATAAACTTCATCGTATGCAAAAGCAGAAGGATCCTCCTCTATAGCTTTCTTATGCT	A/G
2	PERL_371102_Ler	ATTTGATGAACTACTGATGACGTGTCGGTTTCTTGAAGATACGACGCCGTGTCTTTTGGGTTAATTGCGGCGACTCCGGGAATGTAGTCGATTAGATCGC**[T/C]**GC GGGTTTCTGTGATAAGAACATAAAATTTATGGGGAAGATTTAGATAATTAAGGAAATTGAAGGATGCGTTCTGTAAATTAGTTAAAGGAATAAATAAA	T/C
2	PERL_372171_Ler	CTTCTTCCGTTAGACCAGAGTTTTGTTGCATCTATAGAGTACCAGACCGGTTACGCAAAGTGAACCCCGAGGCTTACACTCCTCAAATGCTGCTAATCGG**[A/C]**CC TCTTCACCATTCCAAGAAAGCTGAAGCCCTCAAGCGCTACAAAACCGATTTAAGGTAACAAGTAACAACATAGTATTTTAACTAGTATTATTTTTGTT	A/C
2	PERL_373965_Ler	GGTAAGGCTGATGAAGCTTAGGGAGAATGATTAATGGTCCGTACACAGTAGCTCTCATCCACTGAATATGAGCATGCCACCACAAGGTTCCTCTTTGTCC**[A/G]**GT CACCGTGAAGTTGTAAACATAACTTTGTCCCATCCGAATTGGACATTGTGTCACATAGGACGGTCCATCCGCCCAACCACTCCTTAGCTGCCGGATAC	A/G
2	PERL_375688_Ler	TTTTTGTCAACAAAATATTTTTGGACCCTTGACCTATAATCTCATGCGGTCCGGAACTAGTCGGGAAAAACCGTTTACATAACATAGTGACAACCCATGC**[T/C]**GCC TCAACTATGCTTGCAATGCTTCGAAGTTCGGACATCCAGATATTGCTCGATTTGTATTGCTGCCACTTTTACTACCATTCGGATCTCTATCTTGGTG	T/C
2	CER442035	ATATCTGTAATGGTTCTAATAAGAATATGGTATTTGCATGTTATCACGTATGTAGCAAATCAAAAGAATCAAAAACCACAAGCTGTTGTAAAGCCTGAGG**[C/T]**GCA GAAAGAAGCTCTTCCCATCGAAGTTCCTCCCTTGTCTGTGGATGAGGTTAAAGAAAAGACTGACAATTTTGGATCTAAGTCTCTGATTGGTGAGGGT	C/T
2	PERL_378201_Ler	TTCCGACGAAAGCAGATCGTCCACCGAGACGAGAAACAGCGATTGCGACGTTAGCGGGAGCACCGCCTGGAGCTTTGAGGAATCCAGGAGCTTCGGCGAG**[A/T]**GAGACGCCTGATTCGGTAGGGACGAAATCGATTAGCATCTCGCCGAAGCTAACGATCAGTCCTTTATCACCGTTGGATGCCATTTAGATCAGAGATCTGT	A/T
2	PERL_379592_Ler	TTTTTGTTTTCATCAGTACACAAGTCCCTGGTGAGTGGACAATATACCCCTGGATACACAGCAACTGCAATTCGCTCTCGGACAATGATCAGCTACGCCC**[T/A]**ATT GCGATTCCTGATATTCATCCTGCAAGGTATGCCTTATCGAACAACAGTAGAACTGGCCTATGGTTTTTGGAATGTGTTGGTAGTTTTAGGTATAAAG	T/A
2	PERL_381438_Ler	CCTATGTTAAGTTCAAATTCATGTTTTGTATTTAATCTTATCTACGGTTGTAACCGTTTATTTAATCTATCTCATTTATGTATCATTCCGGGACTTTTCG**[G/C]**CATCGA GTACCCGGTTTCTTTCGGTTGTAATCGCATTCGAAGATTAATATAATATTGTTGACAAAAAAAGGGTTTCAAATTCATGACTTCGAAGCTATAT	G/C

2	PERL_383347_Ler	CGGAGGTAACCCCTCGCCATTGGGAAAGACAAAAGGAGATTTGCATGGTAGCGATCTTGACCGTCGATGTTCTATCTTAGAGATCTAATGGTTAGGAATT**[T/G]**AT<br>GGCGGTACCACCCGGTCAAAGTAATGCGTACCGTCGATATTTGACACGTGTCGGTAATTTAGCTCCGGTTTGACAGATAGACGATACGAGAGATTACC	T/G
2	PERL_384706_Ler	CTTTCACCCCAAACATTAAACTTTGAGATGTCTTACTCTCTAAATTCTGACTTTAGCTTTGATTATTTTGTCTTAGCGTCTACATCTGTCTGCGAAATTC**[T/C]**ACACA<br>ACAGCTGCGTAATTTCTCCATGACACTATTTGCGTATGTTTCTCAATCTTAGTCTCATTCTAACCAAGCTTAGTGTTTTATTCTGTACACAACTC	T/C
2	CER427509	CTCAGTATCTGGACATGGTACTTCAAATAATAGTTCATCTAATGTGAGCAATTTCGACCAGCCTGTGACTCAGCAAAGAGTCTTTCATTCTGAGCGAACT**[G/C]**TAC<br>GAGATCCAACAGAAACTAACCGTTTGTCTAAATCTGATGATTCCCTTGCTTCTCAATTTGTAATGGCTCAAACAACATCAGATGCTTTCCTGCCTAT	G/C
2	PERL_387436_Ler	TGTTCAAAGGAGCAGTCCCGAGGTTCTTCTGGGTGGCTCCTCTAGGTGCTATGAACTTTGCTGGGTACGAACTAGCCAAGAAAGCTATGCAGAAGAACGA**[A/G]**G<br>ATGCAGTGCTAGCGGATCAGCTTGGTCAGAAGAAGCTTTGCTAATGGGATTTTACTTTGCTTTGCTGCTACGAAGTTTTTTGGGGGCAGCTTAAGCCGG	A/G
2	PERL_388688_Ler	TTTATCTCTATAATACATTTGCTTATCGGTTTAGTTATTGGTCTTAATTCTGACTGATCTTATTGTGCATGCAGGACAATACAGGTGTGTTCCCGTTGAG**[T/C]**TATGA<br>CCCGTCAGACACAAAAGATAATTTACTTGGTACGCAAACAGTTTTTGACTTGTGATGTTTATCTGTTATATTTAGACATGTTTTTTTTTGTTTAT	T/C
2	PERL_390343_Ler	TAGTGAAACACTGAACAACGGAGTGATTTCGAAGGCTTCTTCTCACCAGAAGAGCATAAGCATCGATAATAAAAAGAGACAAGCTCCACAGGCTTTGCAG**[G/A]**CT<br>AACCGCAAGAACCAAGCAACTATCACAGTAACAGATAAGAGCTTGTCAGATTCATATAGTCTAAAACCAAATTGCTCAAACTAAATCCAAATTCTGAT	G/A
2	PERL_391711_Ler	TTGCTAATATCAGAAAAAAAAAAAAAAAAACTGACATATTTGTAACAACAACTACAAAATGAAAATTTTATCTTTCTAGGTTTAATGTTTCTTACCGGGTG**[A/G]**AAATT<br>CTATCCCTCTGAGTAACATCAGTAAAACTACTCTACGAATACCTGAACATACATATATAAATCAGAGAATATTTTCACTAAAAACCGGTCTTTTC	A/G
2	PERL_393403_Ler	TGCAAACCTAGTTTTTAAGCAGTTCAACAAGAAATTTTGGGCTCTTCTTTTTTCGCCTAAACAGTTGTTCAAATTCTCTAACTCGATCATTGATTACTGC**[G/T]**AAAAC<br>TCGACCACTCATTGTAAGTTGTTGAGAATTTATGATGTTTTTCTTTGCACTTGTTCGAGTAAATAGAATGTGAATGTAGTAGCTAGCTGTGCTTT	G/T
2	PERL_394981_Ler	TGGCCCACGCAGATGGATTTCCTCTTTCAATAAAAAGTGCCACAATAGTGCTTTCAATAGACCCCAAAAAACATATGTAAGCTGTCAAGGATAGCTCTAC**[A/C]**GG<br>GTACGACTTAAGCGTGATCGCCTGCATAAACAATCATTTTAGTGGTTTAGTACTATATATAACCGTTTCTAATATATTGAACAATATATAAATATTGG	A/C
2	PERL_396668_Ler	TCGATAAATGGGAAACTCATGACGCCGGAGCAGAAGTTAGATCCGAGAACTTTTGCTGATTGTTATCATTGGTGTCTTCCGGGATTGCCTGATTCATGGA**[A/G]**CG<br>AGTTGCTCTCTCTGTATATCATCTACAAAATTTAAATGTTTTTTCTGATTGAATGTGAGAATTTGCAAACACCAGATTCCTTCTTCATCGGGAACGTC	A/G
2	PERL_398212_Ler	AAGAGCATGAGCAGCAGCAACAAAAGGTAGATCAGAGTGACATTTACATATCGTAAGGCACGCGTTTGTACAGATCCGAAGGCAACCGAGGCGTTGCAAC**[C/T]**G<br>CTTGCACAGGGGTACGCATGTAAGTAACGAGTCCAGGGTTTTCAGACATGTCAATCTCCTCCGGTTTAGTCCCTTGAGGAGGTGTCCAAACAAAATGGT	C/T
2	PERL_400707_Ler	TTTCAAGACTCGGATTTTATCTATATTTAGAATTTTATAGAAGGTATGGCTTATGAACTATTTAATGATCGGAAGAATTTGAGACTCAATACCGAAGTTC**[G/A]**AAGA<br>CGAGTGGACAAAACAGCAAAGATGGTTCGGATGTATTGCTTGATGAACTCACGAGGGTAATGATCAGAGTACATGAAGCTGCAATGACTCATGCCC	G/A
2	PERL_402952_Ler	CGTCGCTTCCATCGCAGCAATCTGAGAAACAGACAATGTCATGCTGCTTATCCAATCCAACAAAAACATGTAGATTACTAGCATACAGCAAGAGACAGCT**[G/T]**AA<br>ACGAGAATCATTTAGTCGAATTCAGAACATTAGTAACTACTGACCACAGATTCGATCATTCACACGAGAAGAGTAAACGAACTTTGGAGAACTTCCAA	G/T
2	CER425424	TATGCCAGGGATGGTGGGGAGACCCCACAAGTTACCGCTCCAGGAGCTGCTTCTCCTCCTCCCCCACAAGTTGCTGCTCCAGAGGCTGCTTCTCCTCCAA**[C/T]**T<br>GGAACCAACACAGCCAATACAACTGCTACTTTTGTTAATGAATCACCTTCTCAGAAAGTTGAGACTTTCGATGAATTTGATCCACGCAGTGCGTTTTCA	C/T
2	PERL_407406_Ler	GAAATCTTGTACAACGGTGATGTTGTTGGTGAAATGAGGATTAATGGACAACGAGTGGCTTCATTTGGTGACTTAAGAACAGAGGTTAGTGCAGAAGTGG**[C/G]**TT<br>ACAGAGGGAATCAGAAATCACCTGTTTGGTTGAAGAATGATATAGAGAGAAGGCTAATAATCCTCGAGACCAGAGCTAAACTAAGAGGTGAAGTGCAC	C/G
2	PERL_409455_Ler	GGTGATGGAGGAGGCATCAAGTCTAAGGGAAAAAAGAAAGGAGGCAAAAATAAGAATAAAGGAAAGAAGAAAGGAGGTGGCAAGAAGAGAGGCAAGTAAT**[C/T]**<br>GCGGTAGCTCTGTAACAAGTTTTGAAGCGTTTCAAGTATCATTGTATACTTCACAAGGATTACTCTTGGAATTCAACATTACAGTGAAAGAGCTATGAGT	C/T
2	PERL_410860_Ler	AGATGATTTTGTTTTATTTTTATCACAAGGGTTTGAGCAATTTAAACAAACAGAGGAAAGTATTACAATGGCTAATTAATGGTCTGTTTCTTTACGGGAA**[T/C]**GGCA<br>TATAACGTGTTGGTCCTGTGTAATGTGATACCAAAGGTCTCGTCCATATCCAAGTCCTCTGAAACGACACCGTTAGGAAGCTTCCAGTCAAAGGAA	T/C
2	PERL_412300_Ler	GTTAAAGCTATTTAATAACCCAAAAAGACTTCGAAGAATATATGAGATTAGCCCCCCTTTTTTCATTTTCTTAAAAAGATTTGTATAAGATCTGCAGTGC**[G/A]**AAGG<br>TGAGATACGTCTATATATTCTATAAGATGAATAGCTGCTCTAGCTACTTTTTTTGTGTGGTACATGTTATGATAAATTGAAAACGTTTAATCTATG	G/A
2	PERL_414078_Ler	TTCTTTTTATCTACTGGGCCTTTTTAGTTATTGTGCAGATTATCTGGAAACTTTTTCTTTTCATTTACTGGGCCTTTTTAACTAGCTAGTACGGCGGAAT**[A/C]**GTGAA<br>AACCGCACAAGCCAATTTATCTAAGATTTTGGATTTTGCCACCCACCCAACTTATCGTGAAAACCGCACAAGCCAATATACACACTCAGCCAACT	A/C
2	CER448159	TCCCCAGCGCCGCAGCGAGATCGGAGAGCGGTCTCATTTGGTCAGCCTCGTCCGCCTTGACGTCTTGAAGAGCCAGCACGTCTGCGTCTAGCTCACTCAG**[C/A]**<br>ACTTCCAGAGCCGTTCTGGTGCTCCGGAGACCGATCTCGCCGGGCCTGAGGGGAGAGTGCTGTGGATCCTCCCGGAAGCTGAGCTGGCGGCTGATCTCGT	C/A
2	*PERL_416019_Ler	AACGCAAGCCTCAGATGGAGACATTCCATCATGAGATTTCTCCACGGGAGATGGTGCTACATTGACCATGTCCCCTGAGAGCTTAGCAGACACTTCAAA**[G/A]**AAT<br>TTAGATTCTTCAACTGCAAAAGTATCATGACTAGAAATGATATCAGAAGGGCTTGTCTGTGCTGTTGCACTGACCACAGTGTCCTTGGGTGATCTT	G/A
3	*PERL_416091_Ler	GAAACTTCACAAATTTTTTGAGTAGATCAATTCGCACAATGTTACTCACCATCCGTAGAGTTGTACCTGTTCCTGTGATTGCTTACTTGTTCTGTTATC**[G/C]**GTGAG<br>CCTAACATGAACCTGTGTTCAACTCTTTGTACTGATCATACTACTTGTCATTTTGTTCTCTTGTGTTTGACTAATGATAGAGAAGGAGAATGGG	G/C
3	PERL_416091_Ler	TCCTAGTCTAGTAATATTGGTTGAGAGACCTGATAAGTGAACATTGTAGTTGATACCAATTTGGACTTTTGACATAATGACAACATACTTTGGTAATTAT**[C/T]**GTCTT<br>CGGCCAAGATCATTTTAAAATTGAATCAACGTTTTAGGAAACGTGATAAGTATACTTGAAAGGCTGGCTTTAAGCGAACAAATCCAAAAAAAATA	C/T
3	PERL_420280_Ler	GATACAAGCAAACAGCGGCACAGTCATCGACTTTGGAAGTCGGGTATTTGTATCTCCAAGCTCTGACCGCAGACTCGACTAAAGCTCTTGCTGCAGAGGA**[G/A]**C<br>GCGATGGAGCTGAAGCTACAATCGCCACTACATCTTCATTTGAGAGAACATCCCAAATCTATCACAATAGAAAGAAATGTGAGCAGCAAAAATTGCAAA	G/A
3	PERL_422425_Ler	AACCTGACAGCTCTTTTGAATTTTAATTATGTTGCTTGTCAACTAATTCAAATGGATCTTGATTCCAAAACTAATGTTACTAAGAAAATATTTACGCTAG**[G/C]**CCAAT<br>TTGGTTGTGGATTTACGATATTTTGGGCTGGGCCAACAGAAAAGAGCATGTGGCCATATTTGATCAAGTCATTGTAACTTTCATTAGCCGTATGT	G/C

3	CER473550	GGATCCTCTGTGTAGGATTATCGTTCACTTACCAGCTCTTATATCTGCTGGACGCTACTGGATTTTATTCTCTTGGGCTACAAGCTCTTGGGATTCAAGT**[T/A]**TGTAGAGCTACAGGGCAAGAACTGGTATTAACTTCCTCCTTTCTGGTTGAAATTTCGTGTCACCATTATCTTACTATGCACCCCATAGAATTAGGGCGCT	T/A
3	CER468485	AGTTAAACCGTTAGACCAAAACGACGTTTTGGTACCAAGAAATAAAATCGCAGTTTGTTTTTGGTTAAAGCTTGATGAAACACGAGACTTTGGTTTCTGT**[A/G]**TTGTACAGAGTTTGTTACTTTGTTTCAACGTCCATATAGTTTTTTTATTTGGAAAAAAATAATGATTTATTCCAAAAATAAAATTGTAAAAACACTAAAA	A/G
3	PERL_429038_Ler	TTGAGGCTTTTCTTGGTTCATATACTCTTACTTTTACTGCCAATTATTACCCAAAATATTCCCTTTAATTTAATTAAATGGTTGTATGTCTAGTGATCTC**[T/C]**TAGTGGTGCCCTGCATAACTCATGATTTGTGAATGTGTCGGTCTACAAGATTCGATCACCACAAATAAAATAACAATCACTCACAATACATAGAAAAAAG	T/C
3	PERL_431508_Ler	ACAAAATTATTATAAATAAAAAGTGACAAGAAATGAAAAGAAATAAAGCTGCATCGCCGTTTCAAATCCAATATTGCAAACGTTATCCAAGTCTGTCGGC**[T/C]**GAAATACCGGCAACTAACCATACTCTGATATGTTCCTCACTCATAAATGCTAACATGCTTAAGTGTCATAATTTTTGTTTTGTTTTTGGATTTTACTGAT	T/C
3	PERL_433766_Ler	TATAACTTGTACTGAGAGTGCAAAAGAGAATAATTGCATGAAGAACCTAATGGAGTTGGATTAGATGGATCATGAAACTTGGGTTTTATTGTTTCTAGGC**[C/T]**CACGTTTTCTACGCAATTAGTTAGATTTTCACATGCATATTGATAACAAGTGATTATCATCTCATATATTAACGTAACACAGAGATGATGGTTTGGAGAA	C/T
3	PERL_435300_Ler	CTGTTAATCTGCAGTGTGTCAGTGCAGAGGTAGAGGTTCCTGGTAATGGTCCTTACAGTCCGATTTCCTTTGTGCCTGGTCTCCCGGTGGCTATACCGTG**[T/C]**GAGATCACACTGCTTAATGTGCCAAGAGATACCTGTTTATGGCTGAGAATATCAAGAAACGATGAAACTTGCCAATTTGTGTACTTGGATCCAAACCTCT	T/C
3	PERL_437189_Ler	GTATTCAACAACATCAATCAATACTTACTAGTTTTCTGAATAATTTTTAAAAAATCCTAGAATCAAAAACCTCATGATTTTATAAAACAGAGTATCTGCC**[T/G]**GATTGTGCTCCTCCAGGAAGGGAGAAGGTTTGGTTAGTTATGTCTTCAAGAACACGCTTGAGTTCAGCTTTCGCTGTCTTAACAGATATTTCGGTAGGCC	T/G
3	PERL_439306_Ler	ATATGGAAGTCAATAAGCGGTAACAGAGGATGCTACTTTAGAATAACAGAACGAACTATCCAATAAAAAGGAAGAGCTAACAGATATTAGAAACCTGTGA**[A/G]**TAGTTTTGAATGGCCTTGTTGAAACCACCATGATAAGCATGGACAACTTCATCAACAACCTCCTCGACAACATCACTTTGTTCCTTTAATATATGAATGT	A/G
3	PERL_442052_Ler	TATTATAATATAGAGATTATAGATAATTGTATAGATATTGTACGTTAAAAGAAATATGTATATATTACATGGAACATCATAGGGAATTGAATGTTAGTGG**[A/G]**CATTACGATGTGAGACCGCCTTCAACTTTTTTGACTACGATAAGAACACAATTCTCGCTGTCTGAGAAGTCTATATACAAATGAAACAACTCCAATGTTA	A/G
3	CER477518	TATTCGGACAAACAGCTCAAGTTTCTTGAGGCTTGCTCTGAAAAGCCGAGTTCAAAATGCGGAAATGAGGTTTTCAAGAACATGTTAAATGAGACGATGC**[T/C]**AATTACAGAGGAATGTTGTCGTGATATATTGAAGATGGGCAAAGATTGCCATCTAGGATTGGTTAAACTCATATTTGCCACATATGAGTATAAAAATATT	T/C
3	CER437449	CAGACGGTCCTTTGATGCTCTACGTCTCTAAGATGATTCCCGCATCTGACAAGGGCAGGTTCTTTGCCTTCGGTCGTGTCTTTTCTGGTACGGTTTCTAC**[T/A]**GGTATGAAAGTCAGGATCATGGGTCCTAACTATGTCCCTGGTGAAAAGAAAGATCTGTATGTCAAAAGTGTGCAGAGGACTGTCATTTGGATGGGTAAGA	T/A
3	PERL_448757_Ler	ACCTGAGGATTAATGGACGGTGTTGGTTTTCACAAATCATATGGGCTAAGATTTATTTATAAATGGGCTTACAAAAGCACAATGTTATGTTATCCTCCAA**[A/C]**TTCGGGCCTCTTAACACTTGTACACAACTACACATGATATGAATACAAGCATATCTTCGTTCACATTTATTGCATCATTGCATATGTGTACACAAATAGT	A/C
3	PERL_450708_Ler	ATCTAGCCGATAAGAAGATAACTTAGAGAAGTGTGACATGTTTTGCTTCTATCTTACATGATAGTTTGATGCCGTCAATTTGATTTATGTGATGGGCCTG**[T/G]**ACCTGGGCCAATTCTTGATTAGGCCTGCATTAAAATATTCTGCCAACGGCTAGAAACTTTTTCTCTTCTTCTATAAATTTACTTTTTTTGGCCTAATCCC	T/G
3	PERL_452986_Ler	ACGTGTCTTTCATCTATCTCACTCATACCGGACGGACCGGTCTTCGCGTACGTACGCAATAACAGCTTCCAAGTAGTTTCATTCCCAACCCAAACCGTGT**[G/A]**ATTTTGCGTTCTGTCTGAACATCTTATTCCCTGACATACCGAGAACACCCCTACTTCCTTTTCGACGTTTCTTGGAGTAAAGTAGCAATGTCGGTGTAAA	G/A
3	PERL_454858_Ler	GAGAATCAAACTTTCTTTTAGTTAAAAATGAAATACAAAACCAATAAGTAACCATCAAAACATCGCAAGCTTCACCAAATTCTTCAAACTCTAACCCGTG**[G/A]**CGTAAATAATACAATAACTCTCAAATAGAATACACAAATAGACTAAATGATTAATCTCCCTAATCAAAATCTACGCAACTATCTTTAAGCTCTCCATGCA	G/A
3	PERL_457971_Ler	CACTTTGGGCTCCACGACTGGAGTAAAGTAGTTAATGCGGTATGAGACGAACCAGTTTCTGTCAAGGCTAATTCGGCTGCGTTCTTCATAGCCATGGTTC**[G/T]**CTCCCTAACCGGACACTCCCCAATTATCTCTTGGACTCGTTTCTCCACCTCTGTAGAGCTCACGAAACCCGTCTCTGATTCATTCATCGAAATCGCAATC	G/T
3	PERL_460787_Ler	TAGGGTTTTACTTCCACGCTTTCAATCTTTTATATTGTGACGGCTCAGTCAAATAAGTTACTCGCTCTGTTTTTTCGGGCCCGAGAAGACTCTGGGCCTC**[G/A]**ACCCGTAACTTCCTAGATAACCCAACTCAAAACTCTCCTTTGCAAATTATCATTCATCCGCGCCAACCACAAGTATAACATGATGAAAAGGAACGTTTTT	G/A
3	CER439712	TCCTCTGCTTCCGGTGGTTACAAATTTGAAGACAAAGAAACCCACCCTAACTGAATTGGGCCAATCATGGGCTTTAGAAATCTAATAGGCTTGTAATGTG**[G/C]**CCGGTGAAAGAGATAAGAGTGATATATTATAGGGATGAATTGTGAAGATTGAACCTCAATAAGACCAATCGGAAGATCACGAAGGTAAGTGTAATCAAAA	G/C
3	PERL_466437_Ler	ACGTGTTATAAATTTGATCACTTAGTTGCAACCTATGGAAAGTTAGTTGTTAGTATGTCAGACAGTAATCAACTGATCAGAAACCAGTTTGAAAGGCTCA**[C/T]**GAAGATTGGTCTGCAATAGCTGTTATAATAGATATCACTTGCGAAAAGAGACTGACATTTTGGTTTAAAGCATGTTTAGATCTTATAGGTTGATCTATTG	C/T
3	PERL_468933_Ler	TACGTATAACGCCTGAATAAATCTTTCAGACCTGCGTCGACGGTTCGCTGCCGCCGAAATTTCCGATCAACCTTAATAAGGAATATGACGTCTCTGTCGG**[T/C]**AAATGATCGGAAGGATTTTGTAGATGGGCAGATGAAGTACTGGCGTCAAATAGCCGAATCCGATGTACGTTCTTCTTTCATTTTTATGATCTAATTTCGT	T/C
3	PERL_471183_Ler	TCGAAATTATTATTAGTTTCTTCTCCTTCCTTTGGGACCCTACAAGAGACGAGACGCGCTTATCGGCATCGTCGTCGTCTCCCGTGTTAAAAGTAAATGC**[C/T]**GTGTTGAGGATACGCATTAATGTGGAGAAACAAACATTTTTGTTCTTATAAAAACTGAATTATGTCTCTCCATTAAACCCCAATCTCAGAACACAAAACG	C/T
3	PERL_473449_Ler	TCCTTCTCCGATTTCGTTCTCACGCCTTCGCATTCCTTTTTTTAATTTCGTTTCGAAACGCGTTTCAAGGTTACGTCGATCTCAAAGTAACATCGCCTTC**[A/G]**GAGAGAAATTGATAAACCTTGCTCCAATTCCATTGTTATTCATGGCTCTTAGAAACTTTACCTAGATTACTCGGATTCAATCGTCAAGTTTATATTGGAT	A/G
3	(1)<br>PERL_476047_Ler	GGGCTCACACGATTGGCGTCTCACATTGTTCTTCCATGAATACGCGTCTCTACAACTTCTCGACTACAGTAAAACAAGATCCATCTCTCGATAGCCAGTA**[T/C]**GCAGCAAATCTAAAGGCTAACAAATGTAAGAGCCTTAACGACAATAGCACCATCCTCGAGATGGATCCCGGTAGTAGCAGAAGCTTCGATCTCAGTTATT	T/C
3	PERL_478227_Ler	AATTAAACCCTGTTCTAGCACAATACTACAACATTAAATGTTGAAGGTGGGGATAGATTACACCAAAACATTCGTTCTATTGTCGAAAGTGTCAACGGTT[A/T]GGAGAGTTCTTAAGATCGCATTAGCTATGAACTTTTTATTGTTTTTGTCAGGAAAAAAAGAAAACTAGCAAATGTTAATTACATTTAAGAGAGATATAAT	A/T
3	CER434254	TGTGTTAAATATATATATTTTTTTTGCATATTTATTGTTGCATATTGTGTTGCATATTTAGTAATGGTTACATTCCCTGTTATCGGAGATCCAAGATAAT**[A/G]**CGGCTCTTGTGGCATGGACTACTACTCCATGGATTCTTCCAAGTAATCTTGCTTTGTGTGTCAATGCAAAGTTTGTCTATCTTAAGGTTCGTCAACAACA	A/G

3	PERL_484378_Ler	AGTGGATTGTTGGTCGAAGAGATGAGAGGCGAATGAAACAGAACCTAAAGTAGTAGCGCCACTAAAAACCGCCATTGTTGCGCGGTAGAGCAAAAAAGCC**[A/G]**CGGAGATTGAAGGTAATGATGGTTAATGGATTTTGCCATTTTGGATAAGAAAGTGAGAGACTGGAGATGAAGGTGACGTGGCATTTGTCAGCTCACAGGT	A/G
3	CER435667	AATGCACAATCATTTCATAACTAGTTTTTTTCTGACCTGAACCATCAGCTGCCGTGAACGCCTGAGACTTTCAGTTATGCTTTCAGCATCGGATGTCACG**[C/T]**CAGCATTTGCTCTATAAACAACCACAAATGTTAGATTCACTGGAAACTAACACTGAACTCTTTTTTTAGTGCATGATGCATTTACCAATAAACAAAAGCA	C/T
3	PERL_490837_Ler	ATGAAAACAGCTTTGAAAGAAAGAATGGATTTTGATATAAGAAATGTGAGAGAGAAGATTGGTATAAATAAAGATAGAATGTGGTTTTGCATAGTGGAAG**[C/A]**AGTGAGAGCTAAGTGAAGTCGTCATTGAAGTGAAATCGACGTTAGATGACTTTTCTAATGTATATTTTGGACTGGAATCACATCACTTTGAAATTATACA	C/A
3	PERL_495988_Ler	TCATTTTTCTTTCTGTAGTTTAGGGACCGGCCAAAAGTACATCAACTTAGACCCAAGCTAAGACCCAAAAATTTGGACCATTTTCTAACAACTTGCCGTC**[C/A]**ATGGAAACCGAATCAAAAATAGCATCATTCGTTGGTGACCCATTTTTGGGATATTTAGCATTATCCCATTCACCATTTATCAGCCATCAATCATTCATCC	C/A
3	PERL_499681_Ler	AGCACCATTGACAAACCCTAGAAGGTTTTGTCCAGAGAGAAAGGACTCAAACTGAGTCTTCCAGAGAAGATAGTTTCTCTCAGTAAGTTGAACCGTAACG**[T/C]**AGTTGGAAATGTGAATGACAGGATGCGAGTAGAGCTGGATTTGAGAATCCATGGCTCTGATACCATGAAAGTAAAGATGCTCAAAGGCTAAATATGAAGA	T/C
3	PERL_502764_Ler	AGAGAAGCCAACACCATATGATCGATCGCAAGCATCGCATTCAATACCATCACGATGGTAAATTAGGGATACACGATGCTCGTGTCCTTGTAACGTGAGA**[C/T]**TCATAGCTGTTGCGGTTAGAGAGGATCAAATTAATGTTTTGGGGTAATAGCACTTTGGTTTCGTGTAATAATAGATCAATATAGTTGTTCATCTATATAC	C/T
3	PERL_506778_Ler	TTCTTATATTTATATATTATTTATTTATTTATAATTTTACACAAATTCATGTAATTCTTTGTTGATCTAGCTTAGTTTAAATCTTCATAGGCATCAAAGG**[C/T]**GGCACTTGAGGAGAGCAATAAGCATAATTATACCGGTCCAAATTTTCTAGGCTAAATTTAAGGGTCCAAACATTTGTTTTAGTATAAAGACAAGGCCATT	C/T
3	(1) PERL_515012_Ler	GAGTACCAACCATTTCCTGAAACCTGATGAGGGATGCGTAAGCACCGGATTTGGCAATGAGTTCTTCATGTGTTCCGGTTTCAACAACTTGGCCTTGCTG**[T/A]**ATCACGGCAATGGAATCAACATTTCTGATGGTGCAGAGACGATGAGCAACAACAACAGTGGTCCTCCCCACCATGACTCTGTCTAAAGCTTCCTGAACAA	T/A
3	PERL_520525_Ler	GAATCATCCAATATAAAATAGAAAAATAAAACGTGATCTAAGTAGGCTTAGCTAAATAGTGCACGTATGGTAAATGAGGACTCTTGTCAGCTTTGAACGA**[C/T]**CGAGTAAGGAGTGAAAGGCCTTTGAAAACCAGAGAAAAGCAGGGGAGTACTCTATATTGTGCGTTCTATTATTGCGTCTTATTTCCGAGGGAGTAGTCGT	C/T
3	PERL_524537_Ler	TTTTCTGTTACTCGCCGGCTAATGTCTCCGGGAGGTTTTTGTTACTCGCCGGCTAATGTCTCCGGAAATGTTCATGTTCTTGCCGGCTTTATTGTCACAG**[T/C]**GGAGGAATCGATCACAATCTTTTGTAGCCTGCCTAGGGCTACTCTTTTTAGCTATAGAATCTCTTTGAAGTTCATAAATGCATTACTTAGGTGTAGTGAT	T/C
3	PERL_530599_Ler	AAGTGAAGTCATTGAAAATTCTTTTCCGTACGGTTTGAGATTTTTCTGAAAATGTCTACTTAGTCGATCTTTTCCTGTAAGTAACCTTGTTAGGCGTTCC**[A/G]**GAGACTTTTTCAAAAATGGAAGTATTATAGTCCCTTGCAAACAGTAGAGAAAAACGTCGAATTTTTAGATCTCCTTCTTTTGTTAAAACGCTCGTCGTAC	A/G
3	PERL_536106_Ler	CTCCCATAGATAGTGAAGCCACCTAGACCAAACCACCACCTCTATTCCCCATAGAGAACCCCACCAGGGCAACACGCAAAGAGATCTTCGCTGCAAACCA**[C/G]**CGTAGGCTGAAACTACAACAACAAATACCAGCAACACACAAGTAGTAATCAAAGCAAGTCACGAAAGAGCCACAACGGAAAAACGGAGCCGCAAAACCAC	C/G
3	PERL_539850_Ler	TTCATAACCATGTCATCCGATTCAAAGAAGTGGTGCACCATTGAGCAGACATCATTCCCTATCAAGTCCCAAAATTGTTGGTAGAAGGCAGCCGTGAAAC**[T/C]**GTCAAAATTTGGAGCTCTATTTGCCTAAATCGAAAGGACTGCATCCTTTATTTCCTGATCTAATAATGACTATAATAACTCTCGATTCATCTGATCTGTT	T/C
3	PERL_544271_Ler	AACTGTAATTGTAGAGCGAATAGGCCTGAAGCGTTCATCCAAATTGAGCAGGAATTGATGAACCTTGTCGTCTTCACGTTCTTTGGCGATGTCGGGAGCA**[G/A]**CTTCACACGTGCAAGTCCGACTGGTCTTGTAATTTTGCAGTTCTTCCCAAAGCTTGGTTAGTCTCCCGTAATAAACAAGAACAGATTGACCATCTTGCTT	G/A
3	(1) PERL_548475_Ler	AGGTTGTGAAATCATCAGGATTAATAGGTATACCAAACCTAGAATGAGCAGTTCTCCCACCTTGTAACAACAAAGAAGCAATTCCGCTAGACGCGACGTT**[C/A]**ACAGAAATTAAACCTCTCATTCTAACTGCAGCAGATAAAGTTTTCCACATGAAAGTTTTACTGGTTCCTCCAAAACCGTAAACAAAAAAAATTCCACCAC	C/A
3	PERL_551641_Ler	GAGTCGGACGCAGTGGAAATTTGTGACGATCTTGAGGATCTAAAGCCCGACATGATTACAAAGAAAATTAAAGTCAAAATTGTTCGAAGGTTCGTATGCA**[A/C]**GGCGTTACGCGATAGGGTGGTGGAATTTATCTTTGCATATTCAAAGGTAACAATTATTAATTATCGTAAATTTGTTATGCTATAAGGATTCAACCTGATT	A/C
3	PERL_554387_Ler	ACATAAATCTTAAAAAGCAAGATAAGGCAAAGAGACTAATCAAATATCACCAACGTGGTATTGGCCCAATATTAAATCTTCCAAGTGAAGTGTTGGCTTC**[G/A]**ACGCCCGATTGAGAGGGATCTCCGCTCCAAAGTAGATGAATTCAACATGGTGTGGGTCCCACCTTTAAGAGATATAAGTCCTTTGAACTTGAAAACTAAT	G/A
3	(1) PERL_556114_Ler	CACAAGGACACAAAGAAAAGATGATAATGTTATTGAACCAGGTCCAACATGATCACCAAAGTCAGTGAAAGTAACTGAAACCTTTTCATTAGGATTTTCA**[T/G]**CCACTTTACGCATCTTTGTTGGTCCTCTCTTCTTTCTTTCTTTCACTCTTTCCTCCGCTACTTTACTTCCTTCACCATCTTCTTCCATATTGGCAGCTTC	T/G
3	PERL_558058_Ler	AAACAAAAAAAAAAAATTAGAAAAAAAAAACAGAAACCCAACTTTCTCTCTCCTCTCCTCTTTCTCCTTTGAAGTTTGAGCTCGTCGCTCTTACTCGCCG**[A/G]**AATTCGATTTCAGTAGTCATTGTTGGCATATCTTCTCCTCCGGCGAGTTTTGTCGAGATAATAAGAAAAGGTATGTTTTTAGAGCTGCATAGAGCTCAAA	A/G
3	CER477616	ACGTAGTGAACAATGTTGGCAACGGAGAAGGGTAAGAAGAAGGCATGGGCTTCTTCTGGCCGGCTAGCTCTAAACCGGCCACTTGGTCCGCCCATCACGT**[A/T]**ACTAAGCTCGTCAATGAACTGTCCCTCGATTCCGTAGATATCATTTACCGGTCCATCGTGAACCAGTGGCTGCTCTCCTTCTTTGTACGACCACACCTTA	A/T
3	(1) PERL_565686_Ler	ATGGTAACCTGTTGGTGAAAGCAACAAGATCGAGGAACAGACTGTACAAAGTGGAGCTAAAAGTCGAAAACACTAAGTGTTTACAACTTGCGGCACTTAA**[T/C]**GACTCAACAAAGTGGCATGCTCGATTAGGTCACATTAATCTTGAGACCATAAAGGCAATGGTAACAAAAGAATTTGTGATTGGAATACCTAGTGCCCCAA	T/C
3	PERL_571321_Ler	ACTCATTCATAACATGGATAGCATTTCGTAATCGCCTAGCTACGGGAGAAAAATTAGTAAAGTGGAATAATGATGCTAATGGGGGTTGTTCTTTCTGCGA**[T/C]**GAGGCTATGGAAACTAGAGAGCACATTTTCTTCTCTTGTCCATATTCATCAGCAGTGTGGTCAGCCCTTACAAAAGATTTGCTTGGTCAACTCTTTACAA	T/C
3	PERL_577028_Ler	TACCCATTCATGCGTTGGGCGATGGACATCGTCGGACCTCTTCATGCGTCCCGCCAAAAGAAATACCTTCTCGTCATGATCGACTATTTCACCAAGTGGG**[C/T]**CGAGGCGGAATCATATGCCCGGATCCAATCGAAAGACGTCCAGAATTTTTTTTGGAAGAACATTATTTGCCGCCACGGGCTTCCGTACGAAATTATTACC	C/T
3	PERL_583287_Ler	AAGTCTTCCAAGTTACAATGAAACCCAATTTGTTGAGGTTGAAACAGGTGAAGCAAGAAGTGTAATGTACAAAGGTTTTGCGTCGGAGTTGTTCGTACCG**[A/T]**ATATGGATCCAGGGGAAGGTTGGTATTCAAAAGCTTACATGGATGCTGGAGAGTTTGGTTTAGGACCTTCTTCAATGCCACTTGTGCCACTCAACGACTG	A/T
3	CER477076	GATCCAACCATAGCACATCTGGGCAGGCCGGAGCAAATCGTCACAGTTGCGAGTATGGAGTCTCGCTGATAAAGGATGGCTCCTCGAGGTTCAAAAGCGA**[T/C]**GGATAGATCGTGGGTTGAGCTTGGCAAGGGAACGGGGTTGCCGGGAAACGTCTCTTAGCTTCAACATCCGAAGAAACATCAAAGAGCTTTGGATTAAAGG	T/C

3	CER477021	TCCTCCACCAATTTATTTCCATCGAAGTCACTACACTTTACACTAATAGTGATATTTCTGTTGGGACCTGATCTTCATAGGAAGTGCCTAGTTCTAGATC**[C/T]**GATGCGATGAATTGATTAAACTCTTGGTGAAGCAATTTCTGAACGACATAAATAACCTGAATGCGTTGTTGTTCTTGGGATAGTATGTGATAACATGTTT	C/T
3	PERL_597178_Ler	ATCCTCCACTGGCTTGTCTTCCGGTGTGCGGTCTATGACCCATTGCACCATAGGCTTAAAAACAAGAAAGACGACGAGAAAAAAGATGATCACCGCGACA**[G/A]**TGTCACGATAGGCTGTTGCATGTGAAACATGAATGTAGGTCGCTTGTATAGAGGCAACTATCATGGAAAAAATCCCCAAGATGTCGTTGATGACACATGC	G/A
3	(1) PERL_600735_Ler	ATGCAAAAGATTTGACCGCATTTGAAGCAACATCTCCTAACTTCATCTTCTTTAGTTGATATAGAGAGCTCGGTTTCTGACATTAAAGCCGAGCATCGCG**[A/G]**GTTTGGGCAGTAAATTCTCCCTGTTACAGGGATTAAGTCTTCTTTAATCCTTTGTTGCCACATCTCTCTTATTTTAGGTGTCAAAAGATTTATACAGCCT	A/G
3	CER478878	GTCGAATCTTTCTGTCTGATAAGCTCTCTTCGAAGTTACTTACTTCGTCCAAAGAGATTCTCGCAAGCGTAGGCGGTAACTCTGGCTCAGACATTTACCA**[C/G]**ACGGCAAGAGTCTTCACCGAAGTCTCTAGCTACAAATTCTCGGTCACTCGTGGTCGTCATTGGGTTCGTCTCTATTTCAATCCTTTTGACTACCAAAACT	C/G
3	PERL_609298_Ler	TAATTCGTTTGTTCTCCATAGTAGATATTTGCTTCTTTTGCTTTATATACTCAAGTTCAGACTTTGTAATCTGAGGATGTCGATCTGGTGCACTTGACGT**[T/C]**GCAGAGAGCCACACGAGCAACCAAAGGAACCCCGATAGTCCAAAAATTACAAATGGGCCATATATACCTCCTTGAGACATAAGAATAGGAGACAACATGA	T/C
3	PERL_612997_Ler	GGGATGGGAACCCGCCTAAAAAGGCAGTTCGAGTGTTATCCCTCGCTGTGCCTTCAGGAGAATGCTTTGGCATGTTAGGGCCCAATGGTGCTGGCAAGAC**[C/T]**TCTTTTATCAATATGGTCAGTACACAAATCTACCCCTAAAAAATAAATAGTTGTGCTTAGCTTATGACTGAAAAACTCCCCTTGGATAATAAAAGTTGCA	C/T
3	PERL_615911_Ler	GTTTTACCGATAGCTTCTGATCAATTTCCAAATTGCAATCTCGCAGTCGAAGCTATAGAAAAGCTTTATATAGATCGTGAACCAGAGGAATTTGAATCGT**[T/A]**ATCGAACAATCCCTTGTTCGGTTTCTTGAAGATCGATCACTACCGATTCCGATCTGGGCGATTAGATTTGATGACAAAAATTACGGTGTGTGTGTTCTTC	T/A
3	PERL_618967_Ler	TGCTTCTCTGTTTCTCCGTCGAGTCTCCCGGAGATTTCATAGAATCCAATCGCCTCAAATCGAATCGCTAATCTAGTTGAATCAAGATGGTTTATATCGC**[A/T]**TCGTGGGACGAATTCGTTGATCGATCTGTTCAGCTTTTTCGAGCTGATCCGGAATCTGTAGGTTAACTATTTTTTTGCTCTCCTCTTTCGATTTCACTAT	A/T
3	PERL_624470_Ler	TTTTTTCCACACCGATAGGTGATGTTGCATCAGATAATCTCATTCTTGAAGTTACAGATAATCTCATTCTTGAAGTTACTTATCAATTGGAGCATGCCAT**[C/T]**GCTAACTGATGCCTCTTTAAATTACAGAGTCCACTCTGATGTTTGCTTGTAATGGAACCCAAATTGGGCTGGCAAAGTTGTTTCAAGGCTACCGTCTCTC	C/T
3	PERL_626784_Ler	AGACGAGGTACGTAGCTAGTCTAAGAAGATGTGATGATCAAGCCTTCGTTCTTGAGAAATTGGGGAGAAGATGGAGGCAGTGAATGAAATGAAGGTCGTG**[T/C]**ACAAGAGGATTTCCGGTTCTCCTTGGTGGAGACCCTACGTACCACATTGACTCTATGGTTCCACCTTCTATCGGCTGCACCTACGACCACGATGAACATT	T/C
3	PERL_628544_Ler	TACTAGAAACCCTAAATAGCTAAACCACGCAATTCCTGAGGAAGATTCAATGTAACGAGAGATACGCATGCAGAGAGGAGAGAAAATGCGCAGGACGAAA**[G/A]**CATACTTGGGTCCCTTCGTCCTCGTGAGCCAGAGGATCGACGCCGAGGATGACTTTGGCGTCAGGACGGTACGGATCGTCAGGACGGTACGGATCGTCAG	G/A
3	PERL_630835_Ler	AATTTGATTGGTTTTTTGTTGGCTACAGCTATGGCGGATACGGTAGAGAAAGTTCCCACCGTTGTTGAGTCATCTTCATCATCCACCGTTGAAGCATCTA**[A/C]**TAGCGCCGAGAAGACTGAGCCCACGACGGAGAAGAAAAAGTGGGGCGATGTTGAAGATGATGATGATGAAGAAGAAGCTGTATCGGAGCTTAATTCATTG	A/C
3	PERL_633276_Ler	GAAAACCAAAACCTGTTGCTTCGAGAGCACAGTCCAGGTAATTTCCCTTATTAAGCACCAAGAATTTGATGCCTCTTAGTGTTTGATCATTGGGACTTGC**[A/G]**ACGAACATATGATTGTAGCTTGTATTGTCACTGAACTGTATTGGCGATGTAAACTTAGATTTGCTCATTCGTATAGTCTTTTTGACAGAGTTTTAAACAC	A/G
3	CER476104	AACTGGCAGACACGTTAATCTTCAGTTTCTCAAACCCATTTCCAGTTTCAAGAGATGATCTTGAGGTTAAACTGCTGCTCCGTAAAGAAAAACGAAAACT**[C/T]**TCGGTGCGGTTAAAGTCTTGAGAACCCATATTATTAGCCATGGATGAAGCATCACCACCATCATACTGATGACAAGAGTTAGTAGCAAGGTTGATCCTGT	C/T
3	PERL_637620_Ler	ATTTGGGATCATTGACACATTTGATTTCGAAGCATGCGCCGCAGCTTTGACCACTGTTAAACAACGATGTGCTCAGCGCCGCCGTGTTTGTTCCGTATCC**[T/C]**TGGCTATACAGATTCCCGTATCCACACGCGCCACCTAAAATAACAACAACAAAAACAAAAGTAAATTAAAGGAACAAGAAACAGAGCACTCTGTTTTAGA	T/C
3	CER476418	CTCAGATGAATCATCCCAAGATTCCGACGCCGGGTAAACAAATGGAAGCTCCTCGCTCCGTCGGAGGAAGAATACAGAGGAAACCGCTCGCGGATTGTAC**[T/C]**AACACCGTCTCCAGATCTTCTCAACAGTCATCTTCTTCTGTAAAATTCGCGAATCCGAGTCTCACTTCTTCACTAAAGAGATTAGTAGAACAAACTACGC	T/C
3	PERL_642202_Ler	TTTCCGCTCCTCTGAATTCAATCTCAGGGACATAAGGTAGGGATGCAAAAGGCCGGGAGTGTCATACATCTTAGCCTTTGCAGACAATATTCCGCCAATT**[T/C]**TCAATATTCCAAGAGTTGTTCCAGGAACTGGAGCTTCCGTGAGCCTCGTGACCTTTGCACCATCTTTCTTGGATAAGGCATTAATCAAAGTAGATTTCCC	T/C
3	PERL_644299_Ler	TGCAGAGGTTGTAGATTCAGATATGAGCGAGGTAGCTGCTTGGCCAATTAAAGCCTTTCTTAGATTAGCGTTGAGATGCATTGGATGCAAGTTGGGAGAC**[C/T]**CGTTGAGTAGCATGATTCAAGTGACGAAAGAGCTCAGGCTGATTGAGAAATCTGCATATTATCCATCAAACAACAGATCACAGATGAGCTCCATATGACA	C/T
3	PERL_646360_Ler	TGAGTGTTTGCTTTTGAGTATGATTTATGTTTTATTTTCTATCGTAGTTCTTATTACATTTTTGAGCCATTTCATATCTTGTACTACTTCTATGAAACAT**[T/C]**GAACTGGTGAATGTGGGTTTGATGGTTTTGTGGAGGAAACATACTACTGTTCACCTTTTAACTTTTTAAAGGTTTTTCTTTCTAAACAAAGACTATTTAT	T/C
3	PERL_648257_Ler	AGTATGCAATAGAGAAAGTCTCGAAAGTGTTGGAAGGTTGAAGATAAAGAGAATACAGAGAGGTAACGGAAAAATAACGACTGCTTTTTCTCCAAAATGA**[T/C]**GACGTTACAATCATCTCCATTACGACGTTTTACTCTTTTTTCAGTAACTTTTTCTGTAGTTTGTTTGGCATCACATAGCAATGAACATTTTATGAAATCT	T/C
3	CER477934	TAGATTGTACTAGTTTTGATAATCAATCTTTCAGATGATAATACTTAGCCAGAAGTGTTAATTTTGGATATTTCCAAATGAAAAGTGTGAGGCTTTAAAG**[A/G]**TGGCAAGCGTGTACCAGAACTCCATCACAACCATGTGGCCATCTAAAGATAAGATCAACTAACGCATTCACTCCTCCAGCTTGAGCAATCGCGTTCTAAT	A/G
3	CER482305	TTTGTTACACTGGTTTTAGGAGTATTGGACCTGCCATGTTTGAGGGAGAGGCTCTTGGTCTTGAGGCTATGTATGAAACCAGAACAATCCGTGTTCCAAA**[T/G]**CCACACAAAGCAAGTTTAATCTTTCTTTTCTTTCTTTGGCGTATATACATTATTATTTTTGTTGATCTCTCTAATCATAGATTCTTGTTTAAAAGTGTTA	T/G
3	CER478092	TGTTTCCTTTACTTTCTTTGTTCTTTATTTTATTCTTTTGCTTTAAATTCACACTAATTTTCCTTCGCTTTAGTTACCACTGTATCTGTGTATAGCCTTA**[G/C]**CCTTATCATACACTAAAACTGCGCCTGGGGCGTAATTTAGCAGCAAGAGGATTAGCCATCTCACACGAGAGCCTCAGTTTCTCGGACAAGTCAACGGTCT	G/C
3	PERL_656311_Ler	GATGGGGTATCAGGGGTCATCACCACCTTGGAAGAAGGTTGAGAATGGATCTCTTGTGGACTGCTCTTCACATTTGAATTTACATCTTCTTGTCTCTCAC**[T/A]**AGGCAAAGTAGGCGATCCGGTCTGGTTCGAGTTCCCATTTTTGATACTCATGAATGCAACTTTAGCAGCGCTCATTTCAGCCAACTTTTTGGTTTTCGCC	T/A
3	*PERL_657590_Ler	TTCTGCATTCTGGAATTGTTGGGGCAATCATTTGTGCTCTTGTGACCAATTTTGCAGATTGGTGACCTTAAGGGGTTTTTCGGATTGCTAATGGTCAAT**[G/A]**TCCAACAGCTGAGAACGAAGCTCAAAATATTGGACAACACTTATGGAAATGGAACTGGTTAACTACTTCATCTACTTTTCTATTCTTCCTCTGTTCTAC	G/A

4	131	CTGAATGTTTTCCGGATCTGCTGAGTCATAAATACGAGTGCTTTAGGCAGGCAAATAAGACTCCAGCATCAATTTGGCTGGGGAAGGTGTTAAAATCTC**[T/A]**ACGATCAGGTACGTGTATTTCCATTCCTTCTCTGCCTTGTCTTTAAACATCTCCGTGTACACTTTCCCTGCTCCTTGAGGCCC	T/A
4	130n	GTGTCTTCTAAAAGAAGGCGTTAGACTTATATATATGTTTCTTTCAGTGTTAGGGTGTCGAATGAGCTAGGATCGGGACATCCACGAGCTGCGAAATACTC**[C/G]**GTGATCGTAACGGTGATAGAGTCTCTCGTCATTGGAGTAGTGTGTGCAATTGTAATTCTGATAACACGAGATGACTTTGCTGTGATTTTTACGGAGAGT	C/G
4	128ac	CAATGAAACAGCAGAGATAATCAAATTCATACAAAAACATAAGCATTTACTAGAATATCTAAAACTAAAGATCTCAAAAGCTTAAATTTGACTTGTAACA**[T/A]**ACTATAAACGACATCAATCCGTAATTTCTGGATGAGAATGAGAGAAAAATTTCAACAATCAGAAATATAAAATCAATCTGATCTTGAAACGAGAGACATT	T/A
4	128	TTAAAAATCTAACTGTATTAACTTTATATTTTAACTATATAAACCTCTTGAATATATTATATCATGTGCATGTTATGGACTAAACTTTTATTATTGTTG**[T/C]**AGCGTTATTGGATCTGTGGTGGTTGTGATCGGTTTATATATATTTCTATGGAGTAGGAGTAAGCAAATAGTAGAGTGTAAGATCATGAAGTTGCCTACC	T/C
4	127	CCAGTCCGTGGTCGTAACTGACAATCAATGTACTTCTCAATATGGATGCTAGATACTTGCTTTACAAAAGCTTCTACACATTTAGTTACATCCCAAAAG**[C/G]**CTATGCTTCCATCTGTGGCCCCACTGATGAGCAGATAAACATCACTATATGNTGTATTACCTGTCAGGTAAAGTTATCAAAACTATTACGACAAGAAGA	C/G
4	126ter	GAGCTCATTTTTAACATTTGTTCGTACCAATTCTGGGATTTGTAATTAGCTGAGTATTGAGCTCTAAATCTTGAAAAATTAGTGAGTTTAGTATTTTGC**[G/A]**AAAGACCCATTAACAGATTATGATTTGTGCGTTGTTTCTTGCCAAATACTTTTGAGAGGTTTATGAAACAGAGTAAAACCCTAGAATTCAATTCCTTTG	G/A
4	125	GTCTNTTCTTCACAAGAAGTGGATTAATGTTGAGACTGAAATGGCTTGAATCTGGATGAACCTTTACCAGCAGAGATAGTCTAAGACTCTAAGAGGGTC**[A/T]**GTAAAACTTCCTCTTCCCTTTTTCCATTTCAAAAAAACAGGAANCGAGGCNAAGAGGAAGATCACTTAGCAGGAGAGTAGTTAGATTTACATCATTTAAGG	A/T
4	123	GGTTTAGTGGTAAAGGATATGTTTGTTCCTTAGTGCCTTTTCCTTATGAGGATTAGGAGAATTATTTTGTTTAATTCGTATTTGACATCAACGTATATTGAGTATGGAC**[A/G]**TACTGTATATAGGTTCCACCCGTCTCTCTTCTTATACTCGCAAGCATTAGCACACAGGTTTTGAGTTCATCGCTACTAGTACAGTTTCATAATGATATTAT	A/G
4	103	CAAAACCATCAATCATCGCGTACGAGCACGAAAGCACGTAAAACCCGAAAACTAAAGCTATTTTGACCTCAAGCAAAGCCAATGAAATTCACACTATAT**[G/A]**TCACCAATCAGAGCCTATAATTTTCCATTACTACTTAGAATGTCACAACAAAAAAAGAATCAAGTAAAACCTTATCAAACTCTCTAGCTAATCATTTCT	G/A
4	102	CCCCCAAAAGAATCCTTAAACCGTCGCAAATCGTTACTTTGCGACTAATGTGAGACGATACATGTTAGTCGCTATAAAACGGTCGCTAATTAAAACGGTC**[A/G]**CTATTGCGTCGTCAATTTGTGACGCCTTTGAGACATATATATTCGGTCGTATATAAGCGACAGTTTTGCGACAGTGGTTAATTGTCGTATATTTACGAC	A/G
4	101	GGCAAAAGACTCACCGAGACATCTCGAATGACGCCTACTTCGCTGTTGTGTACGGTAACCAAAATATCAGGCATGAACCGCACTTCAACATCCTCATCT**[G/A]**TGGCAAATACGAAAAGCTAAGAAAATAGCCATAGATGTGAAACATCTTGCGTATGAAACGTTGACGAACATAAAGGAGATGCATGGAATTCAACAATAC	G/A
4	99	TCAAGTAGGATTGATTACGGAACGGGACACGAAACAAATTTCGCGGCGTGGTTGTATTGTTTAGCTAGGATGGGGATAGTGAAAGAAGAGGATTATCAT**[A/G]**GTTTGGTTGCTAGGGTTTTTGTTAAGTATTTGGAGTTGATGAGGAAATTGCAAATGGTTTATTGTTTAGAACCAGCTGGATCTCATGGAGTTTGGGGAC	A/G
4	98	GGAGTTCAAAACATAGNAGGGATGATAAGCTNTGGAACGACTCCCATGCCTTCACCAATNTACTCTGCTACACCTAATCTTGGACTCGCATCCACGCTT**[T/C]**GAGTTCATCTCGCAATCACAAAATATTTTGTCAAGGATCTGGNTNCTACATATTTAGTATTGTTNTCGTTCCTCGCTTTCGAGTTANACCAAGCTAACCAA	T/C
4	94	TCGTATAAGATAAGTTTGGCTAGACCGGCTTTGAAGTTCAATGAAGAGGCTGTTCTTGCTTTATACTCAGGTGAGTCACCATCTCATCACAATAGATTC**[T/C]**CACTGTCAATTCTCTCTCGCTTTCTGTTTAACGTGTATATTTATGGTTCCAGGAGATGTGAAATCTGCAACCGGGCTTCAAACATACTTGTTATCAAGG	T/C
4	91	CAAGACTCAGATGCAATCTGTGGTTAAGAAGATGAAGCATTCCCCTGCATCAGCTTATAAAAGATTGACCGAGCAGAACATCATGAGTTGTCAGAGAGC**[T/A]**TACGTTACACCCTCAAAGATCTATCTGTTGGGTCCAGAGCTTGAGACTGCAAATTATGTTGTGAAGAACTTTGCAGAGCATGTCTCGGATTTCATGAGA	T/A
4	88	CTTGTTGTTTAGTAACGAAACGCTGGATTCCACGGAGTTCCCACGGTATGGTGAGGTTTTACCGCCACGACAAGTCGGAGACAGACAGGTGATGCCGTT**[G/A]**CCGGATGAAGATGTGTTTAGGTTTGAACGTGAAAATGCCGGTAATGCTTCATTTAAATTCCAAGAGATTCCTTTCACCAACGAGTCTTTGTGATGGAAA	G/A
4	86	TATCTAAACTAGCCAGAACTATTGTCGAAGCAACAGTAACTATCTCACAAATAGTTAAAAATCACAACCAGAGGAGCTCAAGAATTAAGCAGTTTCCTTTC**[A/T]**AATCCTTTCGACATAGAATCTAATGAAGCCTCAAAACCAAAGAACATCAACATAACCAAATCTGTAAGCTATAAAACCACTTAATGGACTATAAGTAAA	A/T
4	85	AAAAAAAGTTATAATAAGGTAAATGGATCGTCCCAAGATCATTATTGGATAATTAAAATCACTGGCCAGTGGAATTAAAGCCCAAACAAGCCCATGATA**[A/G]**CTCAAAACTAGATCAACCAGTAATGGCCCATGATGTCGTACTTGAGGTGACGTGGCAAATTCTGAATTCGCGCACCGGTAGATTCTTTTCTTTTTCCGT	A/G
4	83	CTCTTGGAGATCCAAAGTTGTACATGGTAACAGTGATTGCCCCACGTCTAACAAGCACACAAATCATGTTAAATGCCCATTAAGCTGCTTGTCGTCAGG**[G/T]**TGATATGTAGGAGAAGGGAGTTCTCAAAGTACTTACTTAGCTAATTGGCTGGACGAAAGTTCGAGAGCCAAGAGTGTAGCTAATGCGCCACCCAAGCTA	G/T
4	80	TCTCCAACAAAGTTTGGTAGCTCAGATGTGGGATATGAACGTGGAAACATGCTAAGACCAAGTACTGATGGTTTTCGAATTAGTGTCTCAAGCACACCAAG**[T/C]**TGTTCAACTGGAACTTCTGGTCCTGATGACATAGAATCCTTAGGAGATGTTTATGTCTGGGGAGAAGTTTGGAGCGACGGGATATCACCAGACGGGGTC	T/C
4	78	TGCTAAGAACTATCTGGATACAAAAGATGGCACTGAAACATTATTTCAGTAGTAATATGAGAAGGAACTAATAACTGGAACTCATAGCAGAAGCAGAAG**[T/A]**TCCTTTGTTTCAATCCTATTCCTGTGGAGAGTGGAAATGGGCAATGGAAAGTATGAAGAGCCTGAACTTGAATAAAATCATAAGTTTTGCTGCTTGGCCA	T/A
4	75	TTATATCCTAATTAGAATGATATTGCCACACTCTCTCCCTCAAACTACTAAGGACCGCATACCCTTGGCTGTTCTTCCAGCAAAACAATCTTATACCATA**[G/A]**TCACCAAGAAACTCGCCGCTTAACCTTTGAATAAAGGGAAGTAGACTTCGACACAAGCTATCAACACTATTAATTATGATGTCACATTGAGTGCTC	G/A
4	66	GTGGCTCAGGAAGTGGAAATGGTGAAGGATCTCCTGGTCGTAGAGAGAGGAGCCAACACCGCTAAAAGAAGTTTTCTTGTTATTATTACTACCTTAACA**[C/T]**AATCACACATGCGTAAGATGATAAGACTAAAAGAAGGAAAAAGAAAAATACTATAGTAGCANCTTAATTAATTAATGCATAAATAAGGACCAAAGGAAT	C/T
4	64	TGAGAGCCATCCAGAGTCAGGGATGGTTACTTGATCACGAGTAGGGCTTCGAGCCAATGGCGGAGTCACGGGAGCGCTTATTGAATTTCCATGGAAGAA**[G/T]**GGAAGCTTGGAAGGTGAGTTTGAAGAGAGGTTCTTGAGCCATGGGATTAGTGAGTTAGCATCACCAAATGGGTTTGTAGGACTCGGGAATGATGAAGAC	G/T
4	62	GCTCTTAACCAGATCAGATTTNTGGTTATACTATATCACGTANTTCTGCGGTGGAACCATTGGACTTGTGTACAGCAACAACCTTGGACAGATTGCTCA**[A/G]**TCTTTAGGACAAAGCTCAAATACCACAACTCTTGTCACACTTTATTCNNCTTTCTCATTCTTTGGAAGATTGCTNTCTGCAACACCNGATTATATCCGAG	A/G

4	57	AGAAAGGTTGATTGTTACCTCGTTGGGTCTCAACAGAAACCATCCGAGCAACCGAAGGTTGATTTACCCTTGCCCGTTTGGCACGACTACTTCCACCAA**[T/C]**TGGTATGTTTCTTAAAGCCCCACCATGAGTCCAGTATCTACGACAGTTCTTGCAGAAGTATCGTGGCTGAAACTCACAGTAGTTGTTGTAATAACAAAATT	T/C
4	55	GACTGGTACTCAAGAAAAGACGGGAGCCAAAAGGGTAAGCAGTCTGTTGTTACATGCTCAGAGATTTGTTGTTTTGCTTGATGACAAGTGGGCTAACGT**[A/G]**TGCAATTGTGTCAAATTGAGCAGGTTGATTTGCTGAGAGGGCAGAAGACAGACAGAGTGGATGTCAGCTTACAGCCAGAAGAGCTGGATGCTATGGAAA	A/G
4	53	AAAGGACCAGCGAAACGAACAGAGTTCTGGACGGTGAAGTTATCTGAATCAATGGTTTGAGAGATCTCATCGACTTTTATTAACGGTACGATTCCTCGTGA**[T/A]**AGCAACGGGAACAAATTCACGAACGATGTGTAGCTGCAAAAGAGGTTTAACCAAATCAAAAGCAGCACGAGATCTACCATTTGAATCCAGAATCCTACG	T/A
4	48	TGACAGTGCACTTTTGATGTAGGCTTGTAATGCACATGGACTCTATTGTGTACCGTTATATGATACACTAGGTATGCCTTTTTTAGTATTTCCCAATATAC**[T/A]**CTAGAAGGATAAATTCGCCAATAGATTTCCAGTGAGCTTCTACAAATCTTTTAGCTAGCTTATTTGTCATTTTTGTTGCGTGTCAATTTTATAGGTGCTGATGCTGT	T/A
4	41	TTCTATTTTTTCTTGGTCTTCTTCCTCTTCTTCGATAAGCGGCTGGTCTCCTTCGCTGTTTTCCTTTTGATTATCGGCTTCTGCAGGTTCTTCAGGCTG**[G/C]**ACAGAGTGTTCTGCTTCTTCTTCTTCTTGTTCTTCTTCCTCTTTTTCCTGATACTCCTGTGAAAAACACAACAAAATGTCAATCATAATTCATAAGAAG	G/C
4	40	CAAAACTTCTCTCCAAAATCATTTGATTTTGAAATGAAAATCCTTTTCTTCAAGCCAGTAGCATTCCTCAGGACATATTTTACCACTTCTTTATCTCCTTG**[T/C]**CTCCCTTTATACCCACTCCACGTGAAAGCTTGAAGACTGGACAACAAACACGCGGGAACAGAACTCGGCCGTTTCCAGCAGGCCGGGGTCTCTAAACTA	T/C
4	37	TGGAAGAAAATTCNAAGTAAGTTTTAAGTAGTGTTTTCCCTGTTGGTCTTGTGAGTTGAGTTTACTGTTTCTGTGAAGTTGATTGATCCTGATAAGGGT**[G/A]**AATTGAATGGAATAGGGTTGGTCTTCAGCGTGTTCTGGAGAACCGTAAAGCGGCTTTGTGCAAAGAGCAAGCGATGGCTTATGCTCGAGCTTTGGTTGT	G/A
4	35	TGTTAGGAAGTATCACATTTGTTAGGATCGGACACGTGCCATCATGTGACTTTGCGTTATGTTTCTAATCTAACAAAAAGCCTAGCATATAGAGACCAT**[G/A]**ATTAGTAGAGACGAACCACGTGAGGGCTCCCTAGATTTTGCTTGAGTCTTTGTCTTTTTCTCTTTTTGAAAATCCATTTTTACGCGTATGAAGCCTAAA	G/A
4	31	TGGTATCATATGTTACACATTTTATCACTCAAATCTGTTTATGAATCTTATGATAGACTCAATTATTTCAACAGATTTATGGTGGTAGTACAATGGCTA**[T/A]**TGATGATTCGACATATGTAAATAGGTTATAGTGAGAGTCTTGTGGTTTTAACTTGTGCATTGTAAACGTTTTAAGTGGCAAATTTTATAGATGTCGGAG	T/A
4	29	AGGAATGCACAACAAGCTCATAGAATCTCAAATTATATTCTATTTATTTATCTACACTTCCTCTCTTTCTTTTATGTGAAAATTGTGAATGCTCTGTTT**[A/C]**TAGCTTGTCTATTATGTCCGAGAATTTCTTTTTCTGTTTTTGATTCTTTTCGTTGTATATCTTTGTCCAATAAAGGAAATGATGTGTCTTTACTCTTAT	A/C
4	25	TTTCTCTTTCTCCCCAGCTTAATCTTTCTCAGGTTTTTAGTATTAAGTTATAGGATTAAGAATGCAACTACAGATTATAGATTGCTCAGAATCATATAC**[T/C]**CTTTTTAGCCTTTATTAATGTCTGATTCAAGTCCTAGCTACTGATTTGAAACCTTGTCTTGGCTGATTTCTTCTGATAACTCTTGCGTTATACTTATCTTT	T/C
4	19	TGAGTCACAACAACAGCGAGTGAGACGATCAGTGTCGTTGGATTCGTTATCGGGTTTAAGGGTAAGTGAAGTTGTTGTGGGTAGAGAAAAGGAGAAGTT**[G/C]**AAGAGAGGCAATAATATTGGATCTGGGAGGTCAAGTTTATTGAAGAGATCTATCTCTTACAGTGGAAAGTAGTACTAGCTAGCTTCAGTGCTAATAATA	G/C
4	17	TGATTCAAGACACCAAAACCGCAATGTGAGAGACTTTAAGACTAAAAATCATGGATAAGACTAAAAAAACATGGATAAGTATCAACTGTTCTCACGATT**[A/G]**TTTATTCATACCACTGTACTTAAACTTAAAACCCACTATACTAAATAGAAAGGTAATCATCAAAAAATCAGTATGTAAAAACCACTTTTGTGAATAAAA	A/G
4	15	CATCGTTGCGGTTGTTGTTGCTGTGTACCTTCAGTCTTACGACATCATCGGAATCAAAACAGGAGCTTTCTCAATCGCATTCGCTTCCATACTTCTCAT**[A/G]**CTCTTAGCCTCTCCTGTCGCTGTACCTTTCCACGCTTTTATCCGTAGCAAAGTTCATGATGAGCAAGACGTAGAAGGACGAATAGATGAACCTTTACTA	A/G
4	14	TCGTCCATTGTACCATGATTTGAATGCTTATAGGGTTCTCTTTGATGAGGTATTATTATTTTCTTCTCAAGAGTTATTTATTTCGTGTTTAAGAGATGC**[C/A]**CTGTCTCGTTGCGGTGCCTAACAAGTATATTGGAATGAATATGGTAGGATGGTGATCCTCGTCTCTCATGTTTTGGCTTGATGAAGAACAGTAGGGATG	C/A
4	9	AAGCTCATGCCTCCATACATTCGACCAAATGCGCGCATAGGACCTCCATAGCCACCAAAATATTCAGGACGAGAAGAACTTAACATGGAACTTGCGCCA**[G/A]**CGCTAGGTCCAGCTTCATCAAGAGGCGTTGCTGAATCTATTGCTACCTAAACCCGAAAATCAAAACAGTGTAGCTTCTTTATACATCTTAGATAAGTTT	G/A
4	7	GTGTTATTGTGAGTGGTGTTGTGCTTGCAAATGTGCCCAAGAACTCGATGATTATGATCTGAAAGAAAACCGTTGCACCGATCACAACCACAAAGACGTA**[A/G]**TTGTCCAGTATTCCTTTGAAAACATCGATCTCTTCCATCTCTCGCGAGCTTATCTCGTTAAACACCTGGAGTTTTCAGAACCAAAGTCATGTCAAGAATC	A/G
4	PERL_848945_Ler	GCAGTGTGGAGAAAATAAACGTGTTATAGGTAAAGCAACCTCTGATGCTGACCTTCCTTCAAGGTACTTTTTTACATTCCTCTTGTCAGCAATCTTTGTG **[G/A]** GACAACAATCTTTGTTGAAACTTTTTCCATCCCATTTTCTTTGCTAGCGCTCTTGATCAAGCAAAAGCCATGGCCATGCACTCTCTCTTGCAAAAGACG	G/A
4	2	CATTGTTTCTGATATTAACTAGGTGGAACTTTTCAAGGGTTCACCATGAGCCATATAGCTTCTCTTTAACTAAAGTCCTTGCAGGAAAGTTGGAGATCA**[A/C]**ACTTTATTGTTGATTGGACCTCAGGGATACTCTGTCTTGAACATCATATCAAGATACAGAAGTCTCTTTGAAGTTTGGCAACGTTGCTTTTCTTGGTCATCTATATTAT	A/C
5	*PERL_853156_Ler	TACGAAAAATTCATGATCAACCCTCAGAAATACACTATCTGGATGAATATTTGATAGAGTACGAGATTATATATACTATGCGAAAGTAAAGTTTAACCA**[G/C]**AGTTTGGAAATTAATGAGCAGACTCACAGTCTTTTCCCCTGAAGCATTTATGTACTCCACGCTAAATCCGATATCCTGGTAAAAGACAACAGAAATATA	G/C
5	PERL_855702_Ler	AAGAGAAGAATGGAGCAGCCGTACGTGTACGCATACCCGCAAGGTAGTGGCCCCTCGGGAGCTCCGACTCCACAAGCCGGAGGAGTGGTAGTGGATCCAA**[A/G]**GTACTGCGCTCCTTACCCTATAGATATGGCGATCGTACGGAAGATGATGTCGTTAACGGACGGCAATTTCGTGATAACGGACGTCAACGGAAACCTGCTG	A/G
5	PERL_858841_Ler	CTGAAGGCCTGAATTGGTTAGTGGTTACATTACCCGTCCATATGCCTGGAAATATTCGAGATACCCACACTTACTCGCTCACTCATGTGCATAATTGCTC**[T/C]**ATCTCCCATGCATCTGTGTGTCCTCACTTGTTGTTTACGGGTGGCTTTTGATATAATAATATTAGTATAATCTCGACTAATATTGTTATAACTCGATATC	T/C
5	PERL_862557_Ler	GTAGCGCATCAATAATAAATTGTGGAGCATAAAAATGATAATAAAATGACTATTGTTTGATCTTGGAGTCCAAACAAACTGGATATCTTCAAACCGATGT**[C/T]**GCCAGGCTTTTGCTTCCCGAATCCAAAATTAAGACTCTTCCCGTTGACCAACTGAATGACTTGTTTAAAATCACCTTCAAAGATAGCTTTGGATCCATTT	C/T
5	PERL_864956_Ler	CTCTTTTCCAATTCCAAGAATCTTTTTAACCACATGTTTTAATACCTCCTCTACCTTAGACCCAAAAATAATAGATGACTTGGAATTGTTGATGACTTGA**[T/C]**CCGAGGCTTCCCCATACAGTTTCAAGCAACGCATGATCTCTGATCCCTCATTCGCTGAGGCTTTGCACAAGAGGAGACTATCATCAGCAAAAAGAAGATG	T/C
5	PERL_866977_Ler	TCGTTCATTATTATAAGATCATGAACGGTATGTTATATGAGCCTCCTGGAATTAGGTTATCATAACCCTTCCCGAACCATGAACAGTCCATATTGCGGCT**[A/G]**CGAAGTATACAAGATGTGGACAAGCCTCAAAGGATCTCTTATCTCTCCTACTTCTCTTCTATTCGGACCTCAAAAACTTAATTCATATAATTTCTAATAT	A/G

5	PERL_869153_Ler	AAGGAAAAGAGTAGGATATTTAGGTTATTTACTATGGACACTCTATTTTGAATTCTGGTTATCAAGTTTATAAGATTGTTTTGAGCCTTTGTGTCTTAGA**[A/T]**TCGGAAGCAGAGGACTCTGTTCCTGAGTTCCCGCCGCTTCAAAAGAGAGATCTTTCAAAGGTTTTCGGGGAGTTCGGAGAGAAACTCGATCCGTTCTTAC	A/T
5	CER482823	ATCTGCTTATGTAGAGTCTCAATCATTTAGGTCTAGTCTAAATCTTGACACTTTTGGAATTGTTTTGCAGTGGTGGGCAATGGAACTCAGGAGGAGCATG**[C/T]**GATAGCGAAACAGAACCGATCAAGAACGATACATACCTAACCCCGTACCCGTCTAAAATGAAAGTGCTTGAGAAAGTGTTAAGAGGTATGAAAACACCTG	C/T
5	CER477094	TAGTTTCTCTTGCTCCGGTGTGTAGAAATATATACCTGATCGGTATTGTGCTCCCAAAAGTTCTCCCTGCAACAGTTTAAAGATCGGTTAGGAATTGAAC**[G/T]**TTGTCAAGATCACATTCACTATGAAAGCTATAATTGAGGACAATGACAAGCACCATCAAATTTCATTTCAAGATAAGAACACAAATTCAGTTTAACCATT	G/T
5	PERL_875329_Ler	TACAGGATCCAATTAATGAACGGCTCTCGTTCGTCCTCAAGCTTCAAATCTGTATTTTAAAATATCAACGGCCAGGATTCATAAATCCCTCGGTGGCGCG**[C/T]**AGAGACAGTCAGTGGCCATTTTCGGCGACGGAAGTAGGCTCCGCCGGCTGCTGCTGCGTTATACAATGAATGTTTCCTCCAGCAAGAACGATCTCTCTTG	C/T
5	PERL_877303_Ler	GGAGAAGAAGAAATGAAATTGAACAAGTTGGAATGGAAGAAGACAAACTACTTGAGAAGAAAATAATGCAACGAGTAGTTGTGTTTGTGTACTAGTCCTG**[A/C]**ATTACGGGTTCCAGCTTTTAGCACAATGTTTCTTGTTTTGTCTTGATGCACACCTTGGCATTACAGAAAAAGAATAATTGAAGCTAGAGGAATAAACCAA	A/C
5	CER482707	ATGTGTCATAACATAAGAAGTTATTAACAGCGTAACGTATATGAACCTTATCCCACTATCACTATATAATAATAACGTCATACTCATATCCCAACTTTAT**[C/A]**ACGCTATTCTCAAACAAGCAAAGAACCTTAACCAAAAAGAAAACGTCTACGGAGACATATGGAGAGGAAACATCACTTCGTGTTAGTTCACAACGCTTAT	C/A
5	PERL_881677_Ler	GTTCGGTTTGATTTTTTCGATTACGGTCTGATGTAGATTAAATAAAATAATTAAAAAGTTCCAGAAGCATGCAGAAGAAGATTGAAGAACACCGAGATAC**[C/T]**GAGATTTACAGAGGATCAAATCAAAAGCAAAAGAGAAAGAGAGAAGAAGTAGAAGAATCATCATCTTTAAAGCCAAAAATATCAATAAATTAGATTAGAG	C/T
5	PERL_884176_Ler	AAAATAATAATAAAAAAAAATAGTTTCCACAGTTTATAAATAAATAAGAAAAATAGTAGTAGAGCTTAACCTGCGCCGACATCTTCGAAAGGTAGATTCG**[G/A]**TTAAAATGCGCACTACTCTTCTTCGTCAAGCTTCCTCCACTTACTTGGTAAGAATAGCTTTGACTATTACACGAAGCTACTATTTAGTTTTTAAATGTCC	G/A
5	PERL_886324_Ler	GAGACCACCACATCATTAGCATTACAATTCTGTAGTTGGACCTCTCAAGAAACCGTCGTAGCAGAGGGAAAAGAAACATTACACCCGCGAGCATGTTAGG**[T/C]**GACAAATAGGAAACGACTGCTATTATAAATAGAGAAGGTGAGTGCATAGCACTCCCAAACCAACTCTTGATAGTTCTGGCAAATGCTGGAGGATCTTTCC	T/C
5	CER440051	ATGCCTCTTCTCTCTTGTGAACAAGTACCTGAATACGATTTCATGACCGAATCTAACTATCACAATGAAAATGCCAATGCGTTGTCAGCGGGACAGTCCT**[C/T]**ATTTACGGAAGGCGGTGAGATGACATTTATGGTTTCTAGCCAAGAGAGAGCAGGTCAAAGCAATGATCAAATCAGGCGCGAGGATGATAGAAGCGAGACC	C/T
5	CER477932	ACTCTTTAACTTGATAACAGCAAATTCGTCCTTATTAGTAGTATACTAATGTTTTGCTATTGCTTTTGCTTTGGCCAAATGGGCCAGCAGTTCAAATGCG**[A/G]**CATCAACAAACGGGACGTAGACTACCACTAACCACTTTACCTTCTTCATCTTCGTCTTCTACAAGGAGTCCCCGAGAAATATCTTCTTCTTCGGAAGATG	A/G
5	PERL_891788_Ler	CTCACTCTTTCGTCTGAAGTTTCAAGCTTTTATTTTGAATTTCGTTATCTGGATTCGTTTGATTTTGATGTTGATGAGATTTCGCGCCTAACAGGTATTG**[T/G]**AACAGCTCGACGCACATTTCATGGAGCTGTGGCTCTTTCACCGGAGAGCTTAACGGAAGAGTCTCCCAAGGATACTGTGAAAGGATTGTTAACAACCAAT	T/G
5	PERL_894000_Ler	GCAATGGAGTACTAATCTCAATCGAAAAACTTAAAGAAGGGCACGCCATACTCATATACCGTGGCAAGAACTACAAACGTCCTTCATCGAAACTGATGGC**[A/G]**CAGAATCTTTTGACGAAAAGAAAAGCATTGCAACGATCTGTTGTGATGCAGCGACTCGGCGTAAGTGAAATGAATGAAAACGTATCTATCTCTTTCATTT	A/G
5	CER483050	TGTTTATTTCGCGATCGATGAAGATTCTCAGATTTTGTTCAGACGAGATGCGTCTACGAATATGTGAGCGAGACTTTTTCCTTCCACAAACTAAACTTAT**[C/T]**GGGCTAAGATCAGCTTTAGTGGGCTTAAAGGATCTTTTATTGGGCCCAATATATGTCAATTCTTCATTTTTTGATAAAATTTCTGAAGAGGTAGTGACAC	C/T
5	PERL_899013_Ler	GGTTATTTAATGTGTTTTAAAGTTTGACTATGAAATTTATGCTGATGTCAGTTGGTGGCACATGGTTTAAACTTCTAATATACAAGGTTTGGTTCGATAA**[G/T]**TGGACTCCATATAGGCGTCCATTAGATGGCTCATTAACAATAGACTTGATCTTACAATCGGACTCAAACATGTAGTCAGTCGACATCGACTACAGTGGCT	G/T
5	PERL_902420_Ler	AAAAGTTTCTTCCCGACACTCCCATTTGATCGGAGGGCTTCGACATTGGTGGGCTCTGTTTCGCCGCTCCGGCTAAAAGTTAGTGTAAAGCAAAAATGGA**[A/G]**CGGTACGGTGGCGCCGGAGAAGATGAGTCACGGTCGGATCCATCACATGAATGGTCCGCTCAAGGAACCGAAACTGGTATCGAAGGTAACGTTGATTATG	A/G
5	PERL_905180_Ler	AATGGCTGATCTCGATGTTCCTCCTCAAGTTCCTCAAAGTAAAACTAGAGACCTTGACAAGCTTCTTCTCCGTCATGGGAATCTCGTTGACCCCGGCTTC**[G/T]**TACCCGGACCCGGCGTAAACGCCTTTTATCCCTCAATTCGCTTCTTCGGTTATATTCCGTATCGTTTTTGTGGGTTTTCTGAGCTAAGTTTGTTTCTGTA	G/T
5	CER483565	AATGGTTATAAGGAAAGAGTTTTCGATGGTGATGAAGGAGATTGGCGTGGGGATAGGTCTTATGTGGATAATGGTAGGGGAATAAATGGGACCAGTGCTC**[A/G]**CGAAGGAAGGAGCCAAGAAACAAAGAGAGAAGATAGGTAAACTTTTGTTGAAAACAATGCAGTTGTAGACTTCACATTGGATTTCAAACTTTTAGTGTTC	A/G
5	PERL_911371_Ler	GGCAGGTCTCTTGCTCTTGGCCCACAATAAGCGTTTGCATATTTTGACCTCAAAAGAGCAGCAACAACCTTTGATGTAGCGTGTTTCGAAAACCTGCTAC**[A/G]**CGTTTCCACATTACAGAGATGATCAAGCTTAGCTTTCCTCACCTCATATTCTTCTGAATCACCTACTTTATGTGCAAAGACTCTCCAATCACATATTCTT	A/G
5	PERL_913730_Ler	GCGTTACTAAATTAAAGTGGTGTATATAAGAGTAATAGTAGCACAGAAGGACCAAGATGCAAAATAAAATTGGAAATCAAGACTGGGGAGTGACGCACAC**[T/A]**TAAAAGGTACGGACAAAACGATGCATTAGATCTTGATTGTCCGTACAACTGAATTATAAGGGTTTAATTGCAATTGAGAAGGTTTATAATAAGTGTCTTT	T/A
5	PERL_916785_Ler	CTATGTAGAATCTAGAAAGAAGTAACAAAATTAGAGGAATATAAAAGTGGATACAGCAATTGTTCGTGCGCTTGACTTTATCTGGGAAGGGAATGAGTTT**[C/T]**GGGACTCGTTTGAATCTTCTAGTTGGGTATCAAGAAAATCATCTTCAATAATATCTGCGTTGCTTAACAAAACCAATATGTACTGACTCAGAAGGAATGT	C/T
5	CER434838	AGACCAGCCAATATGGACTGCTTCTGCAGGTGCGTCCCATTCTTTGTAACTTCAATTCATCGATAGGACTTAAATTGGTTGGATTAATGAATCCTATTCG**[T/C]**CCTTGCCTTTGTCAAAGTGATGTTAGATGAATGTGTCCTTCATAGTTAACTAAGTGGTGTCCAAGAAGCGAGATATTGTGAACTTGATCTGGACCCATAT	T/C
5	PERL_923014_Ler	AGAATACCCCGAACTCGATAGTAGTACCTCCGCGTTACGTGATTGCGTTACGGGGAACTGTCCCAAGTGATGTGAGTGATTGGATACATAACAGCCGTAT**[T/C]**GTACTCGAGAAACTCCATGGCGGGGGTAAGCATATGCATGTCATTAGAAAAATCTATTCTTTGGTGGCCAAACACGGAAACACAGCTGTCTGGATCGCTG	T/C
5	PERL_926808_Ler	ATAATTTAGTCATGAGACTCTTTTCCAATAATTTGTTTTGCAGAAAACTTGTTGCAAGAGAATTTGTCACCTCTAATACTAAATGAGTACACCAGCGCGC**[G/C]**AACAATATTTACTCAGTCAATCTTCAAAAGATGATAGAAGATTGTGTCACGAAGATAAATGTTCGCCATGTGAAACGTGTAAATCACACCGGTCAAAATT	G/C
5	PERL_930173_Ler	GATGCCAAGGCCTTGATTCCGTGATCTGAGACCAAAGTATTTGAGATGTCAAGGTCATTGACTGAGTAGCAGTTCTTGGCTACTGCGACAAGGCTTGCGT**[T/C]**TGTGATGTTCTTGCAGCCGTCAAGGTTAAGAGACTCCAATGTGCGTCCGTGGCAAACAGAAATTGCAGAGACTGTGTTGTCTGAAACATTGATACATTCG	T/C

5	PERL_933560_Ler	CCAATTTGTTTTAAGATGAAAACCTGTGATTTTTAATAAGCATTACAAATATTTAAGAACTAGGGTTATATAAAAATACATCGTATACGTACATGCGATA**[T/C]**TGCTTACATGAAATAAAGCAATTAGGTACAAGAAAATATGAACTAAACAAGACTAACTAGGGAATTTTGGGCATATGAAAAGATGATCTTAGCTTCCTTC	T/C
5	PERL_937585_Ler	CGGTTCGATAAAGGCGACGCGGTGGTTATGCTTCATTACGCTTATGGCTCTGTGAAGAAATCTGTTGAAGCGTATGTTACACGCTCCGGTGGACACGTCA**[C/T]**CGAGGTACAGTTACCTTTTCCGGTTATTTCGGCGGATGAGATTATTGACCGGTTTAGGATTGGTTTAGAGTCTGGTAAAGCGAATGGTAGGAGAGTTAGG	C/T
5	PERL_942083_Ler	TATAAATTATCTAGGCAAAAAAAATAAAATAAAATCTTAATAAAGTAAAAGTAGTTTTGTTTTGTCATCTAGCGACTAAGCATTCTAGGCGCTAGGCGGA**[A/G]**GATCACTGCCGATTTAGCGCCTAACACTTTCTTAAACACTAATCCAAACCAATGATATATTAAGTCTAAAACATGAATGCAATTGGCATAATTATAAAGC	A/G
5	PERL_946009_Ler	TTAAACCCGCTAGTTAACCATCACATTTGGCCTTTGCCATTGTAGCCTCACAACTTAGGCAATTTTAGGATTTGATTGGTTAATAGATAGGTGCAAATGC**[A/G]**AGATCTTTTGGTAGCAATGGTTGCTGTTTTAAGAGCTATAAAAATATTTATTTGATTGGTATAAGAGTTGAAAATATGTGTGTTTGTGGGATGTTGTTAT	A/G
5	PERL_949882_Ler	AATCTTTGTCACCACGGCCACTGCAGTTCACTACCACCTTGGCTCCATCACGAAGAGTAGGCACGAGTTTGTCGAGGAACGCGAGCGCGTGAGACGCTTC**[T/C]**AATGCCGGTATTATTCCCTCCAATCTGCTTAATCGCATGCACGCTGCAGTCGACAACATATAATTAAGCTTAATGTTTTATGTAAATATATTAAAAAAAA	T/C
5	PERL_952964_Ler	AAGACCGAGTGCGTGGGAGAGGGATTGGAGACGACCAAGTGCGTGGGAGAGGGATTGGAGACGACCGAGTTCAAGGGATCTGTACCATGTGTTATATCGC**[A/G]**GACCTTGGTTTGTAAATGGGTGGATAGTCACAGTGGATCAATGGAAACCAAACCCAAGACCGGATTTCTTGCAAAAGATTCTTTTCTGGGTGCGCATTAA	A/G
5	CER477084	GAAGCTTGGGAGAGATTGATCGTGCTCGTGCACTCTATAAGTATTCCTCCCAGTTTGCAGATCCAAGGTCCGACCCTGAATTCTGGAACAAATGGCATGA**[G/A]**TTCGAGGTCCAGCACGGGAACGAAGATACCTACAGAGAAATGCTCCGTATCAAGCGAAGTGTTTCCGCAAGCTATAGTCAGGTCTGCATCAGTTTCTCTT	G/A
5	PERL_960485_Ler	CCAGGTGGGGCAAGTCTGGCAAAGGCGTGGCACACTCCGGCGAGGTCCGGCGGGTGGTCGGGGCGCATTGGCAAACTCTGGCCAAGTGATGGCGCAAATC**[A/G]**GCGTGCTCTGGCTAAGACTTGGCATGGCTGGGCCGGCTTTTCCTGGCGGCACCGGTTACTCGGGCGAGTAGATGGGCCTGTTGTCACGGTGCCTAGTGAC	A/G
5	PERL_962058_Ler	TGGTGCTCCTGTTATATTGCTTAGGAATCTTGATCCGAAAGGAGGGCTTTATAATGGGACCAGGCTACAAAAACGCAACTTACCAAACAAGTTGTGCAAG**[T/C]**GAGAGTAATAACTGGAGATAGAATTGGTGAAATTGTCTTGATTCCTCTAATCAACCTTACACCTTCAGATATGAAGCTGCCATTCAAGATGCGAAGAAGA	T/C
5	PERL_964521_Ler	TAGGAAAATGGGAAAGAAGAAGACAACCCTAATGTCGACTACCAAGATCGCTTGGATAGTTTACTAGCCCTGCCAGGACGGGAACGACTTCCACTCCTGT**[T/C]**TGAGAAACCTATCCCTGGAATGGAGACTCTCTAGTAATTGCGTGAGCTTTGTTTAGTTTAGTTTAGCATAGTTTGATTGTTCTTTTAGATATCCTTAGTA	T/C
5	PERL_965006_Ler	CAAGTAGATTTCATGAGATGCCATAGGTACCAAAATGGTTTTTCTTCCTTTAAACTCAAAGCTATATTTGTTGGTAAACCCATCATGTATCACTCTCCGA**[C/T]**CAAATTGCCAAGGTCTTCCTAACAAAACGTGATTAGCTTCCAAGGGCAGAATATCACATAAGACCTCATCCTCATACTTCTCAATGGCTAACGGCACTTG	C/T
5	PERL_968445_Ler	ATCGGCTAATACTACATATCAAGAAGACTAGTAGGAGTGTCAGACGGTCGAGCCCGCGCCCAAACGGGCGCTCCGTGGCAGACCTCGCTTCTCTTCATTC**[T/C]**GCAGCGGTCCGGTCCGCCAAGGACCGCGGGCGACAAAACGAAGCCCATACCCACCCCGCATTGCCCATTGGTCTAACGGGCCGGCCCACAGACCATGACC	T/C
5	PERL_972440_Ler	CCTCGAGCTTCATCATTTGATCCTCGGGAAGTGATCTTCGAGCTGAGAGAGCCAAGGAGAAGGCAGAGGAGAGCATCCAAACGCTCGACAACCTACTCGA**[T/C]**CGGACATGACGAGCAAAATCCTCCCGCTTCAACATCAGTTCCTGCTTATACTCAAGAGAGTATGGACGATTTCGTCTCCGTCTACTTCACCTGATCACTG	T/C
5	PERL_975061_Ler	TGTGGTGGCCTACCATTATATGAGTGAGAAATCACTTGTGTAAGAATCGGATGTTTGTGTTTGGGTTAGAAACCCCATCGGAAGTACACCAGCAACGACC**[A/G]**ACCGTTTGAAGAATCGGAGAAAAGAAATCAAATTGAGTTGGGATCGAGACAAGTCTAAAACAAGCATACTTCAGGGAAACAGGCAAAAGAAGGGAAGTTC	A/G
5	PERL_976568_Ler	CCCTGCTTTTTGCTGCATTATAGAACATTTTATTGTTCTGGTCCCCTGCTGCCATCCAGTGAAGCTTTGCTTTCTGTCGAAGATATTTTTCTTCGATTTC**[A/G]**GACATCCTCTGCCATCTCGCATATGCCACTGCCTCTGCATGCACAGATTGAGTTGTTGGAGCCATCATTGTTGCAGTTTGTAATTCGCAGAGCTTCTTGT	A/G
5	PERL_980169_Ler	TGAGGATGAGGTTGTGAGTGACACGGATTCGTTGAGGTTTCGTTGGCTGGTTAACGCCGTAGTAGTAGTCGCCGATCATTGGCTCGTAGAAGTAACTGAC**[G/A]**CGGCGTTTTCCTCCGTCGGCTAAGCTCGCCATCGTCACTTTCTCTGCTTCTTCTTTTTCCGCCAAGAAACGAGATATCTCCGATTTGATTTTATTGCTAC	G/A
5	PERL_984734_Ler	TAAGAAAAGTGTACGTTACATATGATGGCACTTCAATAAGGCATATGAGGGTGGACTATGATAAAGCCGGTCAAGTGGAATGCTATGAGTACGGGGACAA**[A/G]**ACCGGAACACAATATAAGGTTATTCTAATAAAATATTTGTTTACTATTGATATATTATTTTGTTTAAACACGTGGATCACTAATATATAAAAAATGTTTC	A/G
5	CER431735	TAGGACACCCTTCAAAAACCAATATTTAATAGAGAAACATATCAAAAATCTACGCTAAAGATTTAATCTAACCAAGGACCAAAGTTACCTTTGGCTTCCC**[A/G]**GTACTTCCACTGGTGTAGAGCAAAAACAGGGGATCTTCTGCATCAACCCATTCCACCTCACACGATGTTGGATATTGAGAAATAACATCCTAAAGTGATC	A/G
5	PERL_993253_Ler	CAATTCCAAGTAAAAGTAACAGATGGCGAGGAAGAAGCGTTTTTGGTGCTTTTGATTGGTACTTTATCCTGGACACTCCATTGTTGCTGTTGATGAGAAG**[A/C]**CTTTTTGGTGCTTTTGTTTGGTTCTTTTGTAGTTATCTCGGTAGCAATGAAAATCTGACGACAATTTGGACAAAGTATAGCCTTGTTAAGATTCCAATGC	A/C
5	PERL_998723_Ler	AGGTGGTTCACCATGTCTTCGCCTATTCCTCTCACCCCATATGGTGTGAACCGCTGCTTGAAGGAGATATCGAGCAACGAAGCTCTCAATTCTATCGCGC**[C/G]**AGTACCCTGAGACTGTAGCGAGTAAAGAAGACCAGTTTGTAGAGAAGCGAGATTGATGGTCTTTGGTTCTTCACAATTTACTAAATGACTTTATCAGGAT	C/G
5	PERL_1004314_Ler	GGCCCAGTGGCCCATTTATCAGATTTCAAATAACAGATCTCAACACTAGCATGGCTACACACGTGTCAGATTCAATGCATCAGTCATATCTTCAGCATCC**[A/G]**ACACTTGTCAACCTTCCATTGGATCTCTTAACTCTACGCCTCGAAAACAGTTTTTATTTATTTATCATTCCATTTCTCATTGTATCTTCATCAGTCTCTT	A/G
5	PERL_1008140_Ler	TGGGTAAAAGCCGATTAGGTACGGATTCTTTGGGTAGAAGAGTTTAGAACTCAATATATTAAATAAAAAATATATGTTCGGTTTTGGTTCGGGTCCTTTC**[A/G]**GGTTTAGGTCGCTTTGTATATAGATTTCAGAACAACTTAATAAATTATCGGATAAATACCCATATTTTCGGGTAAATACTCGCATTTCAGAGTAAAAGTT	A/G
5	PERL_1011779_Ler	AAAAAAAAGATGAAGACTTTATGACAGAGATACTTACAGGAAGTTCATCAACATCTAATTTGACGAAATCAACATCATTGAACTTATCAGCCATGGCATG**[A/G]**ATCGCAGGCTCAATCATCCTACATGGTCCGCACCATGAGGCCGAGAAATCAACCACCAGCTTTATTATTCACAGAAACCAAATCAGTTAAATTCCATTGA	A/G
5	CER438753	GGCTTCAGGTTGAAGACGACCAGTGCAGATTTGGTGAACCGTTGAAAAGAGCGGAAACATCTCCAACCATCCACAATGTTTCAACACCTCGTAGACCTCT**[C/T]**TTGCCGTCGATACCCCCTTTAATAAGCAACAGTCAGACATAAGACACAAAGAGAACCACTTTCTAATTTAGGACTATAATCCTCACAAGCAGAATCGAAG	C/T
5	PERL_1018094_Ler	GTCAAGAAGAACAGTTGTCTCTTCTTGGAAAGGTGATGAATGCTGGAGACGATCTGGTCGGTTTAGAGCAACTCGAAGAAGACACAGACGGAATGGCTCT**[A/G]**CAAAAGGCGAGACGAAGCATGGGATCGATGAGCGTAATGTCCGGTTCAAAAGGAATGGTCTACATGGAATACAACTCAGGCCGTCACAAATCCGGTCAGC	A/G

5	PERL_1022802_Ler	TACACAATATCACAGATTCATTTTAGAAAACAAACCACGAAAATGTGTGGAAACAAATTCAGATGAATGGAAACGTTAGAGTAACCGCCGAGTTTACCTG**[A/G]**GCGCGCAATCTGACTACTTCCTGATTCAGAAGCTCATTTGTCTTCTTCAAACTCTCAGCTATACTTGTTGAAAAACCAAGACCAACGTTTAAAGGAATGG	A/G
5	PERL_1026634_Ler	TACGGCCTAACCGCCGACAATCTTCCACCTCCGTCAGCGAGTGCAAAGCTCCTTCAATCCACAACTTTCCAAAAAGTCCGTCTTTACGGATCCGACCCGG**[C/G]**TGTAATCAAGGCCCTAGCAAACACGGGGATCGAGATCGTCATCGGGGCGTCTAACGGCGATGTACCCGGTCTAGCATCGGACCCGAGTTTCGCCCGAAGC	C/G
5	CER441019	CACTTTCATGGTTTCCTCTGAGAAGGAAGAAGTTTTCAGGGAACTTAATCTTGTAAGCAAGTAAAAGACAAATCGTTTCGAGGCTTTGCTTGCCGCGATC**[G/T]**ACATAATCTCCAAGAAACAAGTAGTTTGAATTAGGAGGGTATCCGCCATGTTCGAATAGTCTCAAGAGATCCGGATATTGTCCATGAATGTCCCCTGTTT	G/T
5	PERL_1037843_Ler	ACACAATTCTTTCGATCTTAGCCGTTGATTACCCAGTGAATAAGGTGAGCTGCTATGTGTCTGATGACGGTGCATCAATGCTCCTGTTCGATACATTGTC**[C/T]**GAAACTTCCGAGTTTGCAAGGAGATGGGTTCCGTTTTGCAAGAAGTACAATGTTGAGCCAAGAGCTCCAGAGTTTTACTTCTCAGAGAAGATTGATTACC	C/T
5	PERL_1042398_Ler	ACAAGATCGTAAACGAAGAATGCTTGAAGATAACTACCATGCCACGCCGAATTCTTATGCAATCCGTCAATTATGCACGTTCATTGGATGTTCTCTATAC**[C/T]**GCGGATGATGTTTACAACCACCGCGAAGGAAAACTCAAAGAGTATATGAGGCTTTTGCTCGTAGATCCTATACTTCTTTAGGACTTGGGATGCATCATGT	C/T
5	PERL_1048451_Ler	TCTCTACTGACGATCTCAAAGGAAAAAAGAGTGGAGATGCAAGGTTTTATTCAAGATGCTGCCCGTGAATTCATCAACCAGACTTCGAGGCGTCGCAGAC**[A/T]**CTGGGAACCATCAAGAATCAGATTATTACTAGAAAATGATAAATCTAAGGTAGGTGATACATTCTGTTATCTATAACAATGTTTATTTAAATTGTTTAAG	A/T
5	PERL_1054488_Ler	TAAACTAATTGTATATGTATGTGAATTGTGGGCAACGTAAAAGAAATTTATGCAATTTCCGTACTACCTAACTTGATGTTGGGAGATCGATGAGAGCGAC**[T/C]**CAAACTGAATCATTCTCATTAATCTTCCAACACTTGATTATTATCCTCAATATAAACAATCACGTTAAGAAATTGCAAACTGTTTTCTTTTAAGAAAATT	T/C
5	PERL_1058363_Ler	CATTGCATCAAATTTTTCTGACCCGATCACTTCCAGTTCCGGACAATAATCATTTTTCAAAACCAGACCCAACTCTTCAAGTAACAACCGAGCTTTTGTC**[A/C]**GACTAAAGCAGCCACAGACTTTAACGAATTTTTTCTTCAATTTCGAGTGGTTGTTCCGTTCAAAATCGAAGCCTTTTTTCATCTGTAGACATGTTTACAG	A/C
5	PERL_1060855_Ler	CCTTCAAATAATGTAGACGAAGGACTTGTAGCTCTTGAAAAGGATCAACCCCAACACCTTGAGCTTTCTCTTTGTTTATTAATTCTGTCATCTTAGGCGA**[G/C]**GATTCCACGCTTAGAGACTCGAGATTTGCAGCATACATCAGCCATGTCAAATCCTTTAGATGTATGCATGAGTTTATTACCACAGCTGAGAGATCCTTGA	G/C
5	CER436994	ACTCTGATTGTCCTGTGGTTGAAGAACATAAACGGGTGTTTTAGTTGTGAGTTGAGTACAAGTTTTGTTACAATGGAACCAAATCAAGACGTTGCAGACG**[G/A]**CGTTGAGAGTGAAAGATTACATTCTGCATCTAACTGTAGCAGCCTGAGCTTCCGGCATCATATGGGGTTGTACGCCTTAGTTCATTCAACTGAGTCTGAA	G/A
5	PERL_1067562_Ler	CCATGAACTCAATACCTTCTTTATATCTTCCAACTCTCTCATGGAACAAGCGGTTCTTGAGACGGATTCTCCTGTATCTTTCTTCGTCTGGCTTCTTCGC**[T/C]**ACGTTCCTCACAATTGTCAGAAGCGTTTCAAATACCCTCCTCGTGATTCTTGGATCTTCATCCTACAAAAACAAACAAGATTTCATTATCTCTCTTCTTT	T/C
5	PERL_1070916_Ler	AGATCGAGCAAGAACAAGAACAAGAACAAAAACAAGAAATCTCTCAAGCATCATCATCATCAAACATGGCGAATCTAGTTACGTCATCAGATCATCATC**[C/A]**GTTGGAGCTAGCTGGAAATCTCTCAAGCATCTTCGATACTTCATCTTTACCTTTTCCTTATTCTTATTTCGAAGATCACTCTTCTAATAATCCTAATTC	C/A
5	PERL_1073332_Ler	TAGCATCTGAAATGAGTCCAAGCATTGTAGCAAATATCAACATACCACCTGCACTTATAGCAACAGAAACTATTCTTGCTCCAACACCAACTCTATCAGC**[A/G]**TGACTTCCCGAATCCGCTACAAAAGTCCACGATAACCAAAGGGCTTCATCAACCCCACAGTCACTAACAGCATACAATGCTAATCCACCATAAACTATAA	A/G
5	PERL_1075675_Ler	AAATCCAAAACCAATCAAAGGACGCTCTTGTTTTCTGAAACAACACGATTAGAGAGATCTTCTTATTTATATTAACTCTCTTGGACACTTTAAACGGTTT**[T/C]**GCACGATTTGGTTCTAGTTCGAACCAGGACGAATGGTTTTATAAATTTACAAACCAAACCGATGAAATTAGTTGTTCTCTTCTTGAGTAATCAACTAATC	T/C
5	PERL_1078160_Ler	TGCAATTCGACAACATACCCCGACAAATGCTACAAGTCCTTATCCTCCTACTCATCGAATATCAAATCCGACCCGATCAAACTATGCACCACCGCGCTTA**[A/C]**CCTCAACGTGAAATCCGCAAAGGAAGCAACGTCCGTTGTCTCTAAGCTTCTCAAAATGTCTCAGAAATCAACCGCCGGACGAAAGGGTAAAATGTTACCA	A/C
5	CER426941	GACTACACGCTTTACAATGGCTCTCTTATAGGCGCGTCAGGCTCCGACGACGGAACGGTTCAAATGTGGGATCCACGTAACGGAGGGACGTTAGAAGAAA**[C/T]**GGTTAGGCCCGGTGGTGGAGCGGCGATTTGTTCGGTAGAGTTCGATCCGTTCGGTGGATCTTCCATAGCCGTTGGATGTGCTGACCGGAATGCCTACGTG	C/T
5	PERL_1084153_Ler	AATAGTAGTATACAACAAAGTATCGCATGATTATTCTCCTAATCTTCGCATTTCGTTTCTACACAAAAGTCGCCGCAACATAACTCATGCAACGACAAAG**[C/A]**GGGATCGAGGGATAGAGAGAGCACACAACACAAAGAGCTGCCTTTGACTTTTTCAAACCCTAAACCAAATTGACCGTAACGTCCATCAACAACACGTCCC	C/A
5	PERL_1086348_Ler	TCTCACACTATATCTCAAATCACAGAAAACAAAAATCTTTTTTTAATATACAATTTAAATCTTTTACTCCGATTTAAATATAACATTTTTGGGACAACTA**[G/A]**CCAAACGTTACAAGACGTCAATAAATTCCATTGACGGTTACAATTTGATGATAGGTTTGCTTTCGTTACCCTGAGTCCCTGAGTATTAGTGTGGATATCC	G/A
5	PERL_1088463_Ler	TCGTGATTTTGAATCCTTATGAAGATGAATGCACTTCTTTGGATAAGAAGGTGCCTATGGCGTTGTTGTATGATAATGAACTTGTAACCATCGGTTCATC**[C/A]**CATGGCTGGATAGCTACTTTGAGTCAGGACGACGGACTACTGCGTCTCCAAGACGATCTAAACCCGGTTGCATCGGATACTAACCCGAAACGCATTCCCC	C/A
5	PERL_1091480_Ler	CACGACAAATAAAATCAATAAGTAATTTTCTTTTAATCAAAATCACGCCTTTGCGTCTCCTTGCATCTCGCCTGAATCATCTCAGACCGTCCCATGAGGC**[A/T]**AAGCGATGGATTCCGTCATGGTTTGTCACGCATATGCAGTTTTTATTTTAGTTTATATTTTTTTGGTAAAAAGTTAAATTTATATCATTTTTGAAATTTA	A/T
5	CER441642	TTGGACTTTTCTCTTGCATATTGCTTGCCTTCGTGAACCAGTTCTTTGGTTTCCGGTCGAACCAGCTTTGGGTATCTTCAGTTGCGGCACAGATCGTGAC**[C/T]**CTTCCGCTAGGGAAACTCATGGCAAAGACGCTACCGACAAAGAAATTTGGGTTTCCCGGGACGAATTGGTCGTGGTCTTTTAACCCTGGTCCGTTCAATA	C/T
5	PERL_1096052_Ler	TCATTACCCTAAAGAAATATGAATTTCCATAATTCAGTTCTTTCACTCTGTTAGCAAAGACAGTATAAGGGTCTCTTTCTCGAGCACGGACTGATTCTTC**[A/T]**GCCATTTGCATCACTATTCCAACCTCTTCTTCTTGTGGCATCACGTTTGTAGTGAAAAGTTGATATACAAGCTGGAAAGAACGTATAATAAGGTTCAGTT	A/T
5	PERL_1098054_Ler	ATGGAGCTTGAAATTGAAATTCGATGAGGAAAACAAATCGGAAAACAGTAATCTTACTTTGAGATCGTATTCAGCATCATTCGCCGGCATTTTCGAAACG**[G/A]**CGATGAGAGCTTCCACCTCCGGCGAAAACGGCGGCGTGAGCAAACGATGAAACAGAGAATCAATTCACAGGAACAGATCTAATCTAAGCAGAGAGAGAGA	G/A
5	CER436560	CAAAGGGATAGACATTAATGGGCTTAAAATGTATTGGGCTTTATTTATTAGGCCCATTAAGCACCGCCGAACGTCGTGACATTGTTAGCTAGCTCGCCGG**[A/C]**GCAAAATTCAGTCTGTGAAACTTTCGGAGAAGAAGGGATGTCGAGAGGATCTCGATTCGTTAGGGCTCCTGGGCTCTTACTCTGTCGCGTTAATCTTCAA	A/C
5	PERL_1101746_Ler	GAAGAGTTTAAAGATTTATAAAGAAGAAGACCGTACAGATGGACAGATTTAGCTGAAGGCGTTACGACAATATCACCTGCCAATTGTTGTGAGTTTGCGA**[C/G]**TCACGAGAGAGATTAAGGATTCAGGATTAAGGAAACCTATACATATGTTTTTTTCTTTTTTTCATTATTAAAAGGTATACATGTATAAATATGTTATCTT	C/G

5	PERL_1105321_Ler	ATTAGTACCTTATCAATATAATGAGAGTTTTTCCTCGGAACCTCCTCAACGGACCATCCTCTAGGCAATCGGAAACCTTTGGAGGCATATTCAACTCCGC**[A/G]**GTG ATCCTGCAAAAGCATAAACACACTGATTTTAAGTATTTGATCCAAGAAGAACCATATGCTTCTGTAATTTCAAATCAGAACAAAGGACCTGTTTGGT	A/G
5	PERL_1107666_Ler	TTATTCACTTTCTTTCCAACATCTCTACTCTCTAGTACTAGTAAGTAGTAACACTGAAGAATTTAAAGTTAGGGGCAGAAGACTTATGCAAACACCTAAC**[G/C]**GCAC TCAGTCTCACGATTGCTCCCAACAAGCCTTCATGGACTACTGGTTCTTCTTCATCAAACAACTTCTCATAGAAGACTATACCGGACTCAGACCACG	G/C
5	PERL_1109312_Ler	TTAAAGAATTTGTTGGCCCAAAAGTTAAAGAATCACCATCTAGGCTTTTAGCCTACAAATTTGGACAGATCATAGCACAGAGAATGATTTCTCTAACATG**[G/A]**ATC GGCCAGGCCTTAATACTAAGCCAATGTGCACAATAAGACAAGTCATAAAATGTTATTCAGATACATGAATATGTTTGTCTTCGTTGGATCAAAAACC	G/A
5	PERL_1110914_Ler	GAAACACTTAATGGATGCTTATAATAAGAAGTAAGACTTTCCGCCGCTGTGGAAAATATAAACCCATAGAATTGGGACTGCTTTTGTAGAATGTGGACCG**[A/T]**TCG GACTTTGTCAAATTTCGTTTTAACCGTTGGTCCTCCTTTCGCTTACTCATTTTCCGCACGCCATTAACGATAAAGATTCCCATTATTTTTCGTCGTC	A/T
5	PERL_1112536_Ler	GCTAACCAAAATGAAACAAGTACAATGGAACCAGCAGCTTTCCAGAGGCAACGATCATGGGTACCTCCACAACCACCTCCAGTTGCCATGGCAGAAGCAG**[T/C]**C GAGGCCATTCGCCGTCCTAAGCCACAAGCTAAGATAGACCAAGAAGCAGCTGCTAGTGATGGCCAGTCAGGTGTGAGCGATGAGTTACAGAAGATCACT	T/C
5	PERL_1114716_Ler	ACAAAACAAAAATCAGAAAGACAAATCGATAAATTAGTAAAGAGCTAGAGAAGATAAACTTGTAAAGAAATAGGGTTAAGGCTTACTAGTAGCTTGCGCC**[A/T]**GAA ACAACAAGATTGAATCGAAGGTGGAGATAGCTAATTATCGTCTAGGGTTTGCCATTTTTCACTCTCCAACGGGGGAATTGGGGATATCGTCAAGCTA	A/T
5	PERL_1116668_Ler	TTTCATTGATGGAGGCACAAGAACCTGCATTGGTATCATCAGCTTCTGTCTTTTCTCGGGTTGATGATGCAACGGTGGCAGCGGGCGATCAATGGAGTCC**[A/G]**T GCAATCAGAGACCGCACTGACTCCAGATTGAGAATGATCAGTGGTAGTAGTAGTAGTAGTTGCAGTTGTTGTGGTTTGTAAAGAAGTAGAGACAAGGCC	A/G
5	PERL_1118867_Ler	GAACGAAAAAAAGGAACAACAAACTAGAGAGCGACCTTCCTCTTACCGGCGCGTTACTCTCACGCGCTAACGTACATATAGACGAGTCGAGCGATCTCGC**[A/G]**G CGGCGTTTATCGATACCCTAAGCTCGTGGTTGTTTCAGATTTTTGTTAATTGAATTCTCTGCATCGAGATAGAGGGAGATGTGGGTTTTCTATCTGATC	A/G
5	PERL_1120950_Ler	GAAGACGGTCTTGATTACACATCTATACGTCGCTCTAACTCTTTCTACGCCGATGCAATTGCAGACTGCATTGACTTCATCAACCGCTCCTCCGCTTGAC**[A/C]**GC TTCTCATTCTCTTCCAATAATTCTTTCTTCCATTTTGTAAAATCCAATGTTTCTGTATATGCAACCAATTCGAATTCTTTGTATTATTTTATTTTTTT	A/C
5	PERL_1123914_Ler	ATGCAGTGATTCCTGGGCTAATGGCTGCTGGGGAGGCAGCTTGTGCATCTGTTCATGGTGCCAACAGGCTTGGTGCAAATTCACTGCTCGACATTGTCGT**[A/G]**T TTGGTCGTGCTTGTGCAAATAGGGTTGCAGAGATAAGCAAACCAGGTTAGTCTTTTTTGAGGACTGCTGTTATTATAAAAGATAAGTCTCTTCTGATCA	A/G
5	CER435876	AAAACCTTAACCCTAAGAATACGAAGCAGAACCAAAACTTAGCTCTATGAAATCCGAGAATCGGAAACTTTACCTGCAAATCCTCGGAGGCCAGCAGAAG**[A/G]**CG AATATCCCGACATCACATGTATATAATCTCAGAAATTATACAAAAAATTCTCAACTCTCTTCGCTTTCTCCGGAGCAAACACCAAATCCGAAACACTT	A/G
5	*PERL_1126141_Ler	TGTCACAACTAGAACTAAAATTAAGGATCCATTTTAAATGTTAAAAAGAAAAAATTGGACGATGAGAGTATTAACATGCACTTACCGCATTATCAGCGC**[C/T]**ATCTT GGTTCGGATTCGGATCCGAATTCGGGTTGACTTTGCCCGTTTCCTGATCCAGCTTCTTCTTCCTGTGTTTCGTCTTCTTCTTCGTCAAGTCAAG	C/T

Supplemental Table 1 : List of SNPs used for genotyping

* Genotyping performed with Taqman technology

(1) SNPs genotyped only in female population
