# Supplementary material for: Genome-Wide Crossover Distribution in Arabidopsis thaliana Meiosis Reveals Sex-Specific Patterns along Chromosomes
Source: PLoS Genet. 2011 Nov 3;7(11):e1002354. doi: 10.1371/journal.pgen.1002354 (PMC3207851; doi:10.1371/journal.pgen.1002354)
Supplement: Table S3 — Correlation between CO rates and chromosome features. (a) positive (+) or negative (-) correlation. (PDF) [file pgen.1002354.s004.pdf]

	Coding GC			GC1			GC2			GC3		
	(a)	R2	p-value	(a)	R2	p-value	(a)	R2	p-value	(a)	R2	p-value
**Mâle**												
Chr1 Male	(+)	0.006	0.487	(-)	0.017	0.215	(-)	0.001	0.753	(+)	0.052	0.03
Chr2 Male	(+)	0.076	0.0362	(+)	0.003	0.698	(+)	0.004	0.624	(+)	0.092	0.021
Chr3 Male	(+)	0.004	0.615	(-)	0.056	0.0648	(+)	0.001	0.776	(+)	0.011	0.412
Chr4 Male	(+)	0.137	0.0171	(+)	0.019	0.387	(+)	0.083	0.067	(+)	0.097	0.047
Chr5 Male	(+)	0.03	0.65	(+)	0.017	0.235	(+)(-)	0	0.964	(+)(-)	0	0.856
**Femelle**												
Chr1 Female	(-)	0.014	0.266	(-)	0.02	0.682	(-)	0.006	0.486	(-)	0.006	0.467
Chr2 Female	(-)	0.026	0.226	(-)	0.015	0.354	(-)	0.006	0.562	(-)	0.059	0.066
Chr3 Female	(-)	0.077	0.026	(-)	0.051	0.0696	(-)	0.005	0.584	(-)	0.119	0.005
Chr4 Female	(-)	0.04	0.212	(-)	0.085	0.0644	(-)	0.004	0.699	(-)	0.013	0.479
Chr5 Female	(-)	0.018	0.223	(-)	0.032	0.105	(+)(-)	0	0.869	(-)	0.003	0.651

Supplemental Table 3: Correlation between CO rates and Chromosome features

(a) positive (+) or negative (-) correlation
