# Supplementary material for: Genome-Wide Crossover Distribution in Arabidopsis thaliana Meiosis Reveals Sex-Specific Patterns along Chromosomes
Source: PLoS Genet. 2011 Nov 3;7(11):e1002354. doi: 10.1371/journal.pgen.1002354 (PMC3207851; doi:10.1371/journal.pgen.1002354)
Supplement: Table S4 — Observed Number of chromosomes having 0, 1, 2 or more COs. (PDF) [file pgen.1002354.s005.pdf]

**Number of COs**	**0**	**1**	**2**	**3**	**4**	**5**
**Chromosome 1**						
Male	222	622	487	138	26	4
Female	540	731	218	15	2	1
**Chromosome 2**						
Male	441	710	267	40	2	1
Female	685	757	59	6	1	0
**Chromosome 3**						
Male	426	687	379	62	7	2
Female	664	724	114	3	2	0
**Chromosome 4**						
Male	533	781	260	19	0	0
Female	737	706	58	1	0	0
**Chromosome 5**						
Male	250	621	428	92	15	0
Female	603	736	162	7	1	0

Supplemental Table 4 **Observed Number of chromosomes having 0, 1, 2 or more COs**
