# Supplementary material for: Genome-Wide Crossover Distribution in Arabidopsis thaliana Meiosis Reveals Sex-Specific Patterns along Chromosomes
Source: PLoS Genet. 2011 Nov 3;7(11):e1002354. doi: 10.1371/journal.pgen.1002354 (PMC3207851; doi:10.1371/journal.pgen.1002354)
Supplement: Table S5 — Effect of the truncations on the distribution of chromosomes with 1 or 2 or more COs. (a) Based on the genetic map length. Observed loss of chromosomes with one CO. (c) Observed loss with chromosomes with 2 or more COs. (PDF) [file pgen.1002354.s006.pdf]

Supplemental Table 5 : effect of the truncations on the distribution of chromosomes with 1 or 2 or more COs

		chro 1	chro 2	chro 3	chro 4	chro 5
**Male -30%**						
	**overall loss(a)**	42.5 %	42.8	34.1	39.3	44.6
	**1 CO loss (b)**	39.7	36.9	29.5	32.0	40.6
	**2 COs or more loss**	43.6	49.2	37.3	49.1	46.6
**Male -50%**						
	**overall (a)**	62.2	54.0	51.2	46.5	61.2
	**1 CO loss (b)**	58.4	47.0	44.3	38.4	59.4
	**2 COs or more (c)**	63.8	61.3	56.1	57.3	62.1
**Female -30%**						
	**overall (a)**	27.1	38.7	20.3	31.9	24.6
	**1 CO loss (b)**	20.5	35.8	15.3	27.6	16.7
	**2 COs or more (c)**	36.8	54.3	35.1	57.1	41.3
**Female -50%**						
	**overall (a)**	50.1	45.8	43.4	37.1	46.9
	**1 CO loss (b)**	41.6	42.9	36.2	31.7	36.8
	**2 COs or more (c)**	62.8	61.4	64.9	68.9	68.2

(a) based on the genetic map length
(b) observed loss of chromosomes with one CO
(c) observed loss of chromosomes with 2 or more COs
